# Cage Alkyl Carbenes Provide Experimental Evidence for Isotope-Controlled Selectivity in Competing Tunneling Reactions

**DOI:** 10.1021/jacs.4c18129

**Published:** 2025-05-12

**Authors:** Akkad Danho, Bastian Bernhardt, Dennis Gerbig, Marija Alešković, Peter R. Schreiner

**Affiliations:** † Institute of Organic Chemistry, 9175 Justus Liebig University, Heinrich-Buff-Ring 17, 35392 Giessen, Germany; ‡ Division of Organic Chemistry and Biochemistry, 54583 Ruđer Bošković Institute, Bijenička cesta 54, 10000 Zagreb, Croatia

## Abstract

We report the synthesis and reactivity of adamantylidene (**1**) and pentacyclo­[5.4.0.0^2,6^.0^3,10^.0^5,9^]­undecanylidene (**2**) under matrix isolation conditions. The latter previously unreported carbene is persistent under cryogenic conditions and has been characterized spectroscopically. The singlet carbenes were generated through irradiation of their corresponding diazirine precursors followed by trapping the products in argon or nitrogen matrices at 3.5 K. Analyses using IR and UV/vis spectroscopy together with density functional theory computations provide strong evidence for the successful preparation of these reactive species. Carbene **1** (Δ*E*_ST_ = −3.0 kcal mol^–1^) undergoes a slow hitherto unreported but theoretically predicted quantum mechanical tunneling (QMT) C–H-bond insertion and ring-closure to 2,4-dehydroadamantane (**4**). In contrast, **2** (Δ*E*_ST_ = −5.2 kcal mol^–1^) remains unchanged under cryogenic conditions but rearranges to homohypostrophene (**9**) upon λ = 627 nm irradiation. Attempts to prepare protoadamantylidene (**3**) (Δ*E*_ST_ = −5.1 kcal mol^–1^) in a similar fashion did not allow the direct observation of the free carbene, but enabled follow-up QMT reactions, whose selectivities are determined by the ^1^H and ^2^H isotopologs, thereby demonstrating isotope-controlled selectivity (ICS).

## Graphical abstract

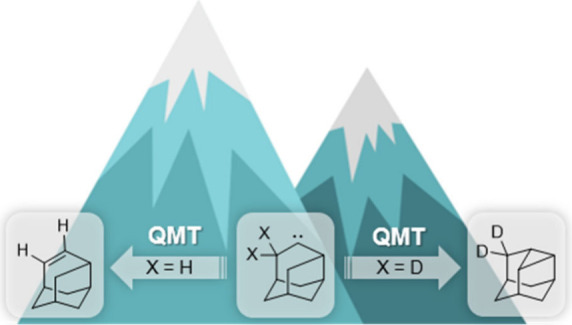

Isotope-controlled selectivity (ICS) is defined as a molecular system where one of two conceivable products—both resulting from a quantum mechanical tunneling (QMT) reaction from the same starting material—forms predominantly, only depending on isotopic composition.^1^ This novel concept of controlling reactivity has been theoretically predicted^1^ but not been demonstrated experimentally. Here we investigate the effect of isotopic substitution (hydrogen vs deuterium) in the reactivity of singlet protoadamantylidene and provide spectroscopic evidence for the formation of different products as a result of ICS. This work is organized to highlight important revelations and unexpected challenges associated with our quest of finding a cage carbene system that would demonstrate ICS, and to underscore that even structurally very similar systems can have quite varying reactivity when QMT operates.

Singlet alkyl carbenes are highly unstable, with only a few spectroscopic reports available, including di-*tert*-butylcarbene,^2^ diadamantylcarbene,^3^ dicyclopropylcarbene,^4^ and adamantylidene^5^ (**1**, Figure 1). The majority of research into these carbenes stems from computational and theoretical studies.^6^ Beyond methylene (which has a triplet ground state), singlet ethylidene represents the simplest alkyl carbene, which, however, undergoes a very facile [1,2]­H-shift; despite multiple attempts, the direct spectroscopic characterization of ethylidene has remained elusive.^7–14^ Analogs such as 2,2,2-trifluoroethylidene^15^ have been generated and characterized in noble gas matrices at low temperatures. Carbenes including norbornen-7-ylidene,^16,17^ cyclobutylidene,^17^ and tricyclooct-8-ylidene^18^ have all been investigated theoretically. Such so-called foiled carbenes^19,20^ display a combination of through-space interactions, nonclassical bonding, and hyperconjugative interaction.^21^ Apart from fast 1,2-shifts, cyclic singlet carbenes may also undergo ring expansion by heavy-atom QMT through C–C or C–H bond insertion reactions.^22,23^ Heavy-atom QMT is less common,^24^ owing to the mass dependence of the tunneling rate, which typically results in significantly extended tunneling half-lives unless the reaction barrier is narrow.^25^ Examples of reactions involving carbon tunneling include automerizations of cyclobutadiene,^26^ 1,5-dimethylsemibullvalene,^27^ and the ring closure of cyclopentane-1,3-diyl.^28^ As QMT half-lives depend profoundly on particle mass but even more so on barrier height and width,^25^ computational predictions require a suitable potential energy hypersurface (PES). Canonical Variational Transition State Theory (CVT)^29^ corrected for small-curvature tunneling (SCT)^30^ in conjunction with density functional theory (DFT)^31,32^ provides results in reasonably good agreement with experiment.^33^

**Figure 1:**
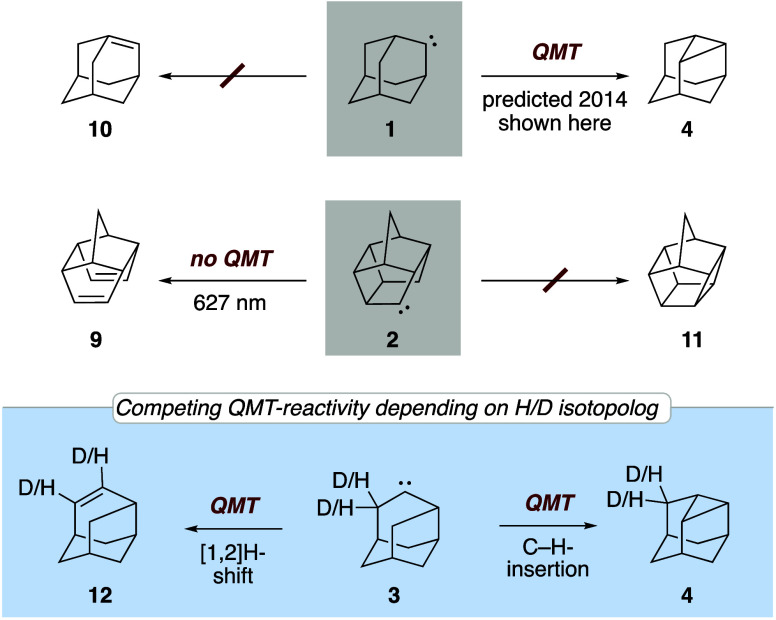
Outlining the hitherto unreported reactivities of known carbene **1** and the reactivities of novel carbenes **2** and **3**.

Alkyl carbene **1** was first isolated and spectroscopically characterized using the matrix isolation technique by Bally and Platz et al.^5^ In 2014, Kozuch et al. studied the tunneling reactivity of **1** and calculated a tunneling half-life of 62.2 h at the CVT/SCT//B3LYP/6-31G­(d,p) level of theory.^22^ While Bally and Platz et al.^5^ observed the rearrangement of **1** to dehydroadamantane (**4**) upon UV irradiation, they (unintentionally) did not wait long enough to also observe its reactivity in the dark. As such, we sought to experimentally examine the predicted QMT ring closure reaction of **1** to **4** in the dark and to take this QMT reactivity as a model for possibly observing ICS with a carbene. As we will outline, **1** proved not to be suitable for this purpose, even though it does show the predicted QMT reactivity, because alternative rearrangement pathways are not competitive. We then investigated pentacyclo­[5.4.0.0^2,6^.0^3,10^.0^5,9^]­undecanylidene (PCU-carbene) (**2**), also a cage structure, with the hope that it would undergo competing QMT reactivity.^34^ Surprisingly, **2** does *not* display observable QMT reactivity because it is unexpectedly stable under our conditions.^35,36^ We finally arrived at protoadamantylidene (**3**), an isomer of **1** that has rather short QMT half-lives but does demonstrate H/D-ICS in competing QMT reactions.

Adamantane diazirine (**5**) and pentacyclo­[5.4.0.0^2,6^.0^3,10^.0^5,9^]­undecan-8-diazirine (PCU diazirine, **6**, Figure 1) were synthesized according to literature procedures from the corresponding ketones **7** and **8**.^5,37^ Due to their high volatility, **5** and **6** could easily be evaporated onto the cold matrix window using an excess of argon as the host gas.

Our matrix-IR spectrum of **5** is in excellent agreement with reported data^5^ (see Supporting Information, SI, for matrix IR spectra and signal assignments). Upon irradiation at 365 nm, matrix-isolated **5** and **6** undergo a series of reactions, resulting in the formation of **1** and **2**, respectively, and leading to the concomitant disappearance of all IR absorptions assigned to **5** and **6**. In parallel, new IR bands emerged, corresponding to the diazo form as well as compounds **1** and **2** (Figures S21 and S22), in accord with the literature.^38^ The experimentally observed IR spectra of **1** and **2** match well with the computed IR spectra at B3LYP/6-311++G­(3df,2pd). Due to the overlapping of most bands above 1500 cm^–1^, the assignments are difficult. In the range of 1500–600 cm^–1^, cage vibrations dominate, with very weak intensities as compared to the stretching vibrations.

In extension to the previous study by Bally and Platz et al.,^5^ we found that over the course of several hours the bands of **1** gradually disappeared (in the dark) while those of **4** slowly emerged (Figure S24). The observed reactivity of **1** under these experimental conditions with a half-life of 6 h can only be explained by QMT, in line with the prediction by Kozuch^22^ and our computational data (*vide infra*). Carbene **1**, generated from the irradiation of **5** at 365 nm, exhibits a UV/vis spectrum with an absorption maximum at around 610 nm (Figure 2a). After 18 h in the dark, this UV/vis signal disappears completely, confirming the QMT reactivity observed also in the IR spectra. Only **4** was obtained, rendering **1** unsuitable for the investigation of ICS. Consequently, our attention shifted to **2**, because the precursor materials can be synthesized as easily as for **1**.

**Figure 2:**
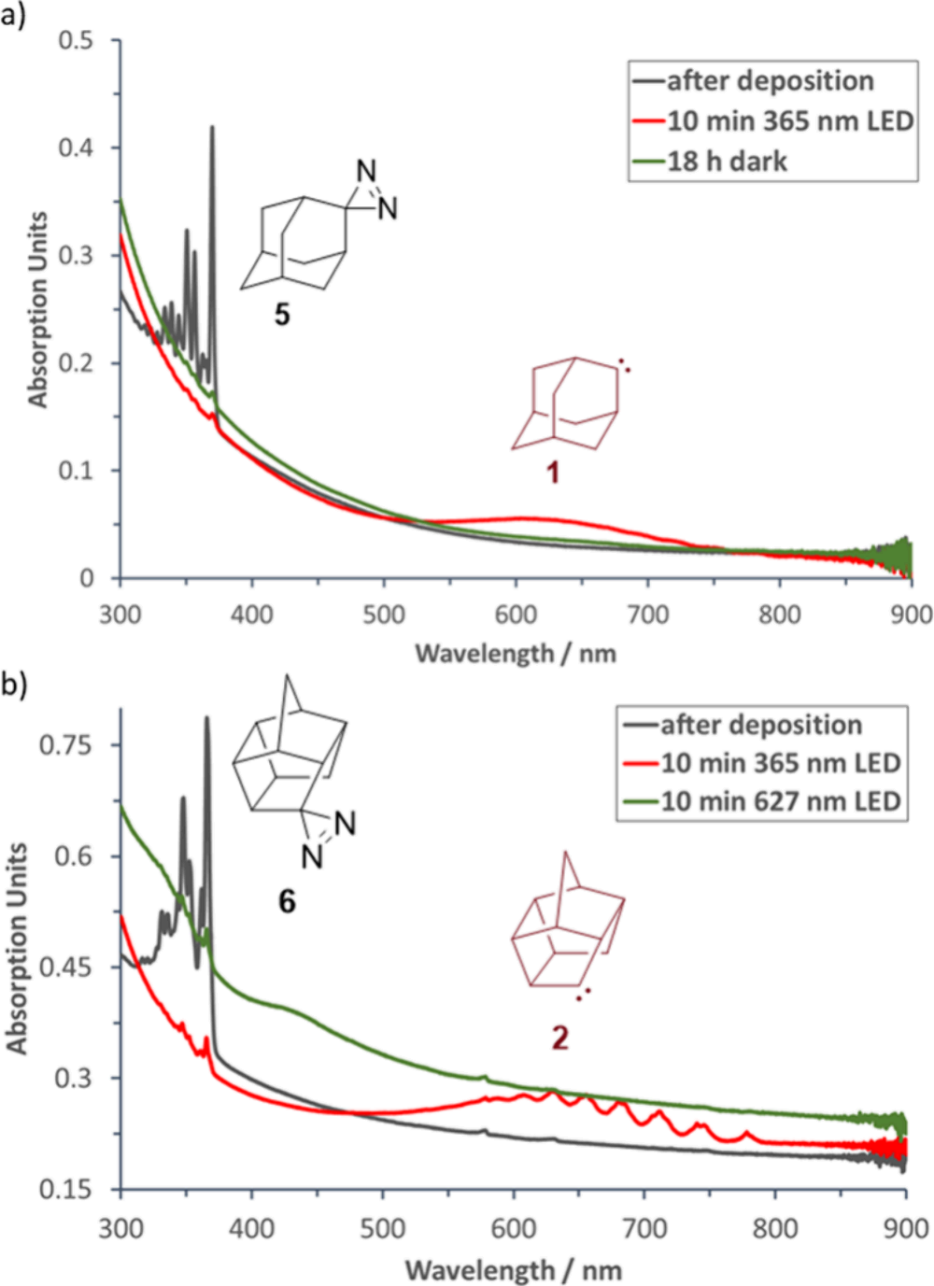
Experimental UV/vis spectra. (a) The spectra of **5** (black) and **1** (red) are in good agreement with literature data.^5^ Carbene **1** vanishes when the matrix is kept in the dark for 18 h (green). (b) Analogous spectra were recorded before (black) and after (red) irradiation of **6**. However, **2** is persistent, and its fine-structured UV/vis absorption (red) only vanishes (green) when the matrix is irradiated at 627 nm.

To our surprise, **2**, generated from **6** by 365 nm irradiation, did *not* display QMT reactivity even after several days in the dark; this strongly indicates that **2** is persistent under cryogenic conditions. Only after irradiating **2** with 627 nm light, we observed the disappearance of the bands of **2** and the simultaneous appearance of bands corresponding to homohypostrophene (**9**). This excludes **2** from experiments related to ICS. In moving forward, however, we had to first understand the QMT behavior of **1** and **2** to identify a suitable cage carbene.

To understand the different QMT reactivities of **1** and **2**, we conducted a detailed analysis of their intrinsic reaction coordinates (IRC) for the QMT pathways using the UB3LYP/6-31G­(d) level of theory (Figure 3), according to Kozuch et al.^22^ Concurrently, we employed CVT/SCT^29,30^ to compute the half-lives (τ) for these reactions at the same level of theory, which enabled us to quantitatively assess the likelihood of QMT occurring under our experimental conditions. For the **1** → **4** C–H bond insertion reaction, the computed half-life of 20 h agrees well with the experimental value. At UB3LYP/6-31G­(d) the reaction leading toward *anti*-Bredt adamantene^39^ (**10**) gives a computed half-life of >9 × 10^22^ h, indicating that this reaction is not competitive (Figure 3A); indeed, **10** was not detectable in our experiments. This also implies that ICS cannot be studied with **1**.

**Figure 3:**
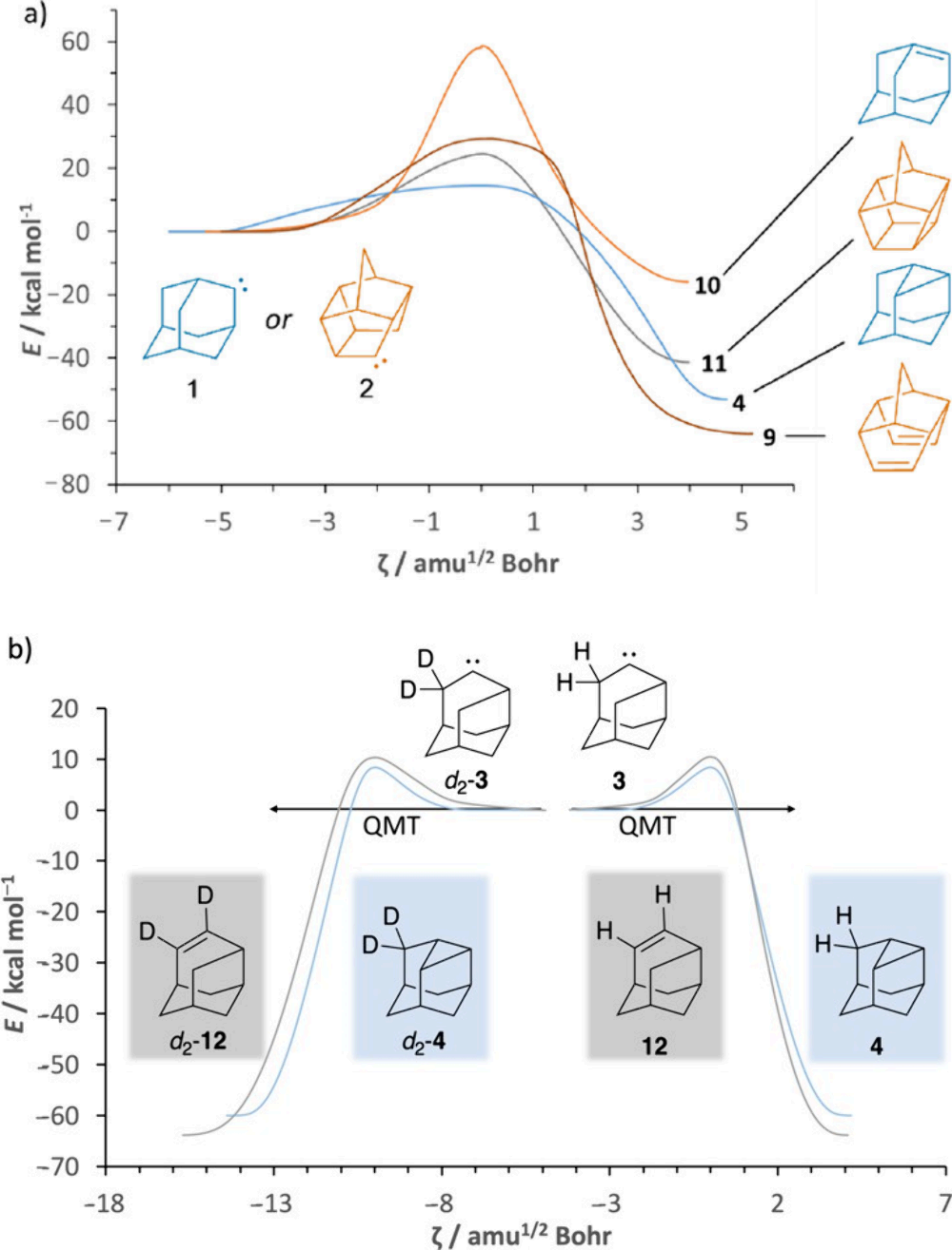
Intrinsic reaction coordinates for the cage alkyl carbenes of this study computed at the UB3LYP/def2-TZVPP level of theory.

In comparing the IRCs for the **2** → **9** and **2** → **11** rearrangements (Figure 3a), it is evident that the former reaction involves a much lower total barrier of 14.8 kcal mol^–1^, favoring the reaction to **9** via a diradicaloid open-shell singlet intermediate (see Figure S64); this energy can be supplied by irradiation at 627 nm (45.6 kcal mol^–1^), leading to **9**. QMT reactivity was not experimentally observed for **2**, in accordance with approximate closed-shell computations for the direct reaction of **2** to **9** (>10^35^ h) and no QMT for the reaction to **11** (>10^11^ h). For the two-step open-shell solution, the generation of the diradicaloid intermediate from **9** is rate-determining (>10^33^ h; see SI).

Hence, we needed a system that has a *lower* and *thinner* barrier and would show QMT behavior in the right time interval. We turned our attention to hitherto also unreported carbene **3**, which is an isomer of **1** that may show the right reactivity.

According to our UB3LYP/6-31G­(d) computations, the half-life for the C–H insertion reaction of **3** to **4** is 4 × 10^–9^ h while the half-life for the competing [1,2]-H shift to **12** is 3 × 10^–4^ h. These values suggest that the C–H insertion occurs much more readily than the [1,2]-H shift, thus favoring the formation of **4** as the dominant product in the protio-case. The extremely short half-lives of **3** in either reaction means, however, that the free carbene cannot be observed directly under our matrix isolation conditions and that we must rely on product analysis. To generate **3**, we initially irradiated the precursor diazirine (**13**) at 365 nm, leading to its expected and well-known rearrangement into the diazo form,^40,41^ as observed in similar molecules.^34^ As this intermediate absorbs at the same wavelength (254 nm) as the carbene (as judged from the computed UV spectrum), this route had to be abandoned. Instead, we generated **3** and the diazo form, via high vacuum flash pyrolysis (HVFP) and recorded the typical carbene follow-up reactions leading to products **4** and **12**, whose ratio depending on isotopic substitution should then reveal whether ICS does occur.

The determination of the product ratios of the reactions of **3** to investigate ICS is based on identifying the characteristic IR bands (Figure 4) and the corresponding red-shifted bands of the isotopolog. Specifically, the strongest vibrational signal for each product was identified and integrated. To accurately compare the isotopolog ratios, a correction factor was applied. This factor was derived from the computed intensity ratios of the vibrational signals in the deuterated and parent molecules, allowing for direct comparison between the undeuterated and deuterated peaks. The experimental intensities were then normalized using the correction factor (Tables S3 and S6), ensuring that the observed data accurately reflect the underlying reaction dynamics. The intensities obtained for the H- and D-isotopologues were subsequently used to calculate the selectivity between the competing reactions (Tables S3–S6).

**Figure 4:**
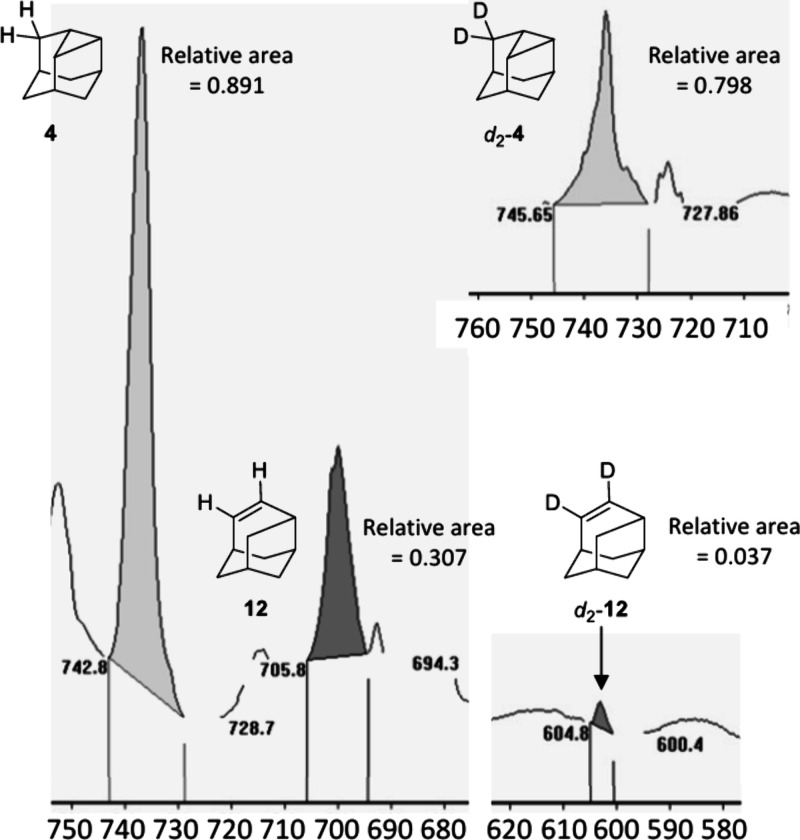
Representative IR spectra showing the pyrolysis products of protoadamantane diazirine (**13**) and *d*_2_-protoadamantane diazirine (*d*_2_-**13**), each trapped in an argon matrix at 3.5 K. The spectra were analyzed by integrating the peaks to determine the product distribution.

As evident from the IR spectrum after HVFP, (the experiments were conducted three times for both H/Disotopologs) (Figures S41 and S46), the product distribution shows a ratio of 2:1 favoring **4** (Figure 5). Substituting hydrogen with deuterium at the α-position of **13**, leading to intermediate carbene *d*_2_-**3**, changed the product ratio to 20:1, favoring *d*_2_-**4**, which was also qualitatively predicted computationally (Tables S27 and S28). The markedly different product ratios highlight the significant impact of deuterium substitution, providing strong evidence for ICS.

**Figure 5:**
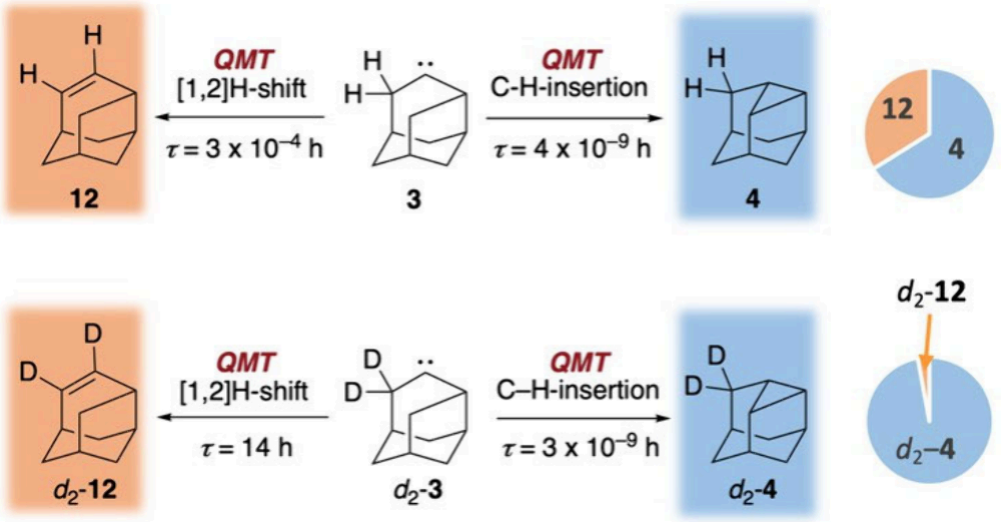
Reactivity of matrix isolated **3** and d_2_-**3** after pyrolysis at 800 °C, with calculated half-lives at the UB3LYP/6-31G­(d) level of theory at 10 K.

In conclusion, we generated three cage alkyl carbenes to test their suitability to provide evidence for ICS. Known carbene **1** underwent a QMT reaction as computationally predicted, with a half-life of 6 h, while **2** unexpectedly remained stable under cryogenic conditions but rapidly rearranged to **9** upon 627 nm irradiation. Much more reactive and closely related carbene **3** was generated *in situ* and α-deuterium substitution of the diazirine precursor significantly altered the ratio of the [1,2]­H-shift vs C–H-insertion products, thereby providing the first experimental evidence of isotope-controlled selectivity.

## Supplementary Material



## Notes

Experimental details, computational details and coordinates, synthetic procedures and characterization, matrix isolation IR-spectra. (PDF)

## Notes

This manuscript was first submitted as a preprint to ChemRxiv on December 17, 2024 (https://chemrxiv.org/engage/chemrxiv/article-details/675c0fbb085116a13346df8a).
