# Supplementary material for: Cage Alkyl Carbenes Provide Experimental Evidence for Isotope-Controlled Selectivity in Competing Tunneling Reactions
Source: J Am Chem Soc. 2025 May 12;147(20):16717–21. doi: 10.1021/jacs.4c18129 (PMC12100647; doi:10.1021/jacs.4c18129)
Supplement: Supplementary file 1 [file ja4c18129_si_001.pdf]

Supporting Information

Cage Alkyl Carbenes Provide Experimental Evidence for Isotope-Controlled Selectivity in Competing Tunneling Reactions

Akkad Danho,[a] Bastian Bernhardt,[a] Dennis Gerbig,[a] Marija Alešković,[b] and Peter R. Schreiner*[a]

[a] Institute of Organic Chemistry, Justus Liebig University, Heinrich-Buff-Ring 17, 35392 Giessen, Germany

[b] Division of Organic Chemistry and Biochemistry, Ruđer Bošković Institute, Bijenička cesta 54, 10000 Zagreb, Croatia

General Information 2

Synthesis 3

IR Spectral Data 18

UV/Vis Spectral Data 68

Computed Potential Energy Surfaces 74

Tunneling Computations 77

Cartesian Coordinates of Computed Geometries 78

References 111

Full Citations for Electronic Structure Codes 112

General Information

All chemicals were purchased from Sigma Aldrich, Carl Roth, Acros Organics, or TCI in the highest purity grade possible and used without further purification. All solvents were distilled prior to use. If necessary, reactions were carried out under argon atmosphere using standard Schlenk techniques. Dry solvents were ordered from Acros Organics (H_2O <50 ppm). Analytical thin-layer chromatography was performed on plastic-backed silica gel 60 plates coated with a fluorescence indicator by Macherey Nagel. Visualization was performed by UV light (254 nm) and/or ceric ammonium molybdate (CAM) stain*. Reaction control was performed via TLC-MS by an Advion expression® CMS or GC-MS analysis (Agilent 7820 GC with 5977B MSD). *180 mL H_2O, 5 g ammonium molybdate tetrahydrate, 2 g cerium ammonium sulfate dihydrate, 20 mL conc. H_2SO_4. High resolution masses were obtained on a Bruker MicrOTof or Bruker Impact II. Nuclear Magnetic Resonance Spectra were recorded on a Bruker AV 400, AV 400 HD, or AV 600 spectrometer at 298 K. Chemical shifts in ppm are reported relative to the residual $CDCl_3$ signal (7.26 ppm/77.16 ppm). Signal multiplicities are reported as follows: s – singlet, br s – broad singlet, d – doublet, t – triplet, q – quartet, hept – septet, m – multiplet or combinations thereof. All spectral data are reported based on appearance.

Matrix Apparatus Design. A Sumitomo cryostat system consisting of an RDK 408D2 closed-cycle refrigerator cold head and an F-70 compressor unit was used for matrix isolation experiments. A polished CsI window was mounted in the cold head sample holder. The sample holder, connected with silicon diodes for temperature measurements, was covered by a vacuum shroud, which was equipped with KBr windows to allow for IR measurements. In some experiments BaF_2 windows were used due to their higher transparency when measuring UV/vis spectra. The sample and the host gas (Ar, purity of 99.999%) were co-deposited at 3.5 K. All spectral data were collected at 3.5 K. The pyrolysis zone was equipped with a heatable 90 mm long quartz tube (inner diameter 7 mm), controlled by a Ni/CrNi thermocouple. The travel distance of the sample from the pyrolysis zone to the matrix was ~45 mm. Ar was stored in a 2 L gas balloon, which was evacuated and filled three times before every experiment. The samples were evaporated from a Schlenk tube at –20 °C (EtOH and dry ice) and reduced pressure (~3×10^{-6} mbar) and co-deposited with a high excess of argon for **5**, **6**, and **11** and nitrogen for **13** und d_2-**13** on both sides of the matrix window in the dark (preventing unwanted photochemistry) at a rate of ~1 mbar min^{-1}, based on the pressure inside the Ar/N_2 balloon. Pyrolyses were carried out at 800 °C. IR spectra were recorded between 7000 and 350 cm^{-1} with a resolution of 0.7 cm^{-1} on Bruker Vertex 70 FTIR spectrometer. A spectrum of the cold matrix window before deposition was used as background spectrum for the subsequent IR measurements. Kinetic IR measurements were taken every half h for 48 h. UV/vis spectra were recorded between 190 and 800 nm with a resolution of 1 nm with a Jasco V-760 spectrophotometer. A high-pressure-mercury lamp equipped with a monochromator (LOT Quantum Design) or a low-pressure-mercury lamp (Gräntzel) fitted with a Vycor filter were used for irradiation of the matrix during photochemical experiments.

Computations. All DFT computations were performed with the Gaussian16, Revision C.01 (full citations for electronic structure codes are given at the end of this document) at the B3LYP/6-311++G(3df,2pd) and B3LYP/def2-TZVPP[1-2] level of theory. The keywords Opt and Freq=NoRaman were used for the characterization of minima on the PES. For transition structures the keyword Opt=(ts,tight,calcfc,noeigen) was used. UV/Vis absorptions were computed by using the keyword td(50-50,nstates=10). The reaction rates were computed using the multidimensional, small-curvature tunneling (SCT) method,[3] implemented in the program *Polyrate*.

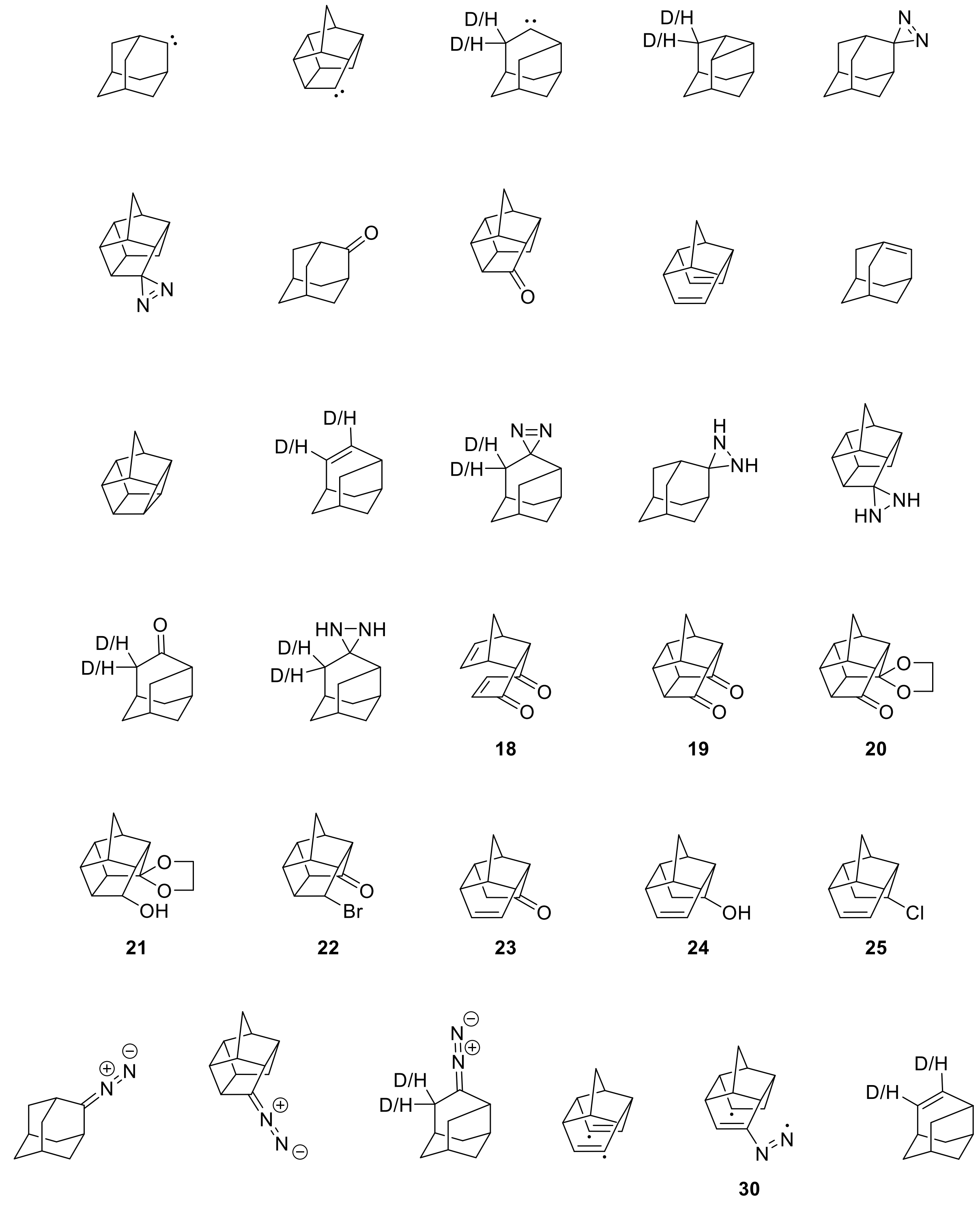

Figure S1: Structures under consideration in this study.

Synthesis

Adamantane diaziridine[4] (14)

To a solution of 0.300 g (1.99 mmol, 1.00 equiv) of adamantanone in 5 mL of MeOH at –78 °C, 20 mL of NH_3 was added via a cold finger cooled to –40 °C (using ethanol and dry ice). The reaction was stirred for 1 h at –78 °C, and then a solution of 0.500 g (4.42 mmol, 2.22 equiv) of hydroxylamine-*O*-sulfonic acid in 5 mL of MeOH was added in two portions. The reaction mixture was stirred at 0 °C for 16 h. Subsequently, 10 mL of H_2O was added, the resulting mixture was extracted three times with 10 mL of CH_2Cl_2. The combined organic layers were dried with $MgSO_4$, filtered, and the solvent was removed under reduced pressure, yielding a colorless solid. Due to product lability, it was immediately utilized for the subsequent step.

Adamantane diazirine (5)

To a solution of 0.328 g of crude **14** in 10 mL of MeOH at room temperature, 0.671 g (3.95 mmol) of $AgNO_3$ dissolved in 2 mL of water and 0.160 g (4.00 mmol) of NaOH dissolved in 2 mL of H_2O were added. The reaction mixture was stirred for 30 min, the inorganic precipitate was filtered off, and then washed with MeOH and H_2O. The filtrate was then extracted two times with 10 mL of CH_2Cl_2. The combined organic layers were dried with $MgSO_4$, filtered, and the solvent was removed under reduced pressure. Purification by flash column chromatography (using *n*-hexane) yielded 0.098 g (0.604 mmol) of adamantane diazirine as a colorless solid in 30% yield in two steps.

The obtained spectra are consistent with the literature.[4]

^{1}H NMR (300 MHz, $CDCl_3$): δ/ppm = 1.99-2.14 (m, 6H), 1.73-1.86 (m, 6H), 0.65 (s, 2H)

^{13}C NMR (75 MHz, $CDCl_3$): δ/ppm = 37.06, 35.39, 34.73, 27.81

Pentacycloundecane diaziridine[4] (15)

To a solution of 0.320 g (1.99 mmol, 1.00 equiv) of pentacycloundecanone in 5 mL of MeOH at –78 °C, 20 mL of NH_3 was added via a cold finger cooled to –40 °C (using ethanol and dry ice). The reaction was stirred for 1 h at –78 °C, and then a solution of 0.500 g (4.42 mmol, 2.22 equiv) of hydroxylamine-*O*-sulfonic acid in 5 mL of MeOH was added in two portions. The reaction mixture was stirred at 0 °C for 16 h. Subsequently, 10 mL of H_2O was added, and the resulting mixture was extracted three times with 10 mL of CH_2Cl_2. The combined organic layers were dried over $MgSO_4$, filtered, and the solvent was removed under reduced pressure, yielding a colorless solid. Due to product lability, it was immediately utilized for the subsequent step.

Pentacycloundecane diazirine (6)

To a solution of 0.328 g of crude **15** in 10 mL of MeOH at room temperature, 0.671 g (3.95 mmol) of $AgNO_3$ dissolved in 2 mL of water and 0.160 g (4.00 mmol) of NaOH dissolved in 2 mL of H_2O were added. The reaction mixture was stirred for 30 min, the inorganic precipitate was filtered off, and then washed with MeOH and H_2O. The filtrate was then extracted two times with 10 mL of CH_2Cl_2. The combined organic layers were dried with $MgSO_4$, filtered, and the solvent was removed under reduced

pressure. Purification by flash column chromatography (using *n*-hexane) yielded 0.158 g (0.919 mmol) of pentacycloundecane diazirine as a colorless solid in 46% yield in two steps.

The obtained spectra are consistent with the literature.[4]

^{1}H NMR (600 MHz, $CDCl_3$): δ/ppm = 2.78-2.86 (m, 2H), 2.62-2.64 (m, 2H), 2.49-2.53 (m, 1H), 2.38-2.43 (m, 2H), 1.68 (d, 1H, *J* = 10.6 Hz), 1.47-1.51 (m, 1H), 1.38 (dt, 1H, *J* = 4.1 Hz, *J* = 12.3 Hz), 1.33 (d, 1H, *J* = 10.6 Hz), 1.16-1.20 (m, 1H)

^{13}C NMR (150 MHz, $CDCl_3$): δ/ppm = 47.75, 46.17, 44.20, 43.89, 43.12, 41.40, 39.28, 38.27, 37.44, 35.39, 30.13

Protoadamantanone (16)

Under Schlenk conditions, 58.3 g (0.132 mol, 2.03 equiv) of lead tetraacetate, 37.4 g (0.147 mol, 2.26 equiv) of iodine, and 10.0 g (0.065 mol, 1.00 equiv) 1-adamantanol were dissolved in 600 mL of dry benzene. The flask was heated for 20 min at 80 °C and then for 2 h at 72 °C. The mixture was allowed to cool to room temperature for 1 h. The inorganic salts were filtered off and washed with five 50 mL portions of diethyl ether. The combined organic layers were shaken with 500 mL of saturated aqueous sodium bisulfite until the dark red color disappeared. The organic phase was then washed with 500 mL of H_2O and 250 mL of saturated aqueous sodium hydrogen carbonate. Afterwards, the organic phase was dried over $MgSO_4$, filtered, and the solvent was removed under reduced pressure to approximately 20 mL, as *endo*-7-iodomethylbicyclo[3.3.1]nonan-3-one is unstable and was immediately used in the next step.

7-Iodomethylbicyclo[3.3.1]nonan-3-one was dissolved in 150 mL of MeOH, to which 7 g (0.1 mol) of KOH were added, and the reaction mixture was refluxed for 3 h. Afterwards, the reaction mixture was quenched with 300 mL of ice-cold H_2O. The resulting mixture was then extracted five times with 100 mL of diethyl ether. The combined organic layers were dried over $MgSO_4$, filtered, and the solvent was removed under reduced pressure. Purification by flash column chromatography (using *n*-pentane and activity III neutral alumina) yielded 7.0 g of protoadamantanone as a colorless solid in 71% yield.

The obtained spectra are consistent with the literature.[5]

^{1}H NMR (400 MHz, $CDCl_3$): δ/ppm = 2.73 (m, 1H), 2.61 (m, 1H), 2.54 (m, 1H), 2.41 (m, 1H), 2.25 (m, 1H), 1.95 (m, 2H), 1.81 (m, 1H), 1.76-1.50 (m, 6H)

^{13}C NMR (100 MHz, $CDCl_3$): δ/ppm = 216.75, 51.19, 45.09, 41.44, 38.20, 37.44, 37.31, 37.21, 34.89, 29.59

HRMS (ESI): m/z = 153.0940 $[M+Na]^+$, 153.0937 (calcd)

Protoadamantane diaziridine (17)

To a solution of 300 mg (1.99 mmol, 1.00 equiv) of **16** in 5 mL of NH_3 in MeOH (7M) at 0 °C, 300 mg (2.65 mmol, 1.33 equiv) of hydroxylamine-*O*-sulfonic acid in 3 mL of MeOH was added. The reaction mixture was stirred for 4 days at 0 °C. The solid was separated, and the liquid was removed under reduced pressure. To both solids, 20 mL of CH_2Cl_2 was added, and the resulting suspension was filtered. The extract was washed three times with 10 mL of 1M H_2SO_4 cooled to 0 °C. To the aqueous layer, 10 mL of cold (0 °C) 2M NaOH was added. The aqueous layer was then extracted three times with 30 mL of CH_2Cl_2. The combined organic layers were dried over $MgSO_4$, filtered, and the solvent was removed

under reduced pressure, yielding a colorless solid. Due to product lability, it was immediately utilized for the subsequent step.

Protoadamantane diazirine (13)

To a solution of 0.244 g of crude **17** in 10 mL of MeOH at room temperature, 0.671 g (3.95 mmol) of $AgNO_3$ dissolved in 2 mL of water and 0.160 g (4.00 mmol) of NaOH dissolved in 2 mL of H_2O were added. The reaction mixture was stirred for 30 min, the inorganic precipitate was filtered off, and then washed with MeOH and H_2O. The filtrate was then extracted two times with 10 mL of CH_2Cl_2. The combined organic layers were dried over $MgSO_4$, filtered, and the solvent was removed under reduced pressure. Purification by flash column chromatography (using *n*-pentane) yielded 0.016 g (0.099 mmol) of protoadamantane diazirine as a colorless solid in 5% yield over two steps.

^{1}H NMR (400 MHz, $CDCl_3$): δ/ppm = 2.46 (q, *J* = 4.0 Hz, 1H), 2.24 (m, 1H), 2.05 (m, 3H), 1.91-1.76 (m, 3H), 1.61 (m, 1H), 1.54 (m, 3H), 1.00 (t, *J* = 6.1 Hz, 1H), 0.72 (qt, *J* = 2.1Hz, 4Hz, 1H)

^{13}C NMR (100 MHz, $CDCl_3$): δ/ppm = 41.26, 41.16, 38.90, 38.19, 36.22, 35.35, 34.43, 34.41, 28.63

HRMS: m/z = 135.1171 $[M–N_2+H]^+$, 135.1168 (calcd)

Deuteration of protoadamantane (*d*$_2$-16)

A solution of 1.000 g (6.442 mmol, 1 equiv) of protoadamantanone, 3 mL NaOD (in D_2O 40% w/w), and 5 mL of dry dioxane was refluxed for 24 h. After cooling the reaction mixture to rt, dioxane was evaporated and the obtained crude oil reaction mixture was divided between water and CH_2Cl_2. The water layer was extracted 3 times with CH_2Cl_2. The combined organic layers were dried over $MgSO_4$, filtered, and the solvent was removed under reduced pressure. This process was repeated three times and yielded 0.721 g (4.737 mmol, 74%) of *d*$_2$-protoadamantanone.

^{1}H NMR (400 MHz, $CDCl_3$): δ/ppm = 2.73 (m, 1H), 2.61 (m, 1H), 2.41 (m, 1H), 2.22 (m, 1H), 2.00-1.91 (m, 2H), 1.83-1.49 (m, 6H)

^{13}C NMR (100 MHz, $CDCl_3$): δ/ppm = 216.89, 51.18, 41.43, 38.14, 37.40, 37.35, 37.21, 34.82, 29.41

HRMS (ESI): m/z = 175.1063 $[M+Na]^+$, 175.1062 (calcd)

***d*$_2$-Protoadamantane diaziridine (*d*$_2$-17)**

To a solution of 300 mg (1.99 mmol, 1.00 equiv) of *d*$_2$-protoadamantanone in 5 mL of NH_3 in MeOH (7M) at 0 °C, 300 mg (2.65 mmol, 1.33 equiv) of hydroxylamine-*O*-sulfonic acid in 3 mL of MeOH was added. The reaction mixture was stirred for 4 days at 0 °C. The solid was separated, and the liquid was removed under reduced pressure. To both solids, 20 mL of CH_2Cl_2 was added, and the resulting suspension was filtered. The extract was washed three times with 10 mL of 1M H_2SO_4 cooled to 0 °C. To the aqueous layer, 10 mL of cold (0 °C) 2M NaOH was added. The aqueous layer was then extracted three times with 30 mL of CH_2Cl_2. The combined organic layers were dried over $MgSO_4$, filtered, and the solvent was removed under reduced pressure, yielding a colorless solid. Due to product lability, it was immediately utilized for the subsequent step.

***d_2*-Protoadamantane diazirine (d_2-13)**

To a solution of 0.244 g of crude d_2-**15** in 10 mL of MeOH at room temperature, 0.671 g (3.95 mmol) of $AgNO_3$ dissolved in 2 mL of water and 0.160 g (4.00 mmol) of NaOH dissolved in 2 mL of water were added. The reaction mixture was stirred for 30 min, the inorganic precipitate was filtered off, and then washed with MeOH and H_2O. The filtrate was then extracted two times with 10 mL of CH_2Cl_2. The combined organic layers were dried over $MgSO_4$, filtered, and the solvent was removed under reduced pressure. Purification by flash column chromatography (using *n*-pentane) yielded 0.018 g (0.099 mmol) of ***d_2*-11** as a colorless solid in 5% yield in two steps.

^{1}H NMR (400 MHz, $CDCl_3$): δ/ppm = 2.46 (q, J = 4.0 Hz, 1H), 2.24 (m, 1H), 2.08-2.00 (m, 3 H), 1.90 (m, 1H), 1.82 (m, 1H), 1.61 (m, 1H), 1.55-1.50 (m, 3H), 1.00 (m, 1H)

^{13}C NMR (100 MHz, $CDCl_3$): δ/ppm = 41.43, 41.28, 39.00, 36.41, 35.52, 34.57, 34.55, 28.58

MS (ESI): m/z = 137.1296 $[M–N_2+H]^+$, 137.1294 (calcd)

1,4,4a,8a-Tetrahydro-1,4-methanonaphthalene-5,8-dione (**18**)

28.749 g (267.69 mmol, 1.1 equiv) of freshly cracked cyclopentadiene was added to 17.771 g (248.29 mmol, 1.0 equiv) of purified benzoquinone in 125 mL of ethyl acetate and stirred in an ice bath for 30 min. After adding another 120 mL of ethyl acetate, the reaction mixture was stirred for 30 min at room temperature. Removing the solvent under reduced pressure yielded 38.91 g (223.4 mmol, 90%) of a brown solid.

The obtained spectra are consistent with the literature.[6]

Pentacyclo[5.4.0.0^{2,6}.0^{3,10}.0^{5,6}]undecane-8,11-dione (19)

38.91 g (223.4 mmol, 1.0 equiv) of **18** was dissolved in 100 mL of ethyl acetate and irradiated in the photoreactor for 20 h. The solvent was removed under reduced pressure. The crude product was recrystallized in ethyl acetate and yielded 10.60 g (57.42 mmol, 25%) of a yellow solid.

The obtained spectra are consistent with the literature.[7]

Pentacyclo[5.4.0.0^{2,6}.0^{3,10}.0^{5,9}]undecane-8,11-dione monoethylene ketal (20)

23.524 g (135.04 mmol, 1.00 equiv) of **19**, 8.421 g (135.7 mmol, 1.01 equiv), and 0.301 g (1.74 mmol, 0.01 equiv) of *p*-toluenesulfonic acid were dissolved in 110 mL of benzene. The mixture was stirred under reflux at 85 °C for 5 h. The mixture was then poured onto 50 mL of ice-cold 10% aqueous Na_2CO_3. The organic phase was extracted with CH_2Cl_2. The combined organic layers were dried over $MgSO_4$, filtered, and the solvent was removed under reduced pressure. Purification by flash column chromatography (using CH_2Cl_2) yielded 17.211 g (77.89 mmol, 58%) of a yellow solid.

The obtained spectra are consistent with the literature.[8]

Spiro[1,3-dioxolane-2,5′(1′a*H*)-[1,2,4]ethanylylidene[1*H*]cyclobuta[*cd*]pentalen]-7′-ol, hexahydro-(9CI) (21)

2.205 g (10.10 mmol, 1.0 equiv) of **20** was dissolved in 25 mL of ethanol, and 0.772 g (20.41 mmol, 2.0 equiv) of $NaBH_4$ was added portion wise, while the solution was stirred at 0 °C. After 2 h, the ice bath was removed, and the solution was stirred for 2 h at room temperature. Then, 17 mL of H_2O, 10 mL of 1N HCl, and 20 mL of CH_2Cl_2 were added. The organic phase was separated, and the aqueous phase was extracted twice with 20 mL of CH_2Cl_2. The combined organic layers were dried over $MgSO_4$, filtered, and the solvent was removed under reduced pressure, yielding 2.144 g (9.73 mmol, 96%) of a yellow liquid.

The obtained spectra are consistent with the literature.[9]

***exo*-11-Bromopentacyclo [5.4.0.0^{2,6}.0^{3,10}.0^{5,9}]undecane-8-one (22)**

2.284 g (10.37 mmol) of **21** was dissolved in 30 mL of 47% HBr. The solution was stirred for 3 h at 80 °C. After cooling to room temperature, the solution was quenched with 100 mL of ice-cold H_2O. The precipitate was washed with water. The organic layer was dried over $MgSO_4$, filtered, and the solvent was removed under reduced pressure, yielding 1.747 g (7.31 mmol, 71%) of a grey solid.

The obtained spectra are consistent with the literature.[9]

Tetracyclo[6.3.0.0^{4,11}.0^{5,9}]undec-2-en-6-one (23)

14.148 g (59.17 mmol, 1.0 equiv) of **22** and 22.50 g (344.1 mmol, 5.8 equiv) of zinc powder were added to 200 mL of glacial acetic acid. The suspension was stirred at 125 °C for 8 h, and after cooling to room temperature, the mixture was filtered, and the solid was washed with diethyl ether. The organic phase was washed with water and twice with aqueous $NaHCO_3$. The organic layer was dried over $MgSO_4$, filtered, and the solvent was removed under reduced pressure, yielding 6.507 g (40.62 mmol, 69%) of a colorless solid.

The obtained spectra are consistent with the literature.[9]

Tetracyclo[6.3.0.0^{4,11}.0^{5,9}]undec-2-en-6-ol (24)

To a solution of 1.744 g (45.96 mmol, 1.1 equiv) of $LiAlH_4$ in 46 mL of diethyl ether in an ice bath, 6.507 g (40.62 mmol, 1.0 equiv) of **23** in 46 mL of diethyl ether was added. The mixture was stirred for 3 h at 0 °C. Then, the mixture was stirred for 17 h at room temperature. The reaction mixture was quenched with $NaOH_{(aq)}$. After filtering the mixture, the solid was washed with diethyl ether and H_2O. The organic phase was washed twice with H_2O. The organic layer was dried over $MgSO_4$, filtered, and the solvent was removed under reduced pressure, yielding 5.986 g (36.90 mmol, 91%) of a colorless solid.

^{1}H NMR (400 MHz, $CDCl_3$): δ/ppm = 6.39 (q, *J* = 2.1 Hz, 1H), 6.03 (q, *J* = 2.0 Hz, 1H), 4.45 (m, 1H), 2.91 (s, 1H), 2.58-2.51 (m, 1H), 2.50-2.42 (m, 2H), 2.32-2.19 (m, 2H), 1.79-1.72 (m, 1H), 1.60-1.52 (m, 2 H) 1.48 (dd, *J* = 4.1 Hz , *J* = 4.1 Hz, 1H)

^{13}C NMR (100 MHz, $CDCl_3$): δ/ppm = 140.09, 139.56, 59.59, 52.57, 51.78, 47.93, 46.89, 42.55, 39.20, 32.51

Tetracyclo[6.3.0.0^{4,11}.0^{5,9}]undec-2-en-6-chloride (25)

To a solution at 0°C of 5.986 g (36.90 mmol, 1.0 equiv) of **24** and 50 mL of pyridine, 17 mL (27.97 g, 182.4 mmol, 4.9 equiv) of PCl_3 were added. After stirring at 100 °C for 5 h, the mixture was poured into 200 mL of iced water. The organic phase was separated, and the aqueous phase was extracted four times with *n*-pentane. The combined organic phase was washed with 100 mL of 10% $HCl_{(aq)}$ and 10% $NaHCO_{3(aq)}$, followed by H_2O and $NaCl_{(aq)}$. The organic layer was dried over $MgSO_4$, filtered, and the solvent was removed under reduced pressure, yielding 4.504 g (24.93 mmol, 68%) of a yellow oil.

^{1}H NMR (400 MHz, $CDCl_3$): δ/ppm = 5.90 (m, 4H), 3.17 (m, 2H), 2.33 (m, 4H), 1.64 (m, 2H)

^{13}C NMR (100 MHz, $CDCl_3$): δ/ppm = 137.27, 65.07, 49.13, 31.85

Homohypostrophene (9)

To a solution of 4.504 g (24.93 mmol, 1.0 equiv) of **25** in 20 mL of DMSO was added 5.105 g (45.50 mmol) of KO^{t}Bu in 80 mL of DMSO. The solution was stirred for 10 h at 100 °C. H_2O was added, and the mixture was extracted four times with *n*-pentane. The *n*-pentane extract was filtered through a neutral alumina column. The organic layer was dried over $MgSO_4$, filtered, and the solvent was removed under reduced pressure, yielding 1.99 g (13.80 mmol, 55%) of a colorless solid.

The obtained spectra are consistent with the literature.[9]

Homopentaprismane (11)

A solution of 1.013 g (7.02 mmol) **9** in 15 mL benzene were irradiated for 22 h in a photoreactor. Removing the solvent under reduced pressure, yields 0.786 g (5.45 mmol, 78%) of a colorless solid.

The obtained spectra are consistent with the literature.[9]

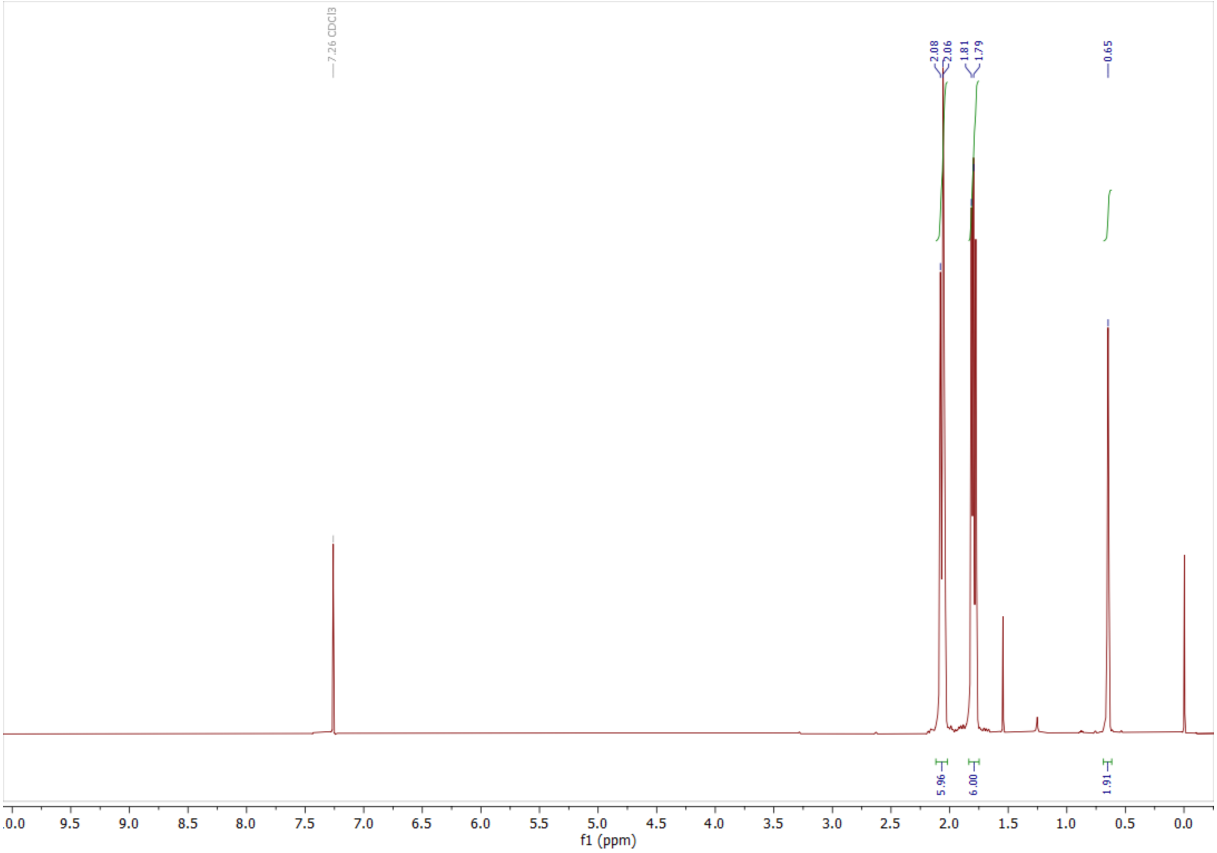

Figure S2: ^{1}H NMR of **Adamantane diazirine (5)**

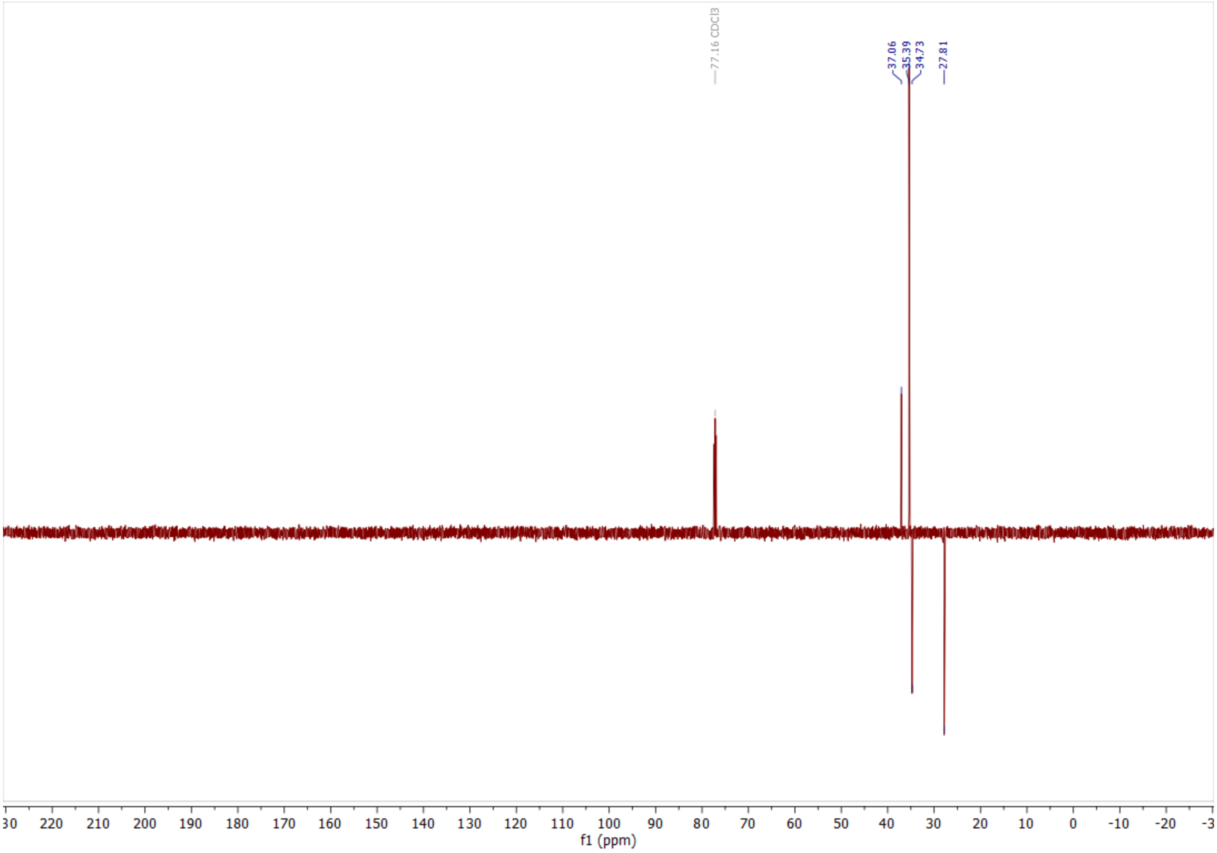

Figure S3: ^{13}C NMR (DEPT-135) of **Adamantane diazirine (5)**

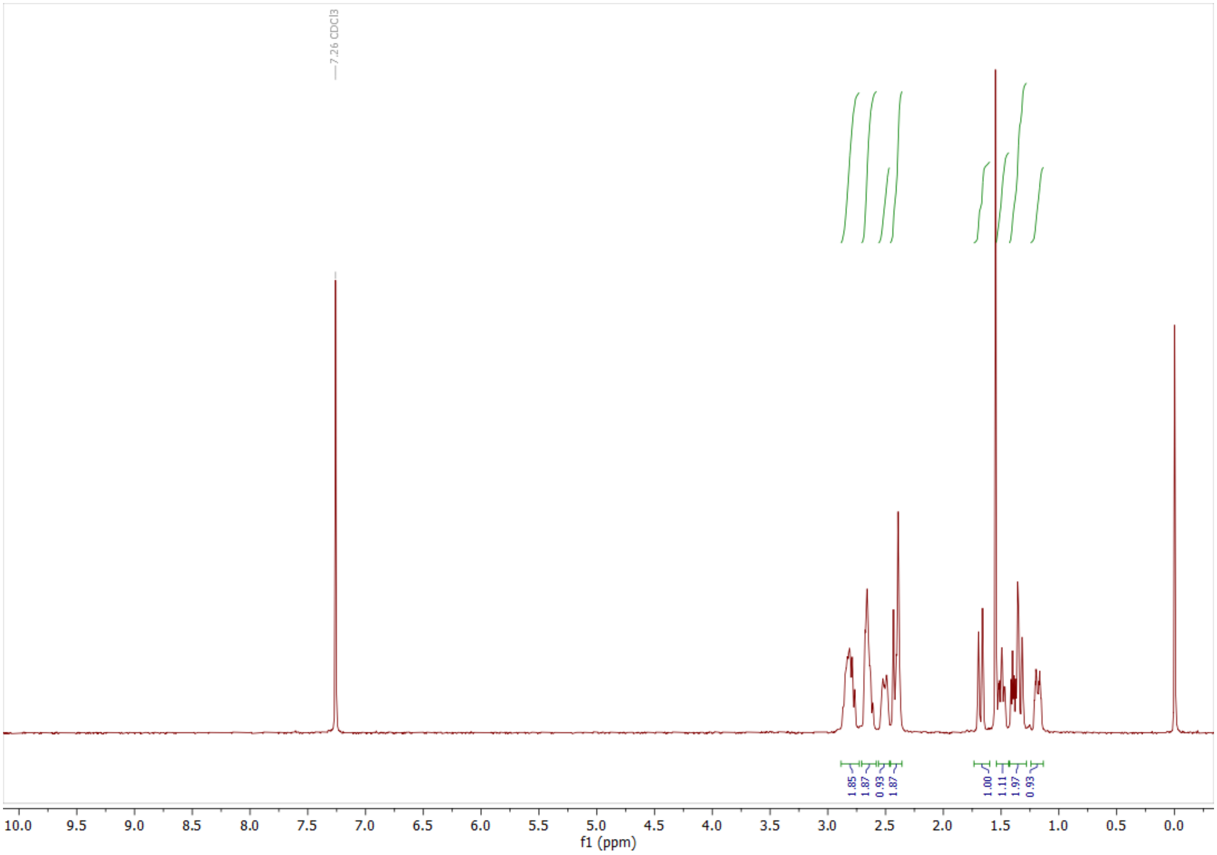

Figure S4: ^{1}H NMR of **Pentacycloundecane diazirine (6)**

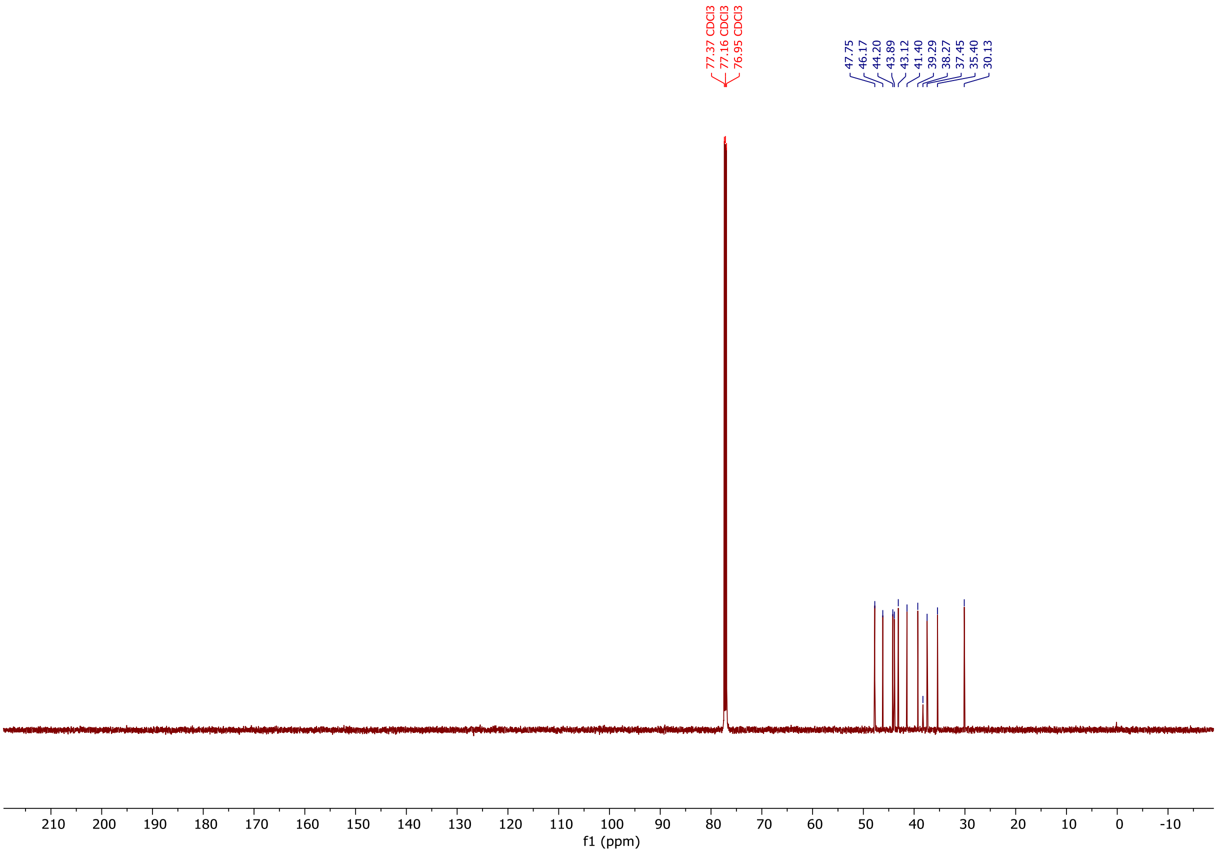

Figure S5: ^{13}C NMR of **Pentacycloundecane diazirine (6)**

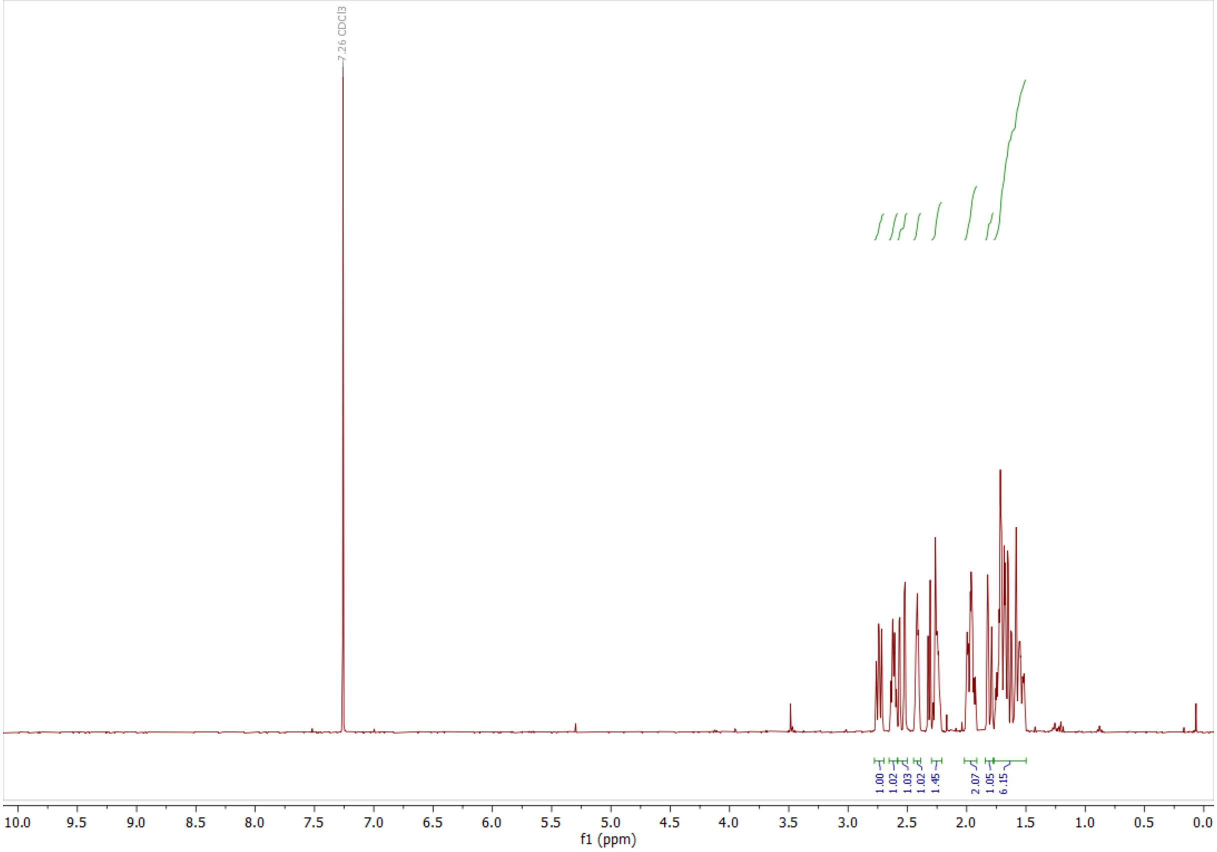

Figure S6: ^{1}H NMR of **Protoadamantanone (16)**

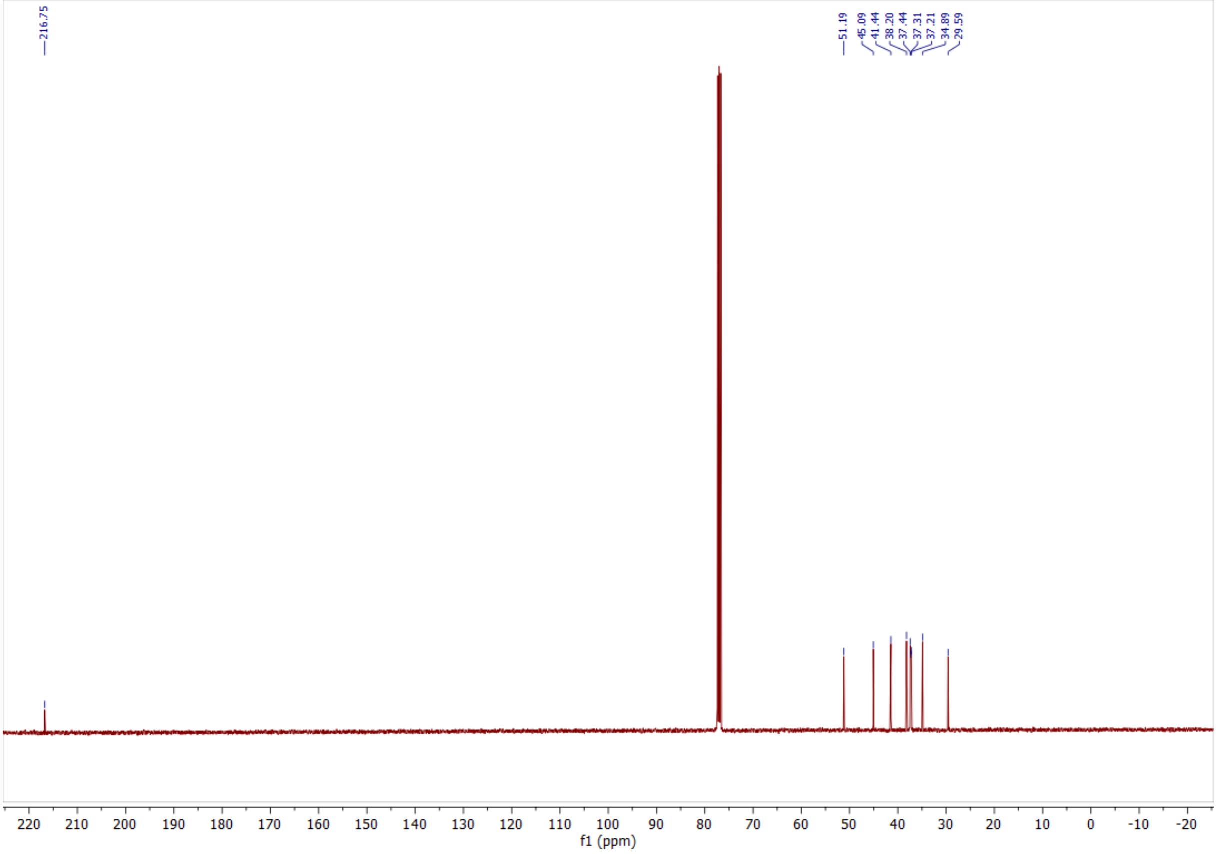

Figure S7: ^{13}C NMR of **Protoadamantanone (16)**

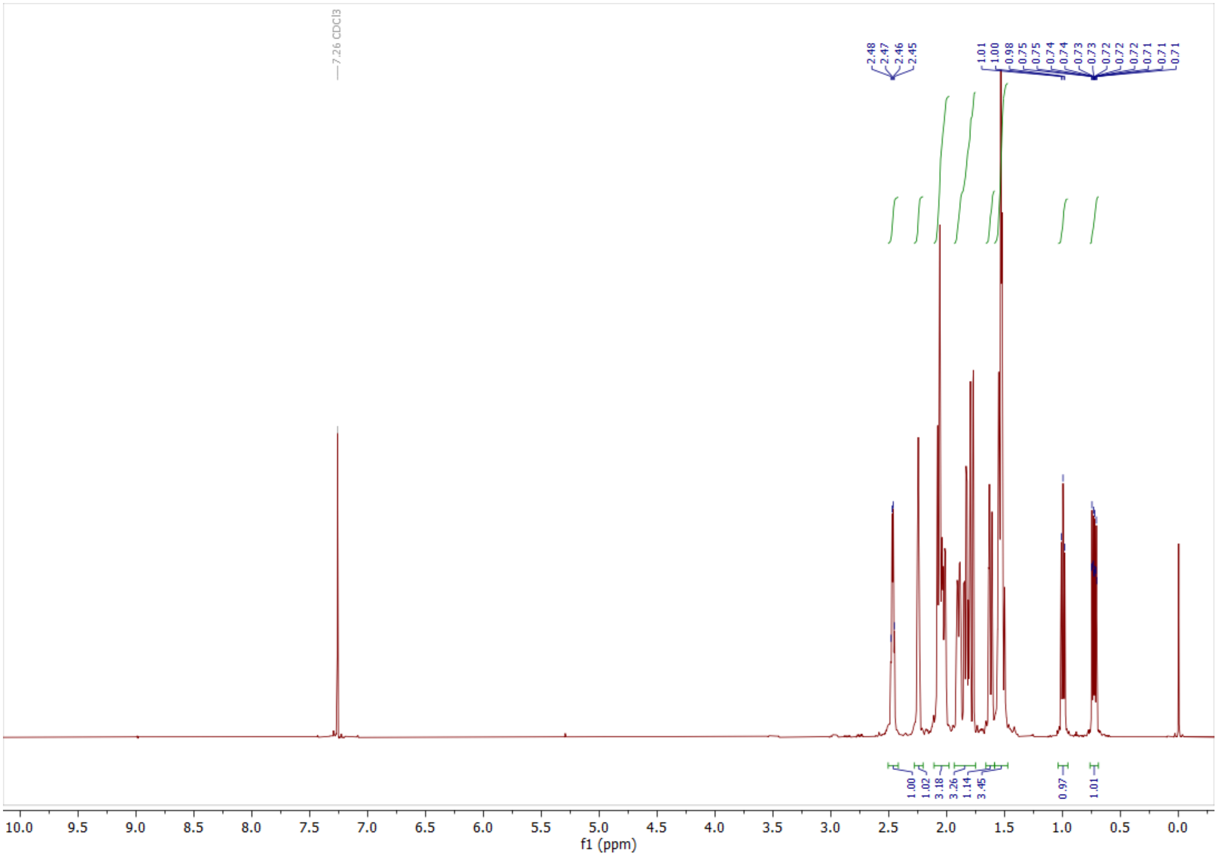

Figure S8: ^{1}H NMR of **Protoadamantane diazirine (13)**

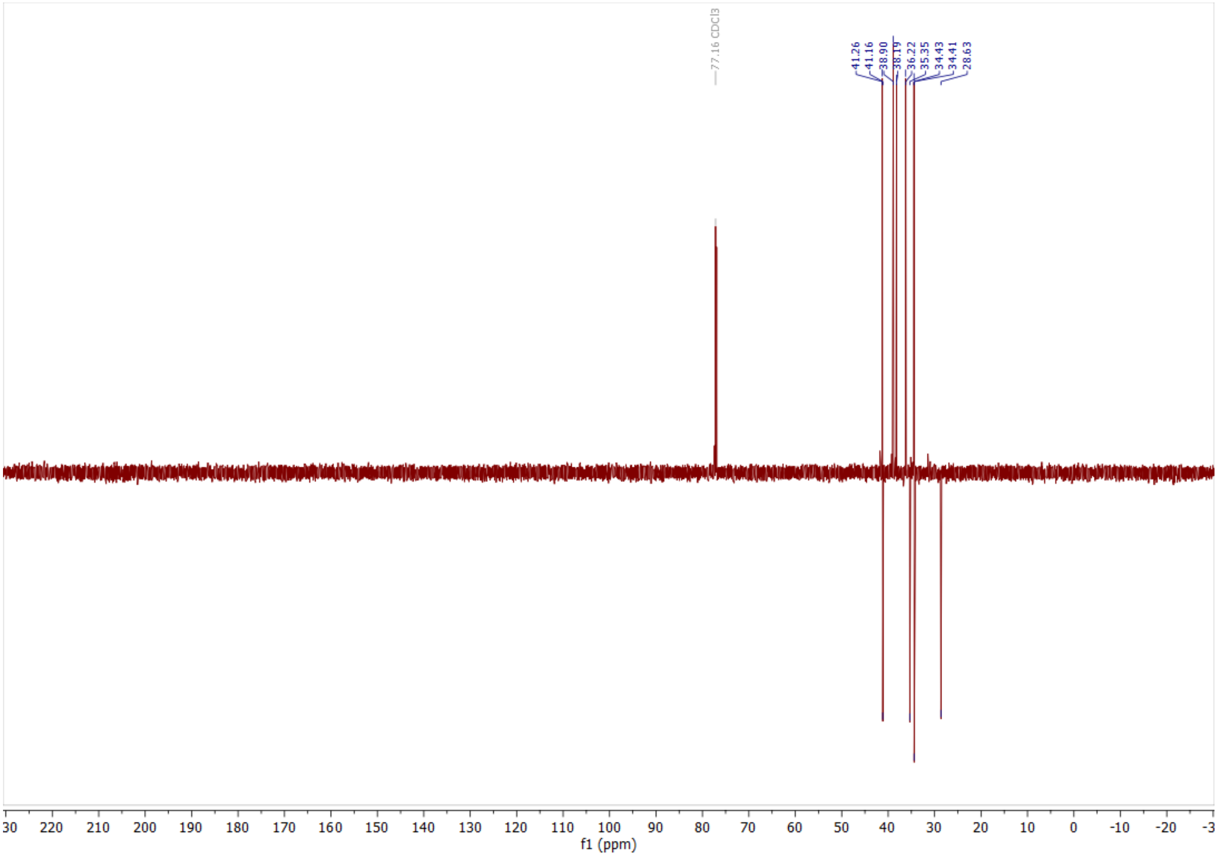

Figure S9: ^{13}C NMR (DEPT-135) of **Protoadamantane diazirine (13)**

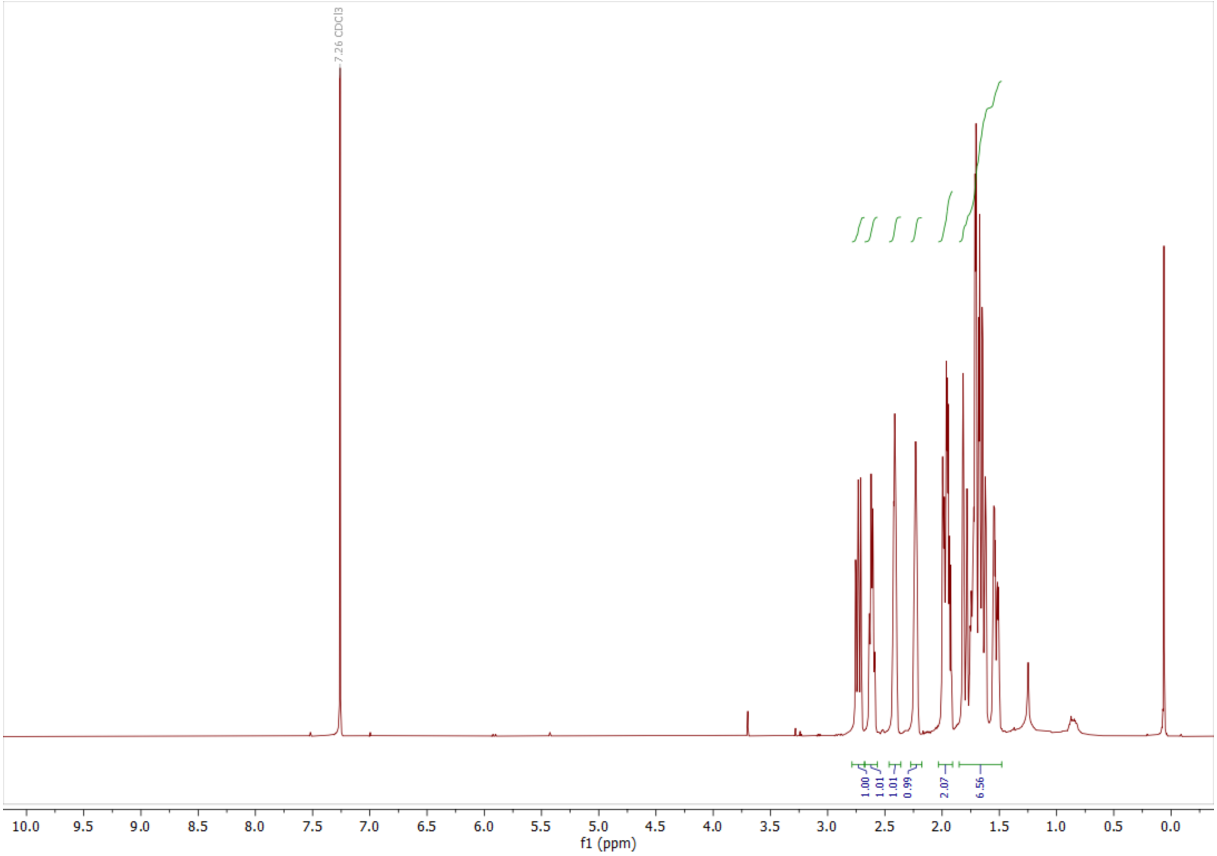

Figure S10: ^{1}H NMR of d_2-**Protoadamantanone (**d_2**-16)**

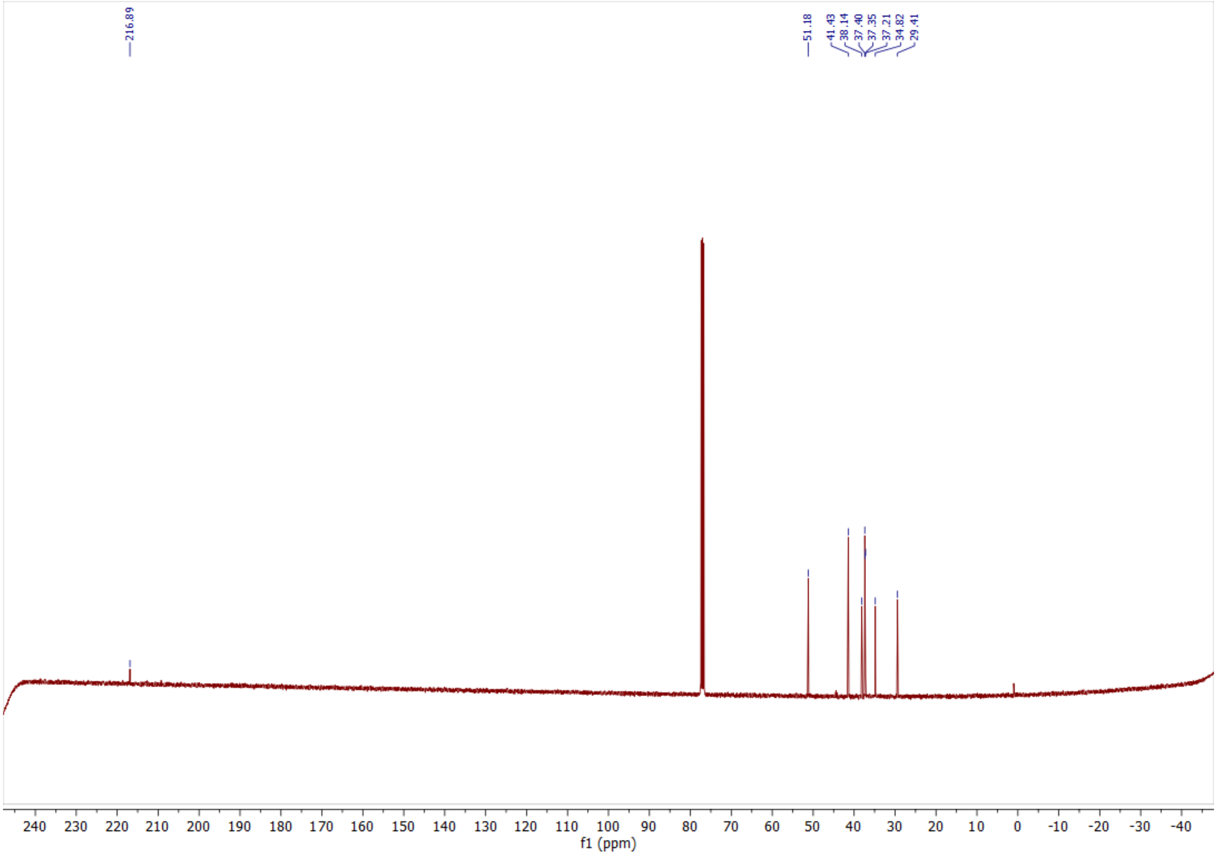

Figure S11: ^{13}C NMR of d_2-**Protoadamantanone (**d_2**-16)**

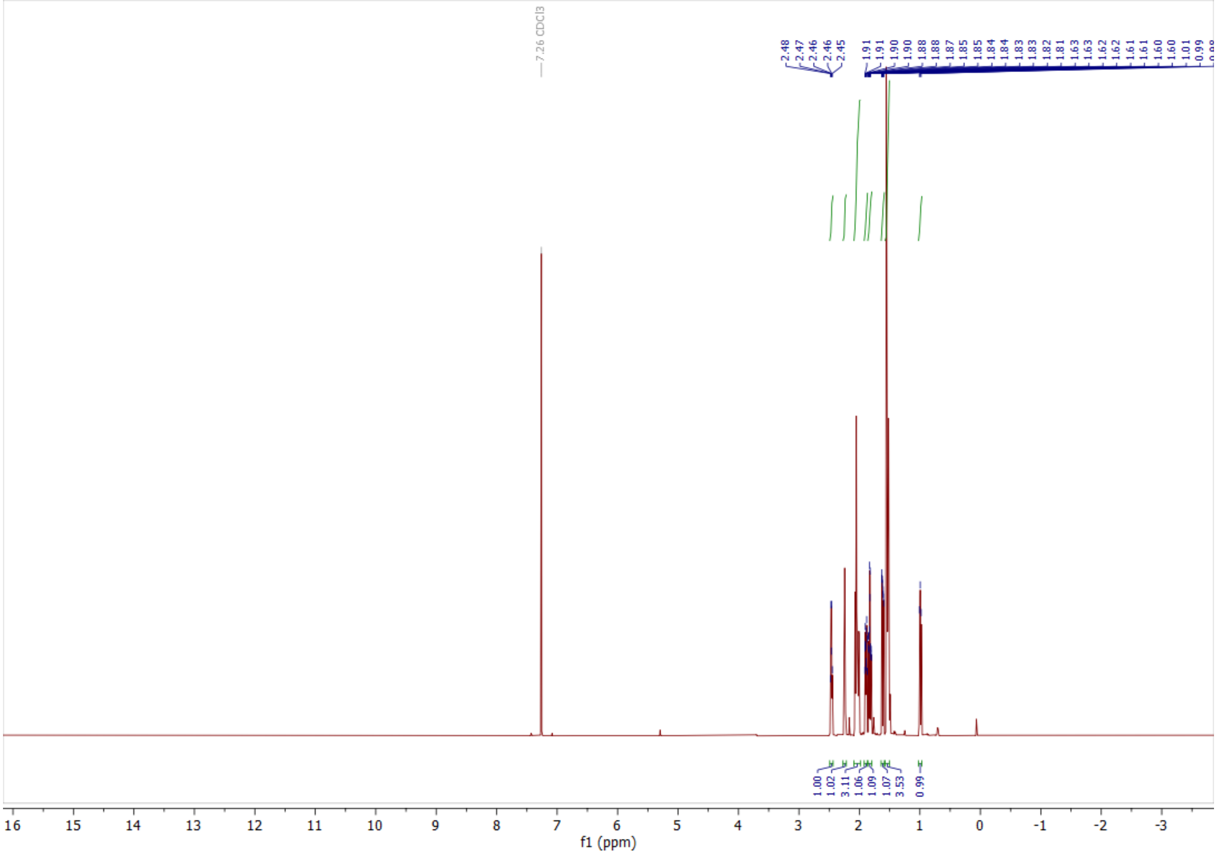

Figure S12: ^{1}H NMR of d_2-**Protoadamantane diazirine (**d_2-**13)**

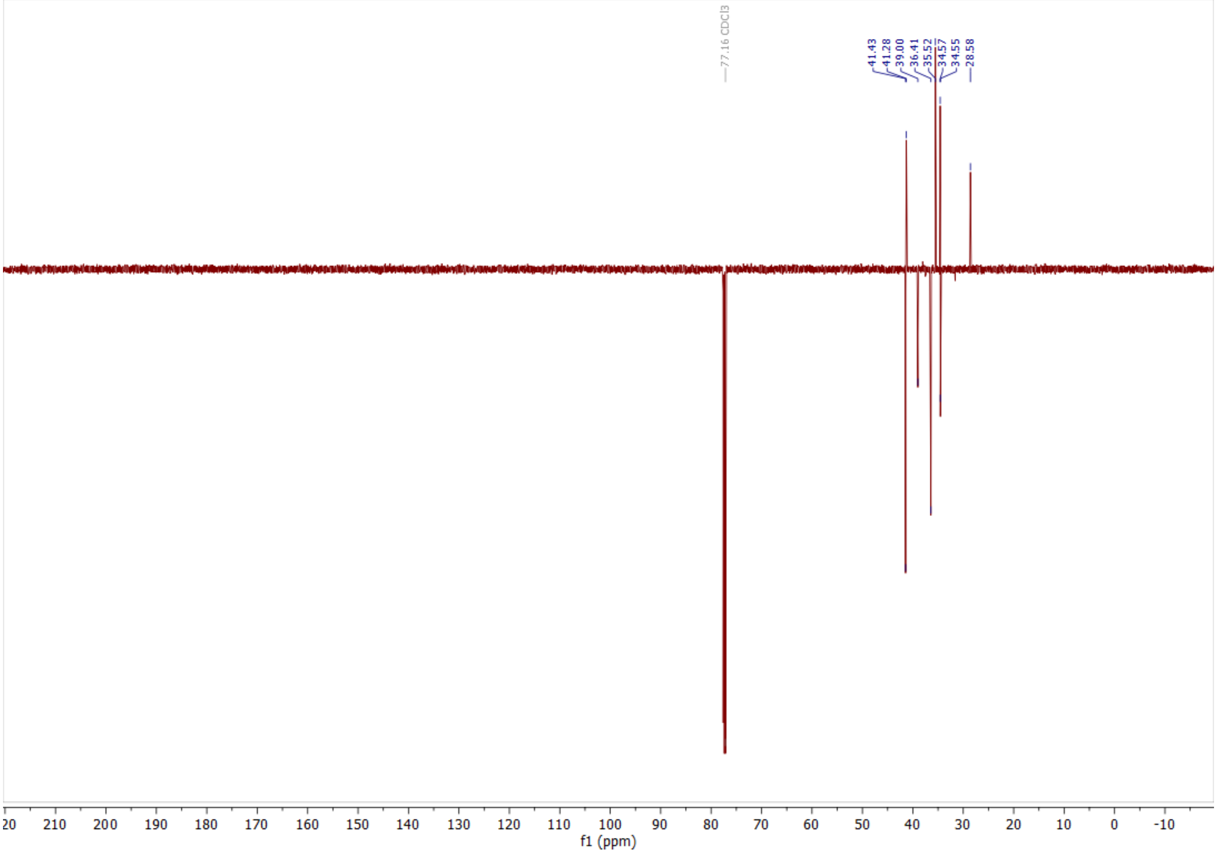

Figure S13: ^{13}C NMR (DEPT-135) of d_2-**Protoadamantane diazirine (**d_2-**13)**

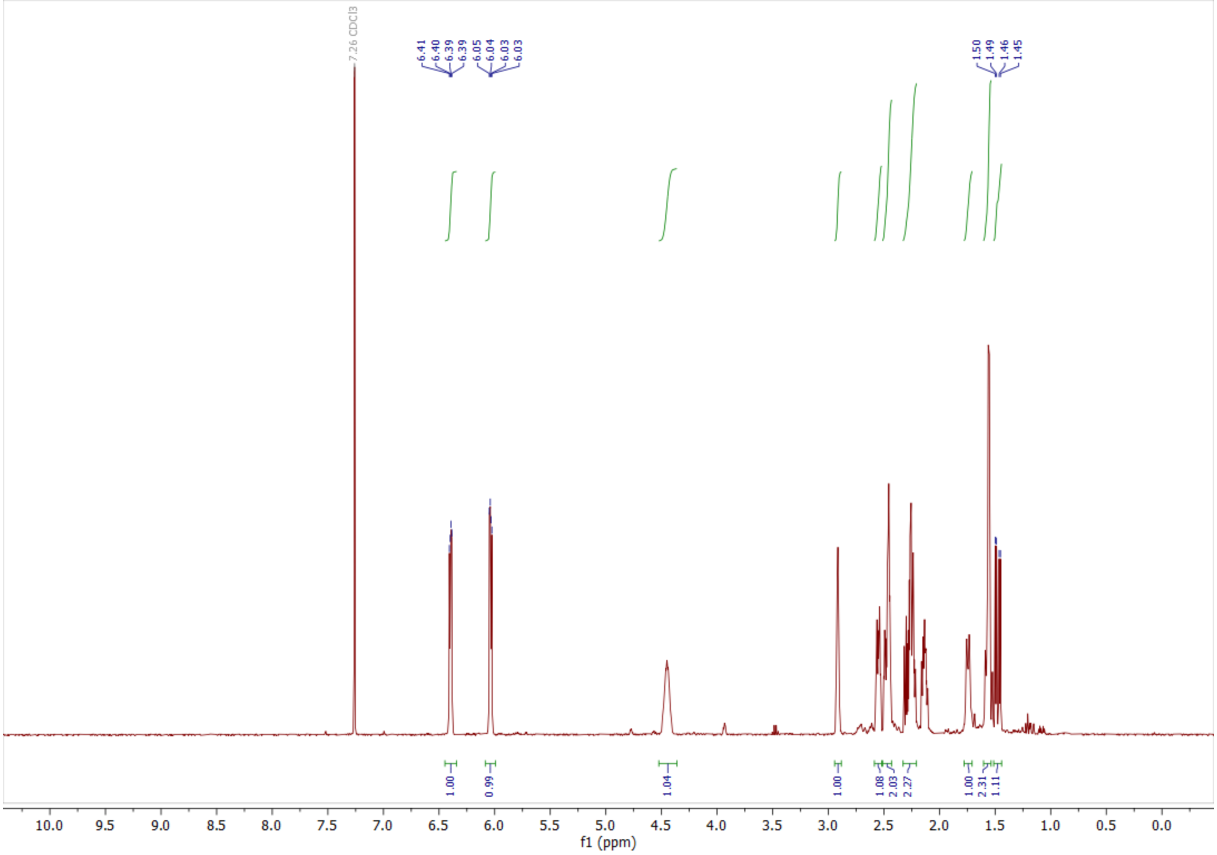

Figure S14: ^{1}H NMR of **Tetracyclo[6.3.0.0^{4,11}.0^{5,9}]undec-2-en-6-ol (24)**

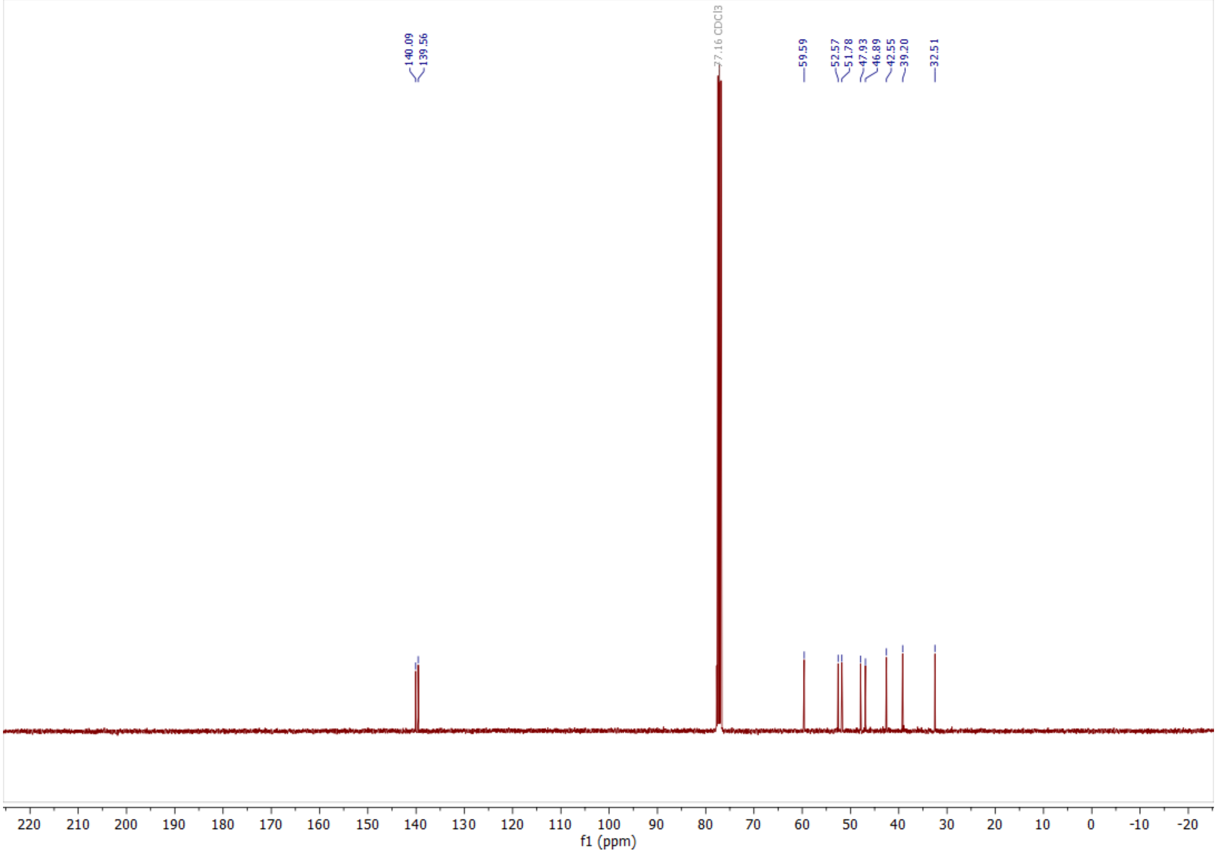

Figure S15: ^{13}C NMR of **Tetracyclo[6.3.0.0^{4,11}.0^{5,9}]undec-2-en-6-ol (24)**

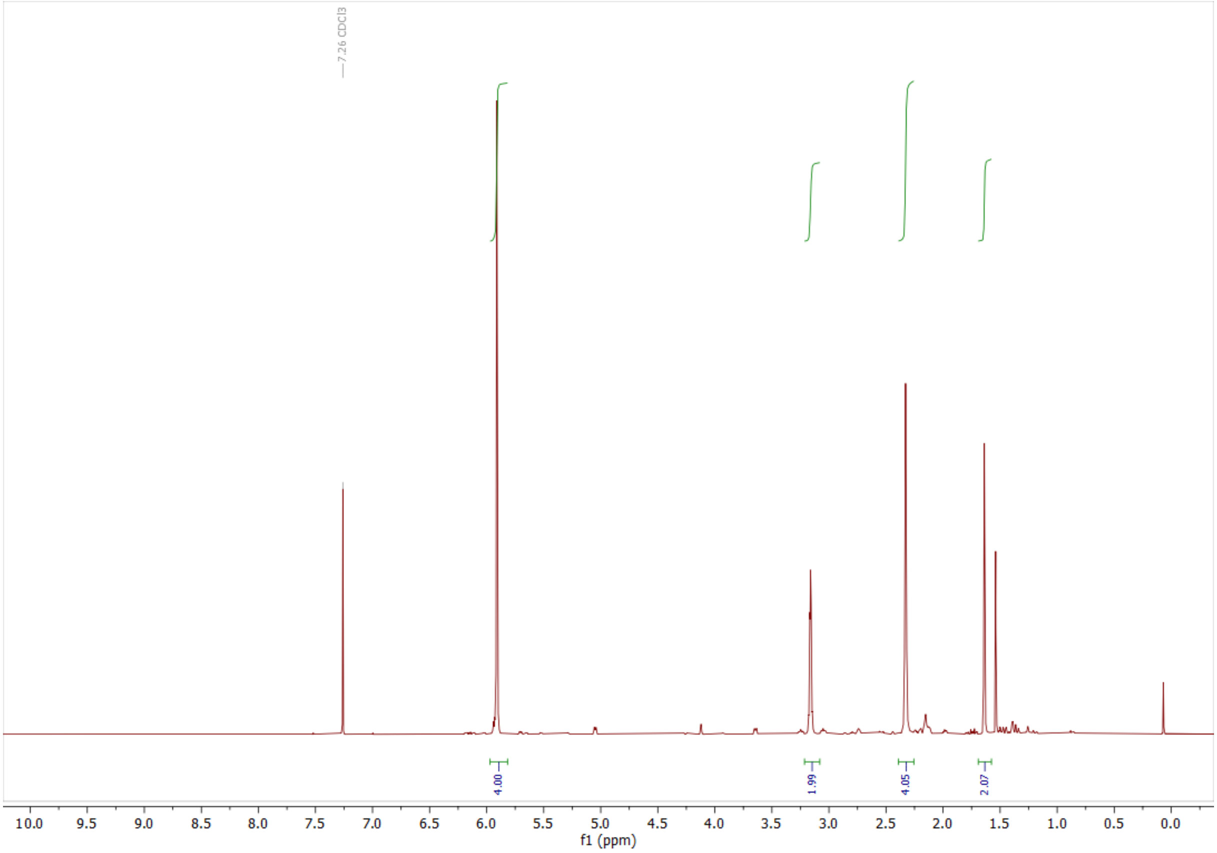

Figure S16: ^{1}H NMR of **Tetracyclo[6.3.0.0^{4,11}.0^{5,9}]undec-2-en-6-chloride (25)**

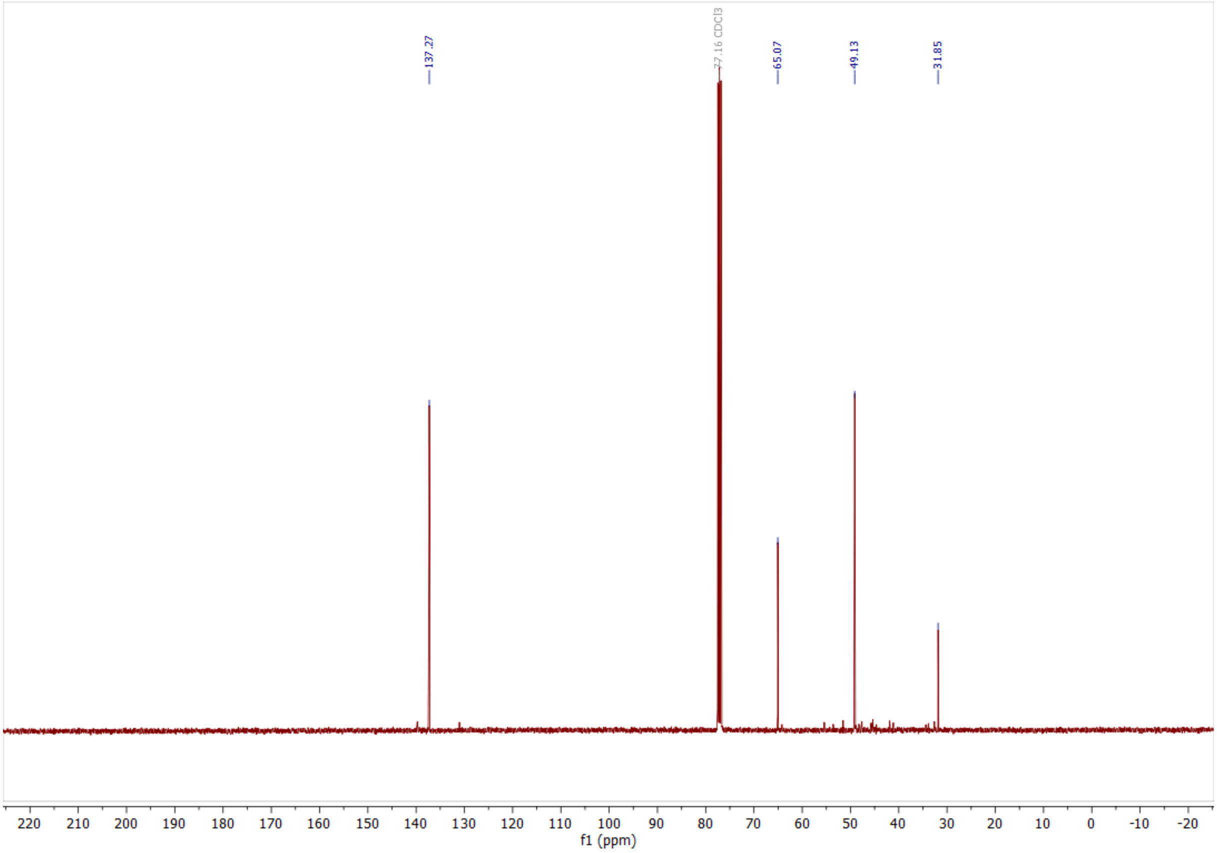

Figure S17: ^{13}C NMR of **Tetracyclo[6.3.0.0^{4,11}.0^{5,9}]undec-2-en-6-chloride (25)**

IR Spectral Data

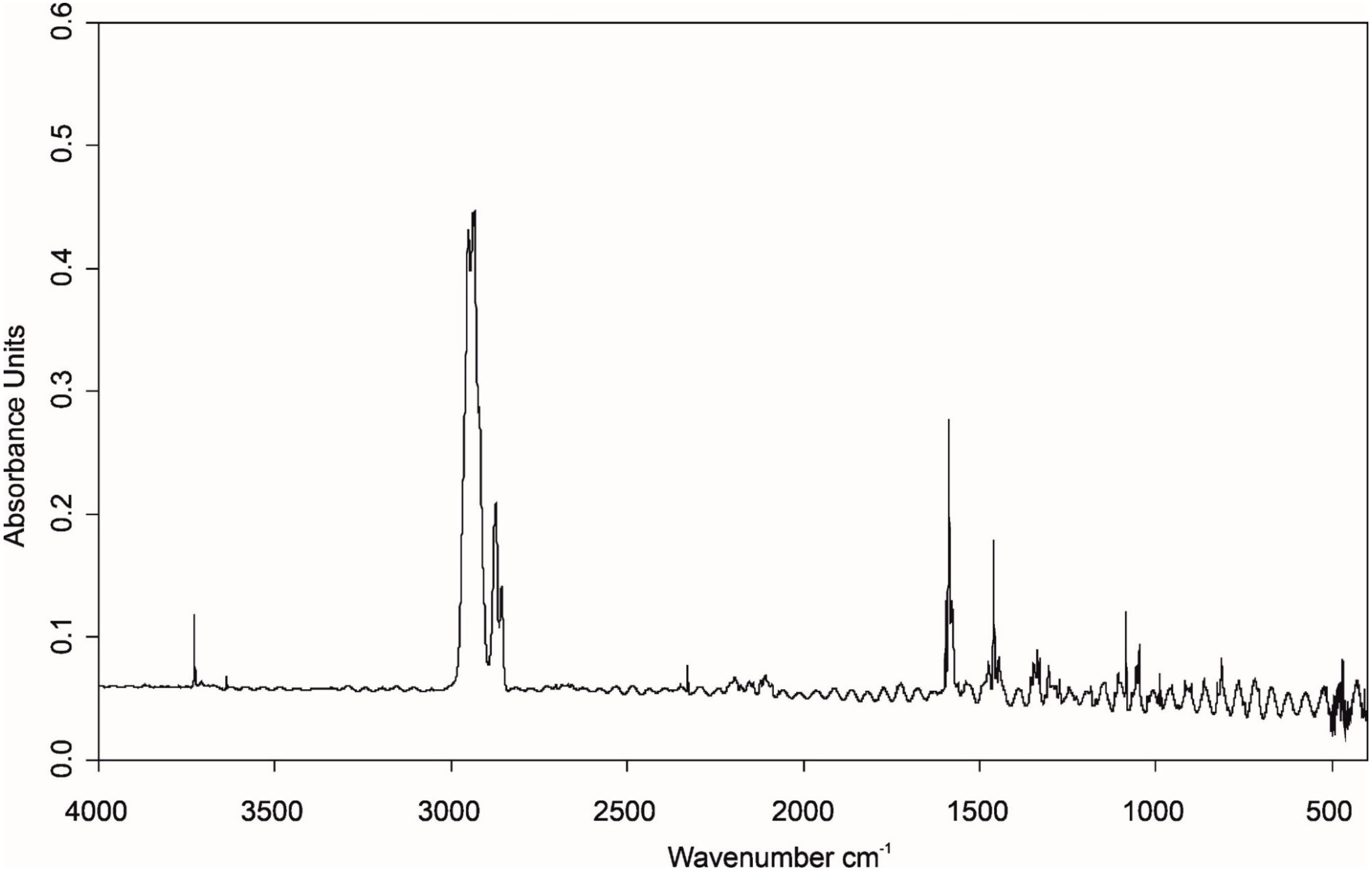

Figure S18: IR spectra showing the deposition of adamantane diazirine (**5**) with subsequent trapping in a nitrogen matrix at 3.5 K.

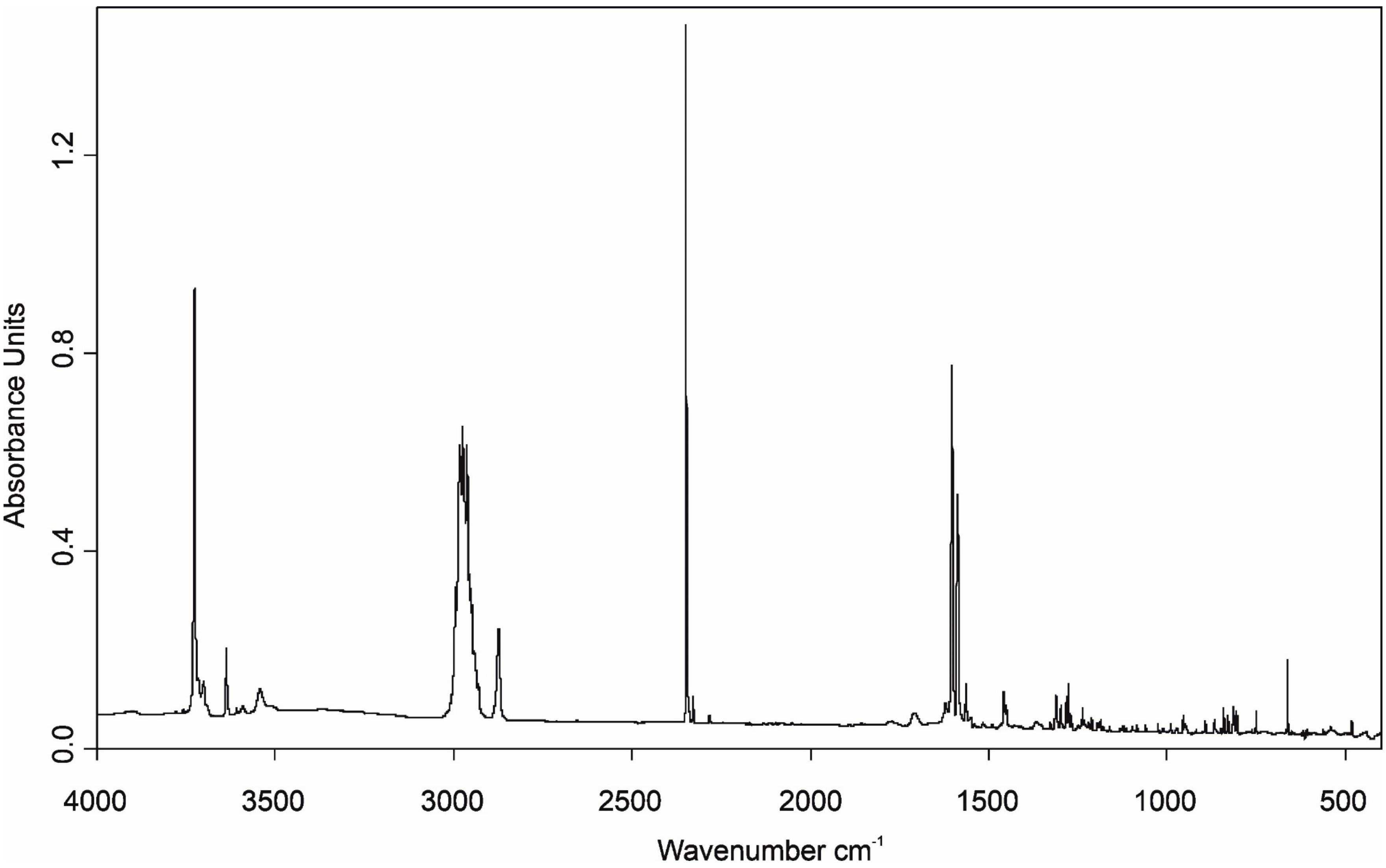

Figure S19: IR spectra showing the deposition of pentacycloundecane diazirine (**6**) with subsequent trapping in a nitrogen matrix at 3.5 K.

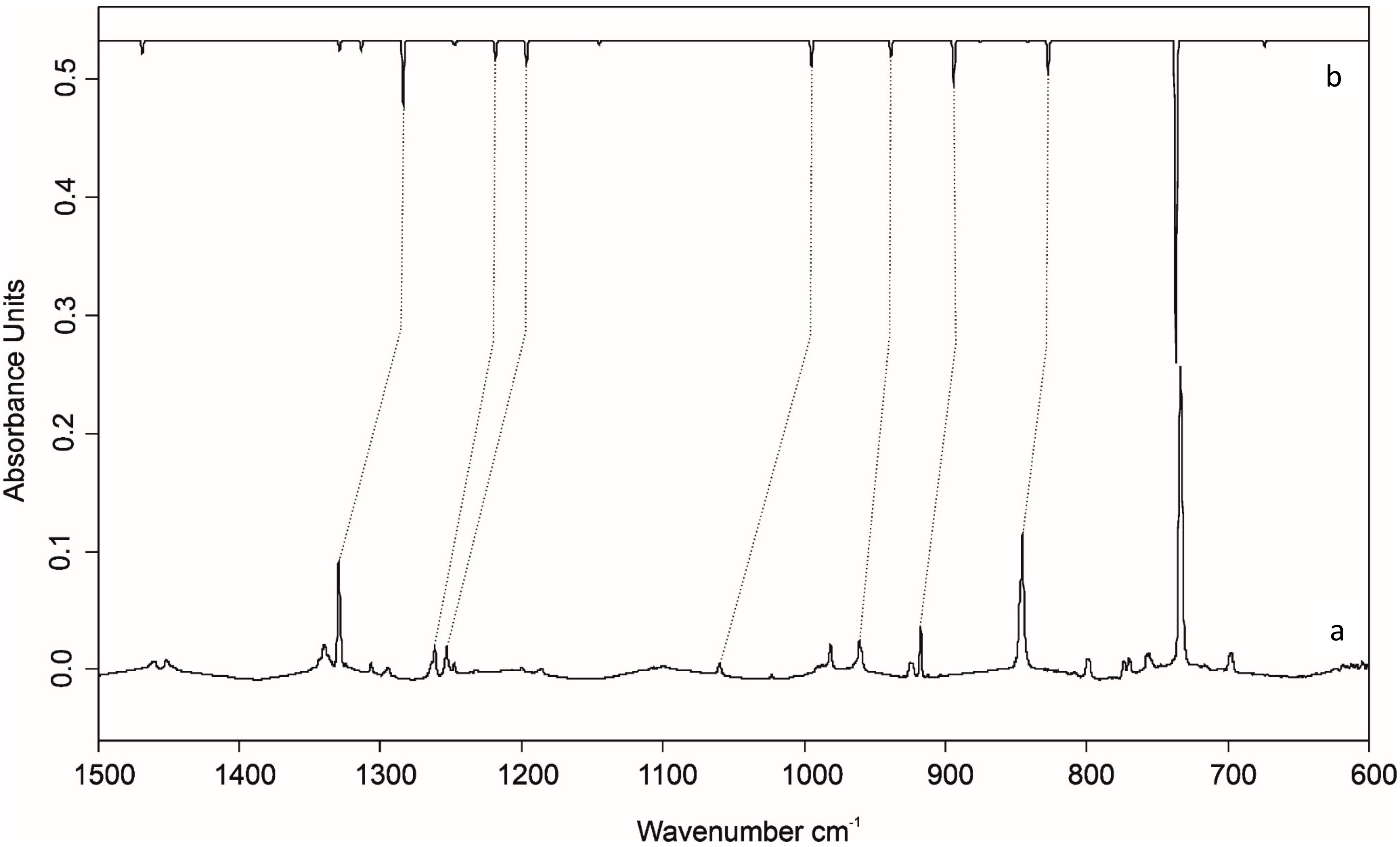

Figure S20: a) IR spectra showing the deposition of **9** with subsequent trapping in a nitrogen matrix at 3.5 K. b) IR spectrum of **9** computed at UB3LYP/6-311++G(3df,2pd) (anharmonic).

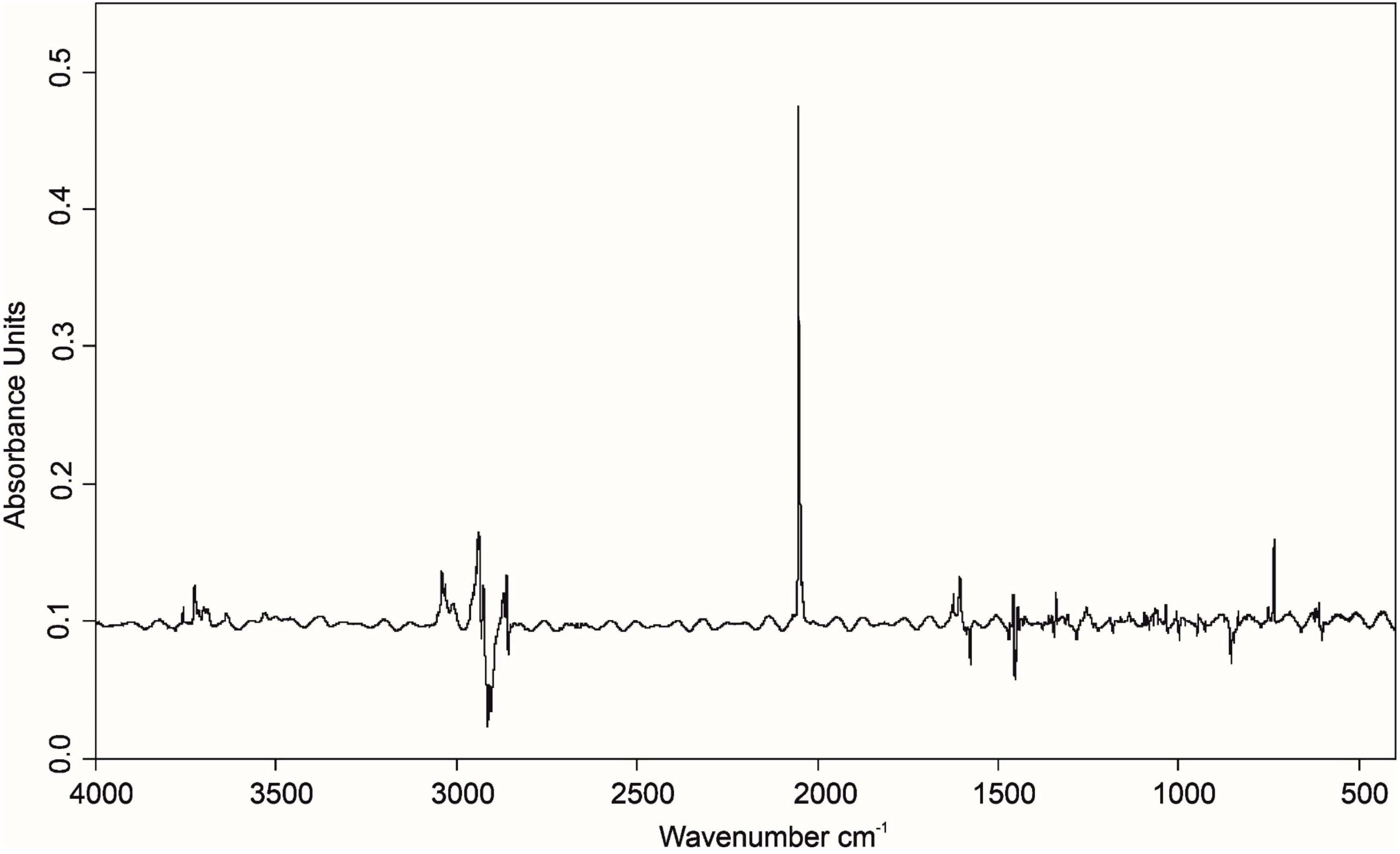

Figure S21: IR difference spectra showing the photochemistry of **5** after irradiation with λ = 365 nm in argon at 3.5 K. Downward bands assigned to **5** disappear after 20 min irradiation. Upward bands assigned to adamantane diazo **26** and **1** appear after 20 min irradiation.

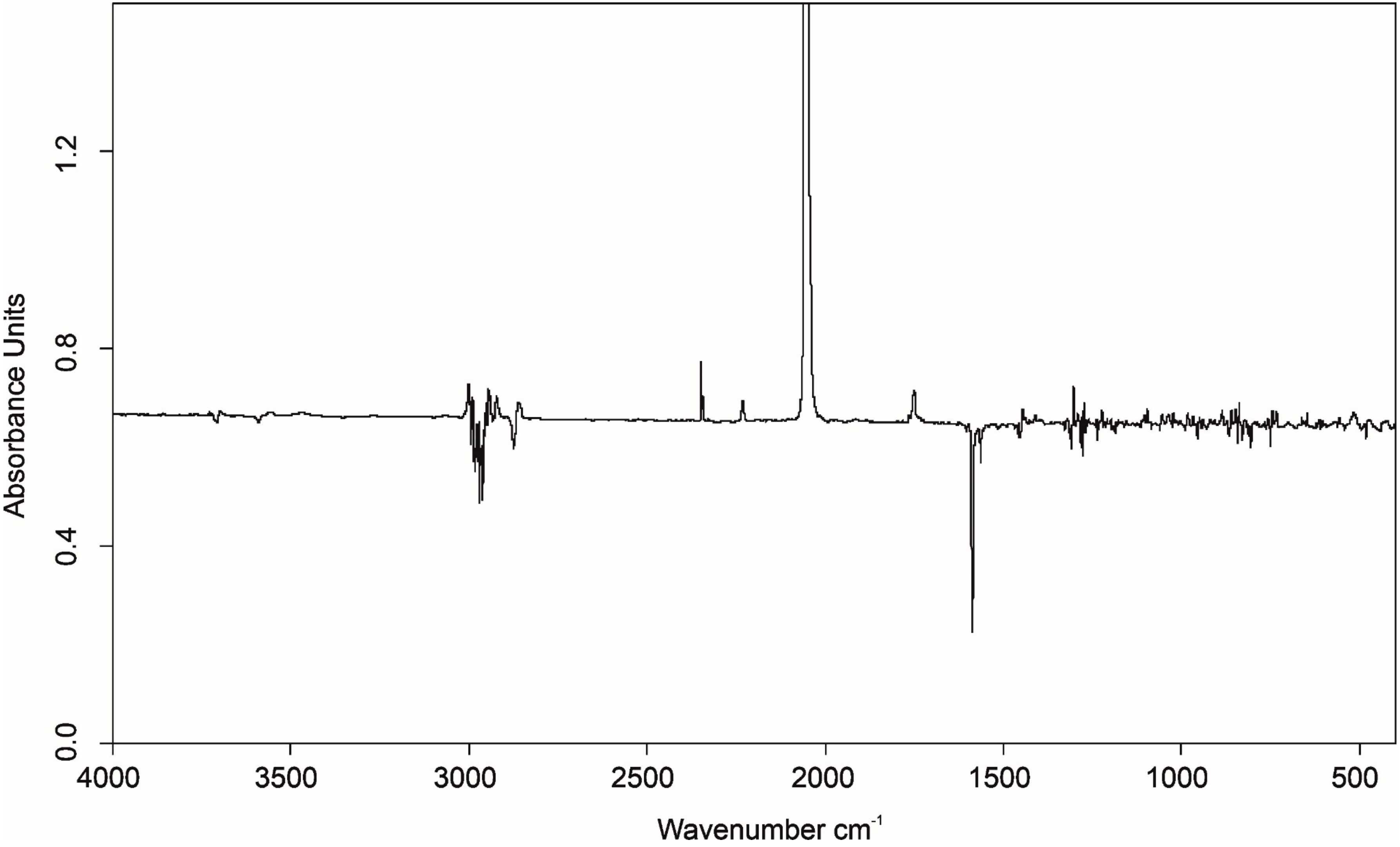

Figure S22: IR difference spectra showing the photochemistry of **6** after irradiation with λ = 365 nm in argon at 3.5 K. Downward bands assigned to **6** disappear after 20 min irradiation. Upward bands assigned to **27** and **2** appear after 20 min irradiation.

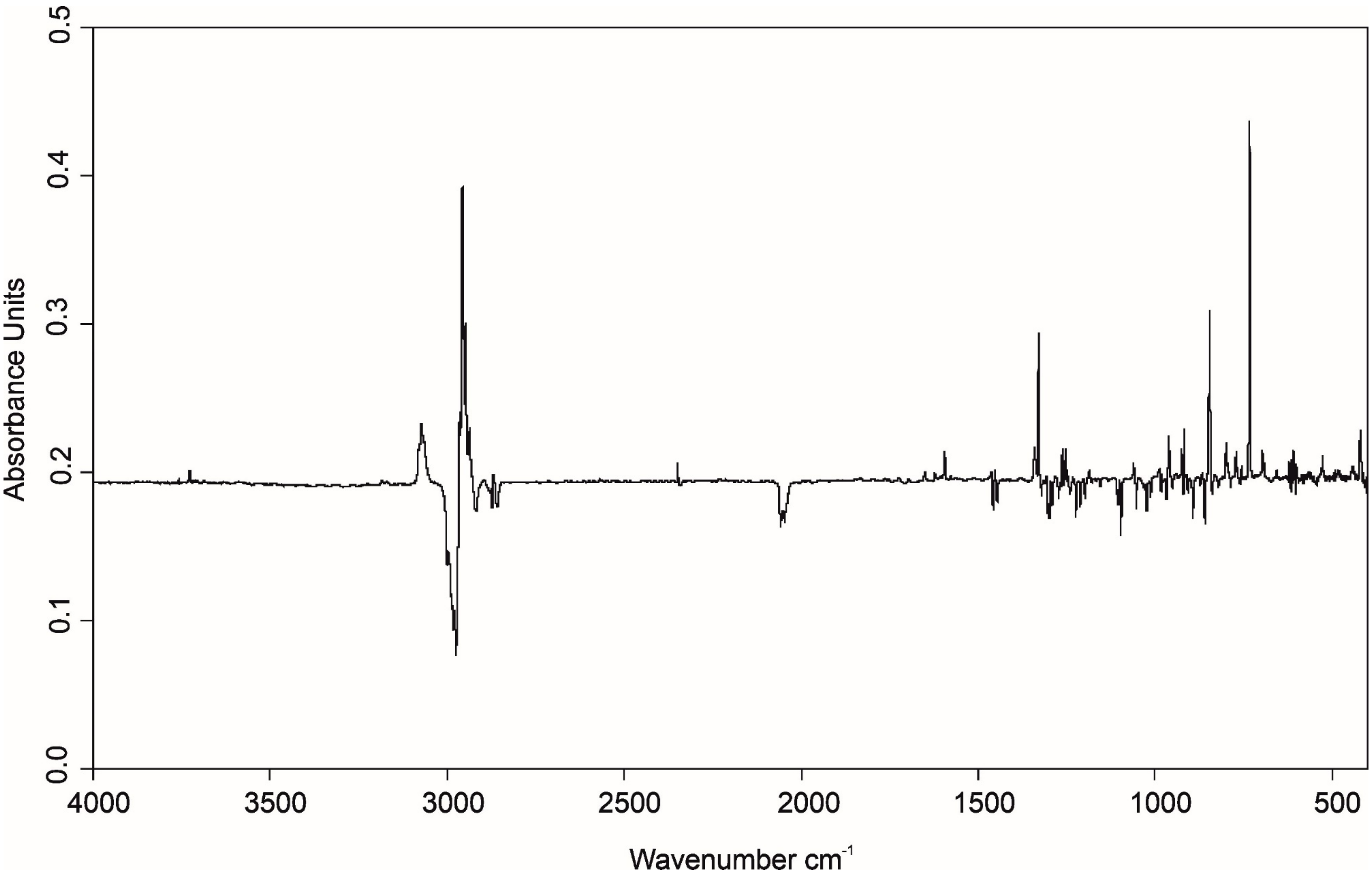

Figure S23: IR difference spectra showing the photochemistry of **2** after irradiating for 10 min at λ = 627 nm in argon at 3.5 K. Downward bands assigned to **2** disappear after irradiation. Upward bands assigned to **9** appear after irradiation.

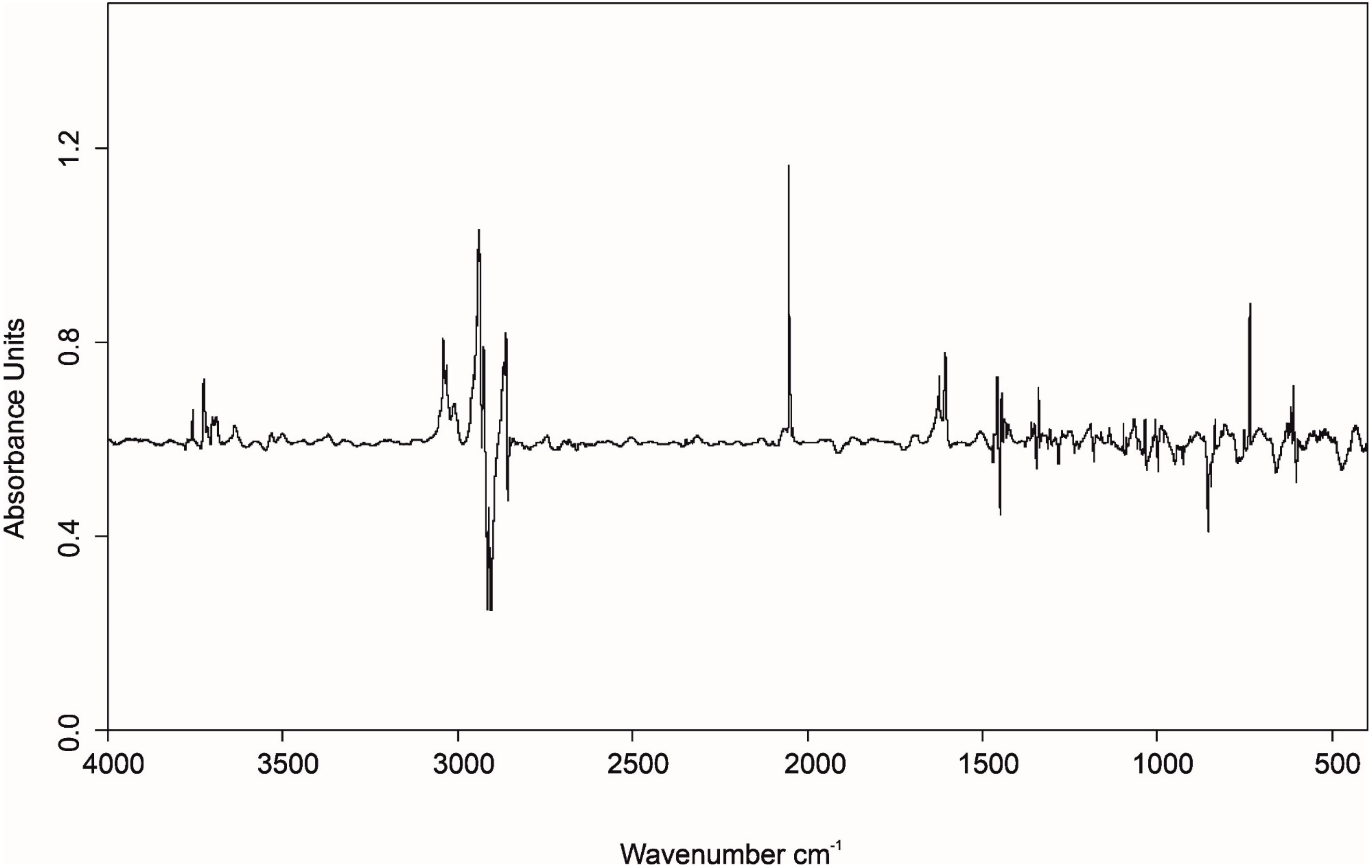

Figure S24: IR difference spectra showing the tunneling of **1** after waiting 16 h in argon at 3.5 K. Downward bands assigned to **1** disappear after 16 h waiting. Upwards bands assigned to **4** appear after 16 h waiting.

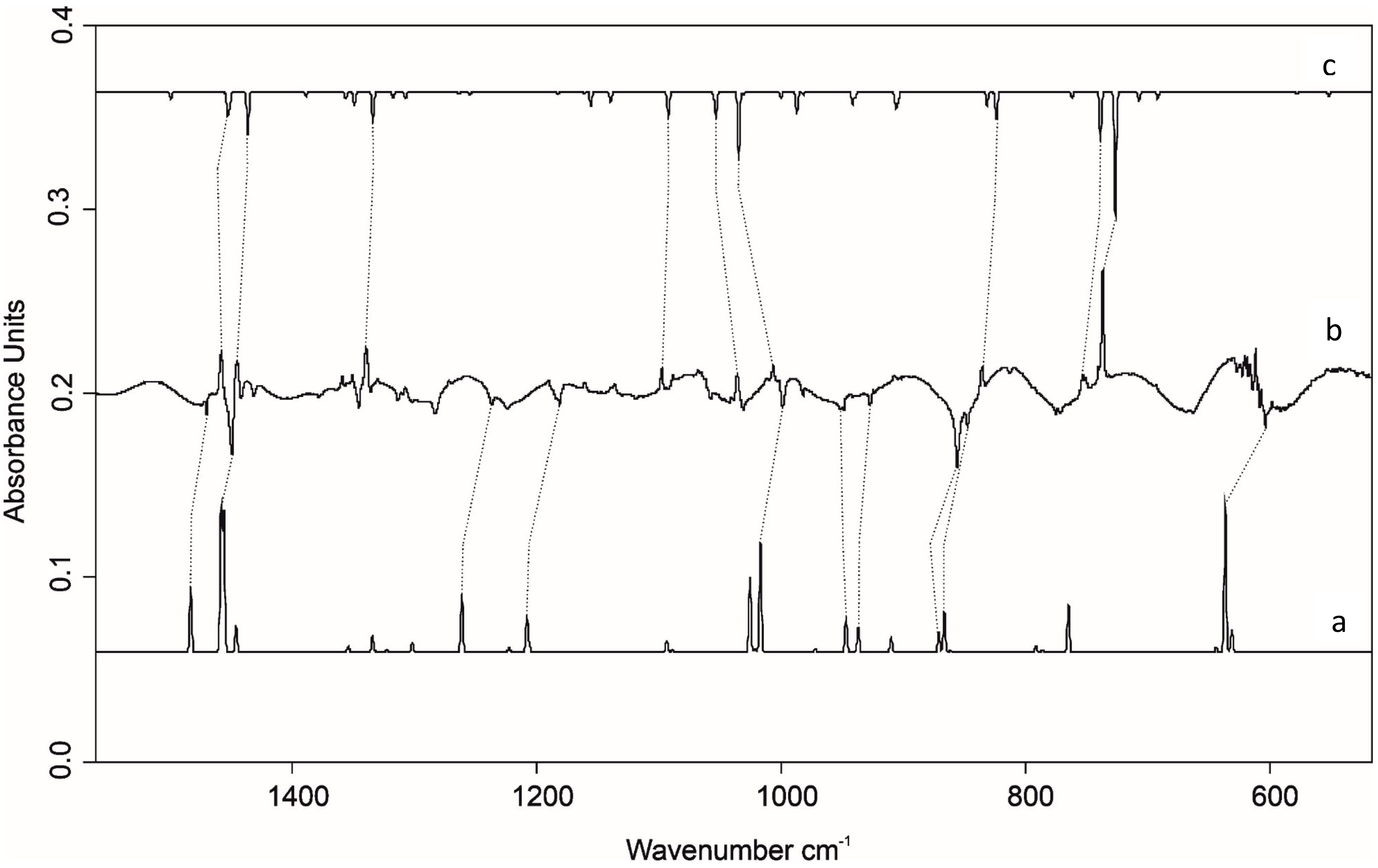

Figure S25: (b) IR difference spectra showing the tunneling of **1** after 16 h in argon at 3.5 K. Downward bands assigned to **1** disappear after 16 h. Upward bands assigned **4** appear after 16 h waiting. (a) IR spectrum of **1** computed at UB3LYP/6-311++G(3df,2pd) (anharmonic). (c) IR spectrum of **4** computed at B3LYP/6-311++G(3df,2pd) (anharmonic).

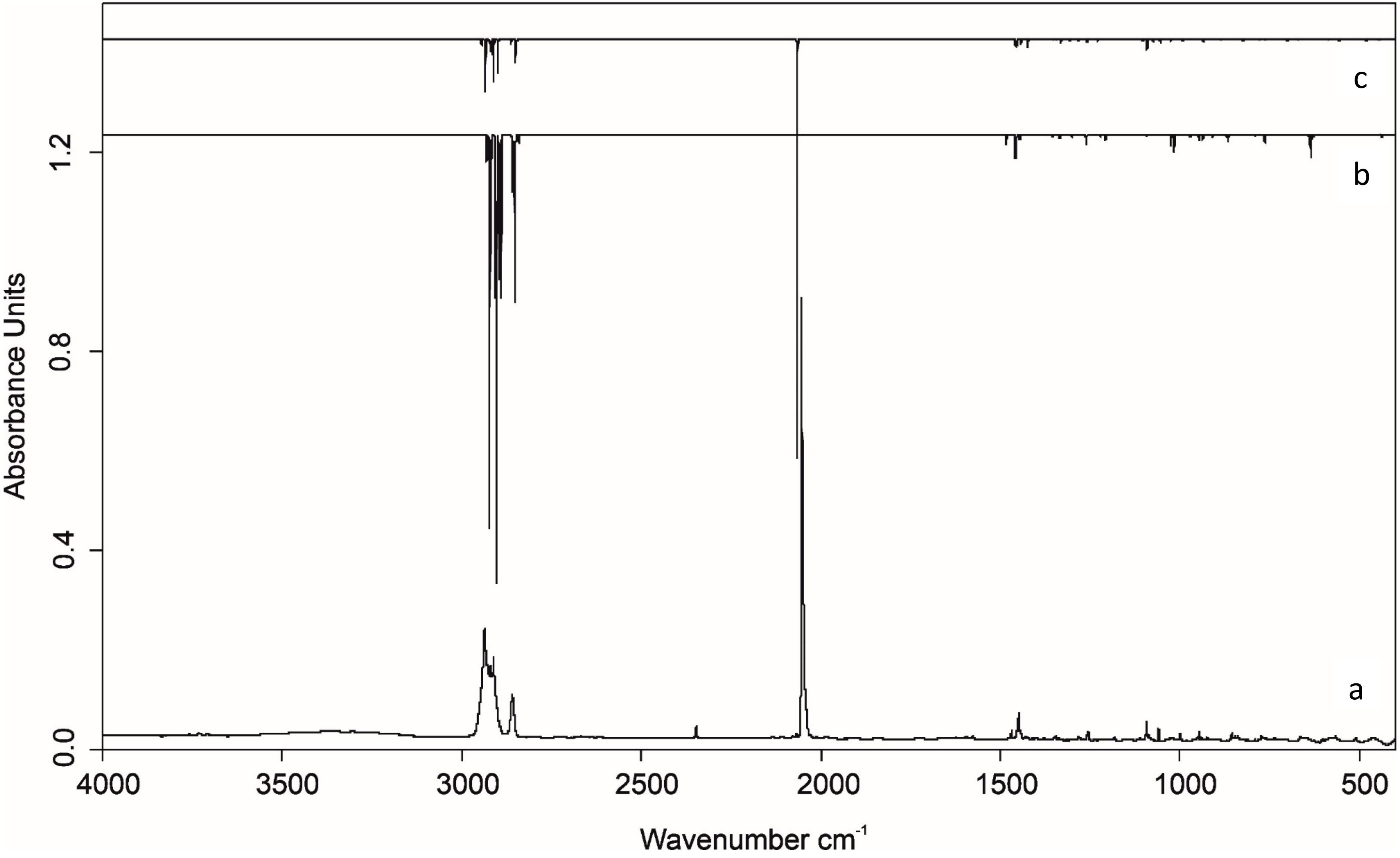

Figure S26: (a) IR spectra showing the photochemistry of **1** after irradiation with λ = 365 nm in argon at 3.5 K. Bands assigned to adamantane diazo **26** and **1** appear after 20 min irradiation. (b) IR spectrum of **1** computed at UB3LYP/6-311++G(3df,2pd) (anharmonic). (c) IR spectrum of **26** computed at B3LYP/6-311++G(3df,2pd) (anharmonic).

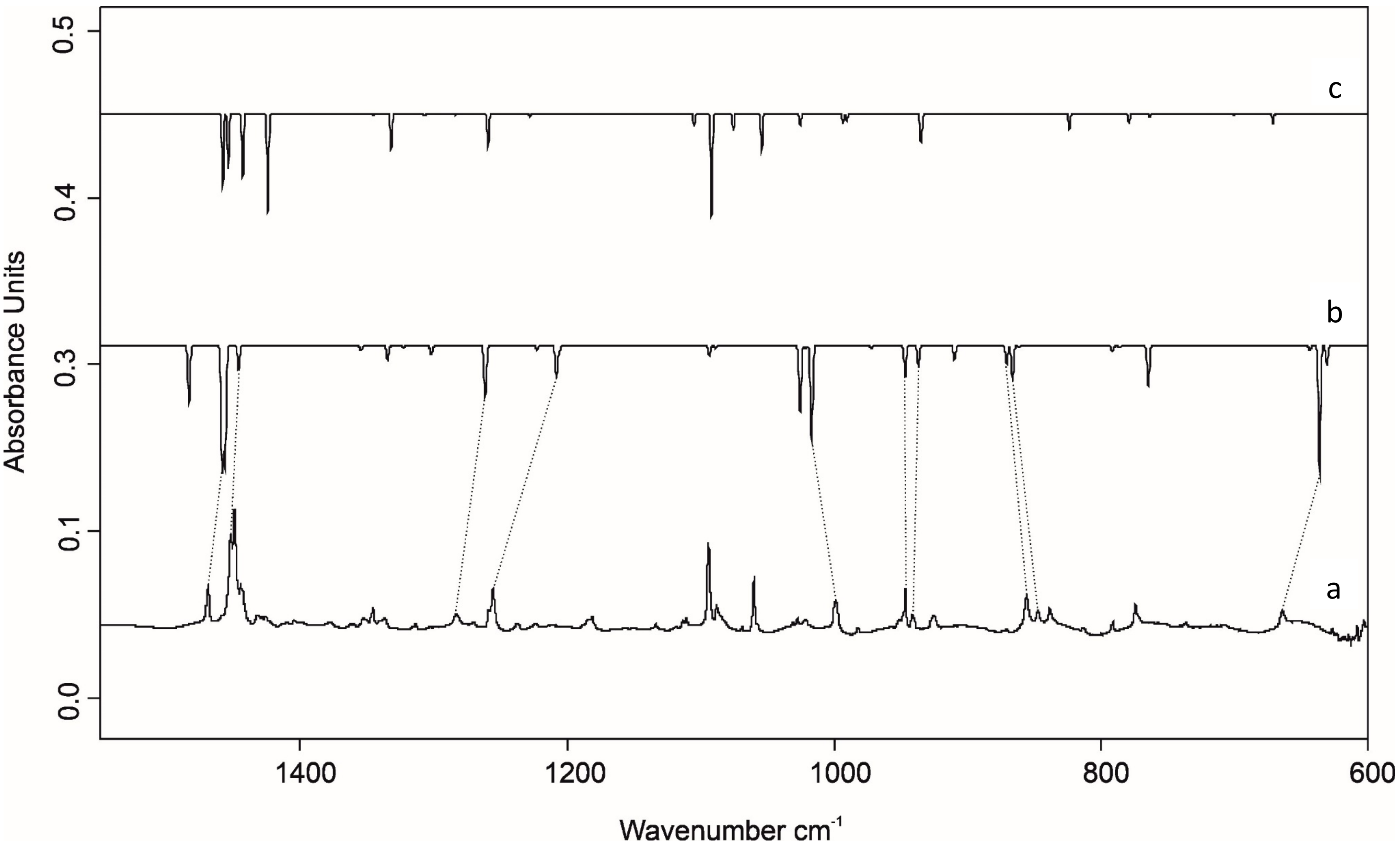

Figure S27: (a) IR spectra showing the photochemistry of **1** after irradiation with λ = 365 nm in argon at 3.5 K. Bands assigned to adamantane diazo **26** and **1** appear after 20 min irradiation. The bands to compound **1** are assigned. (b) IR spectrum of **1** computed at UB3LYP/6-311++G(3df,2pd) (anharmonic). (c) IR spectrum of **26** computed at B3LYP/6-311++G(3df,2pd) (anharmonic).

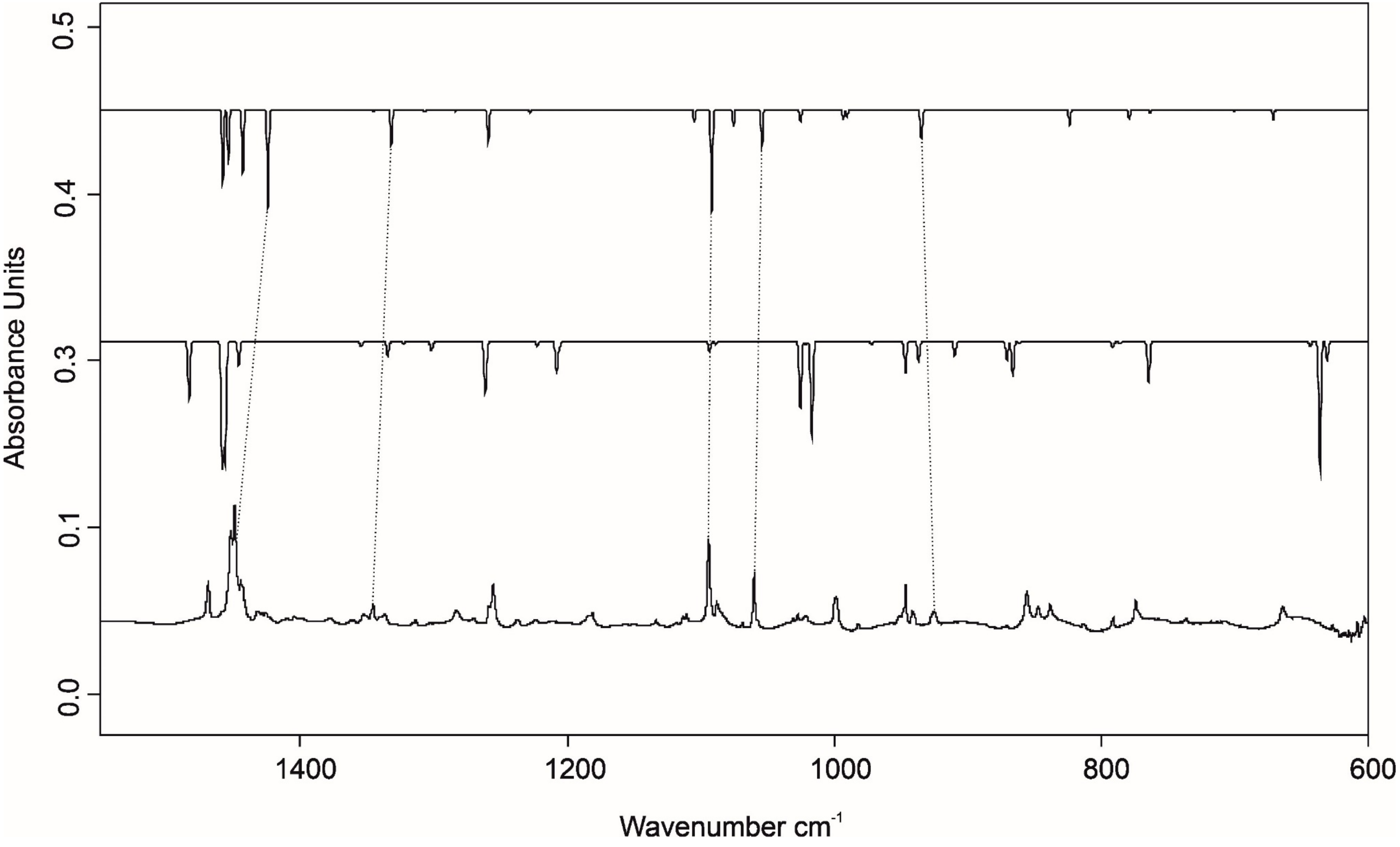

Figure S28: (a) IR spectra showing the photochemistry of **1** after irradiation with λ = 365 nm in argon at 3.5 K. Bands assigned to adamantane diazo **26** and **1** appear after 20 min irradiation. The bands to compound **26** are assigned. (b) IR spectrum of **1** computed at UB3LYP/6-311++G(3df,2pd) (anharmonic). (c) IR spectrum of **26** computed at B3LYP/6-311++G(3df,2pd) (anharmonic).

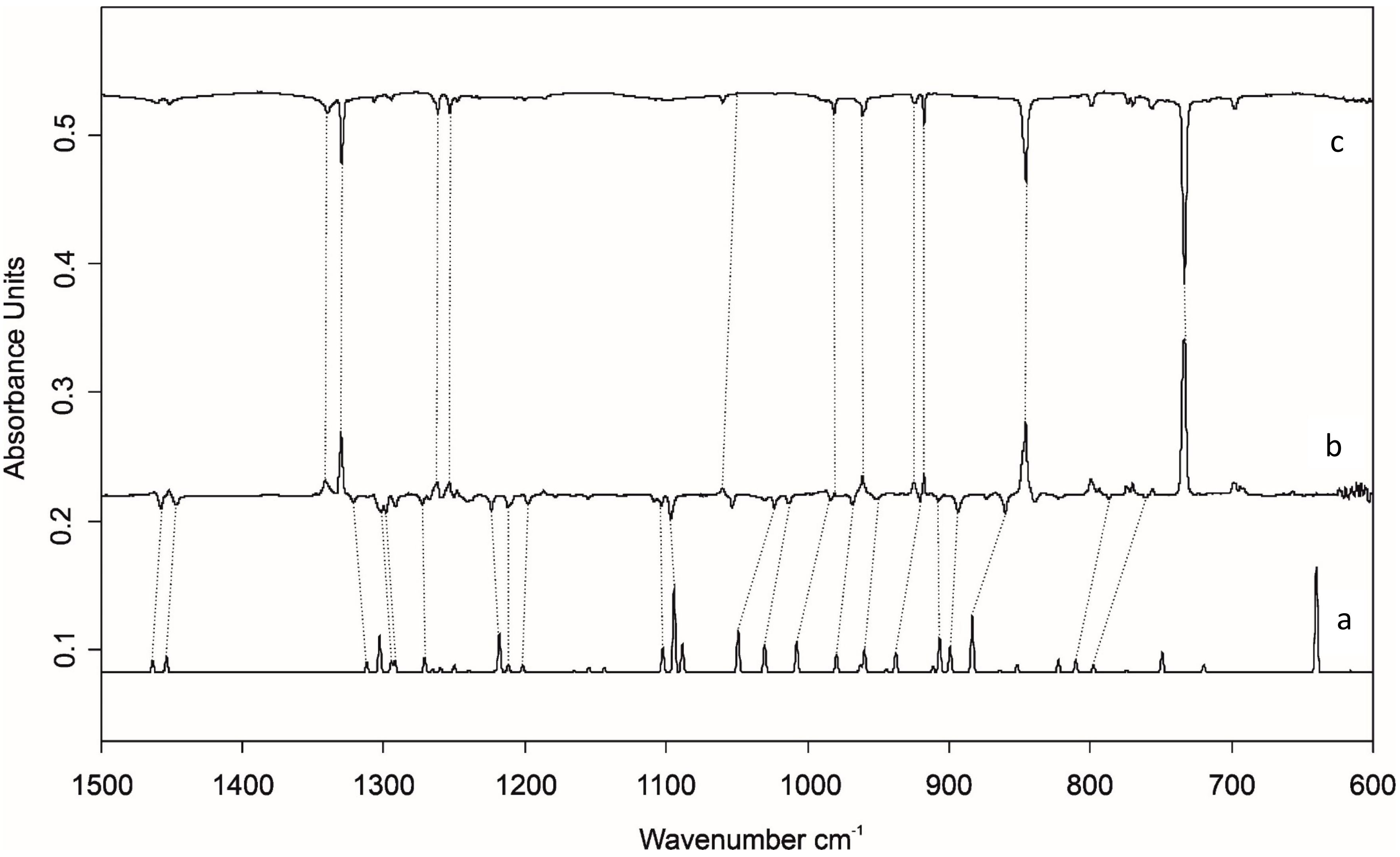

Figure S29: (b) IR difference spectra showing the photochemistry of **2** after irradiating for 10 min at λ = 627 nm in argon at 3.5 K. Downward bands assigned to **2** disappear after irradiation. Upward bands assigned **9** appear after irradiation. (a) IR spectrum of **2** computed at B3LYP/6-311++G(3df,2pd) (anharmonic). (c) IR spectra showing the deposition of **9** with subsequent trapping in a nitrogen matrix at 3.5 K.

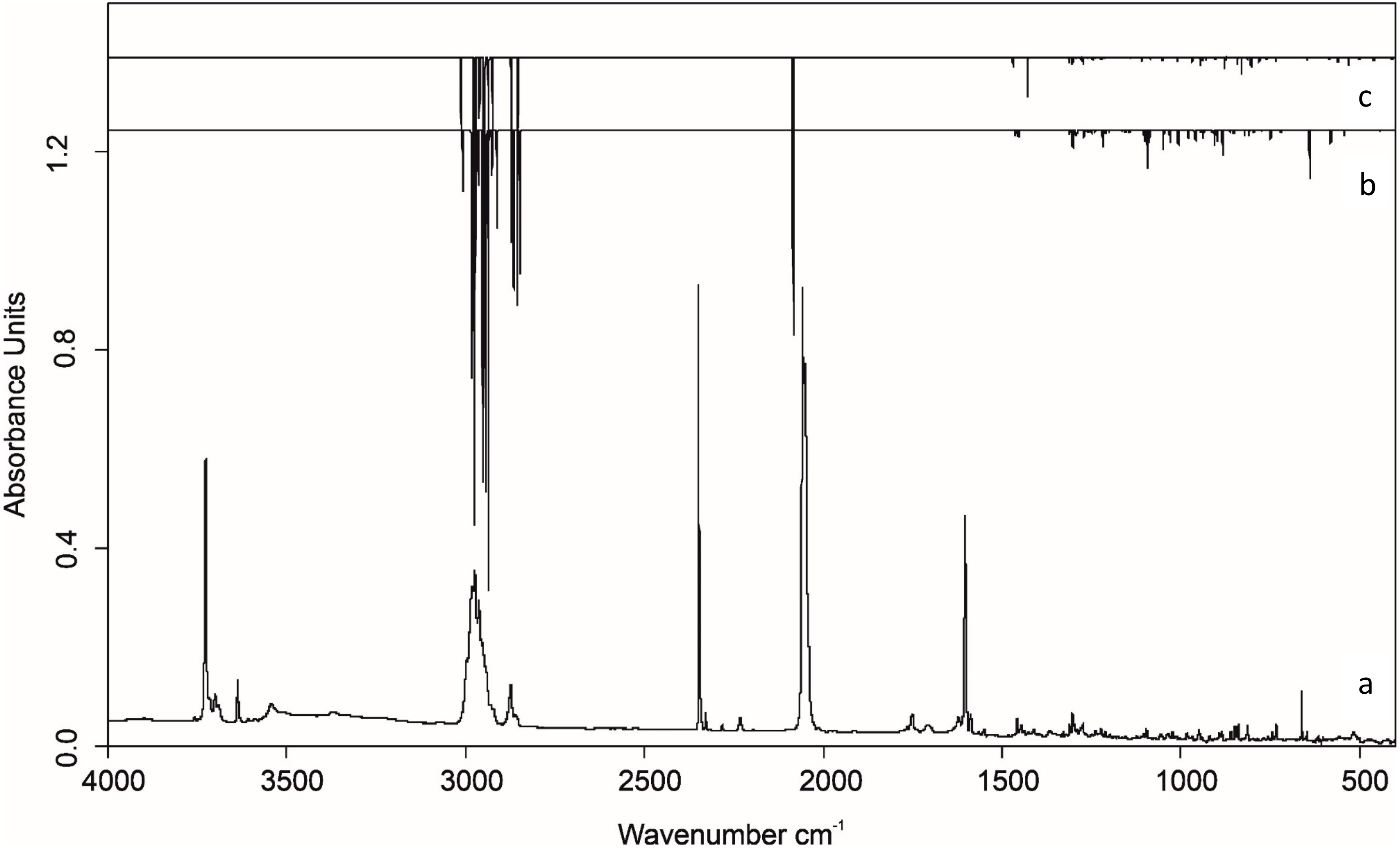

Figure S30: (a) IR spectra showing the photochemistry of **2** after irradiation with λ = 365 nm in argon at 3.5 K. Bands assigned to adamantane diazo **27** and **2** appear after 20 min irradiation. (b) IR spectrum of **2** computed at UB3LYP/6-311++G(3df,2pd) (anharmonic). (c) IR spectrum of **27** computed at B3LYP/6-311++G(3df,2pd) (anharmonic).

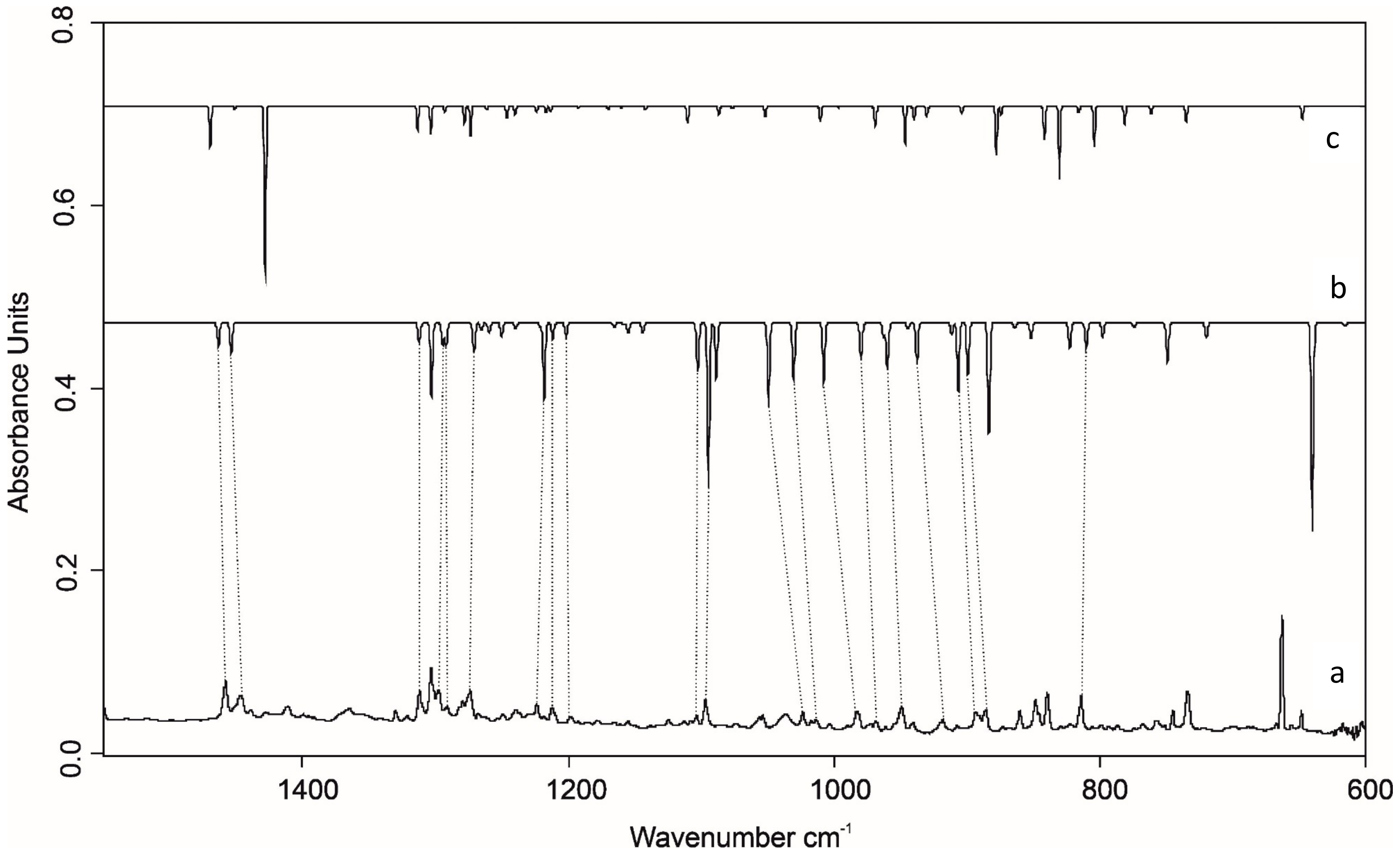

Figure S31: (a) IR spectra showing the photochemistry of **2** after irradiation with λ = 365 nm in argon at 3.5 K. Bands assigned to adamantane diazo **27** and **2** appear after 20 min irradiation. The bands to compound **2** are assigned. (b) IR spectrum of **2** computed at UB3LYP/6-311++G(3df,2pd) (anharmonic). (c) IR spectrum of **27** computed at B3LYP/6-311++G(3df,2pd) (anharmonic).

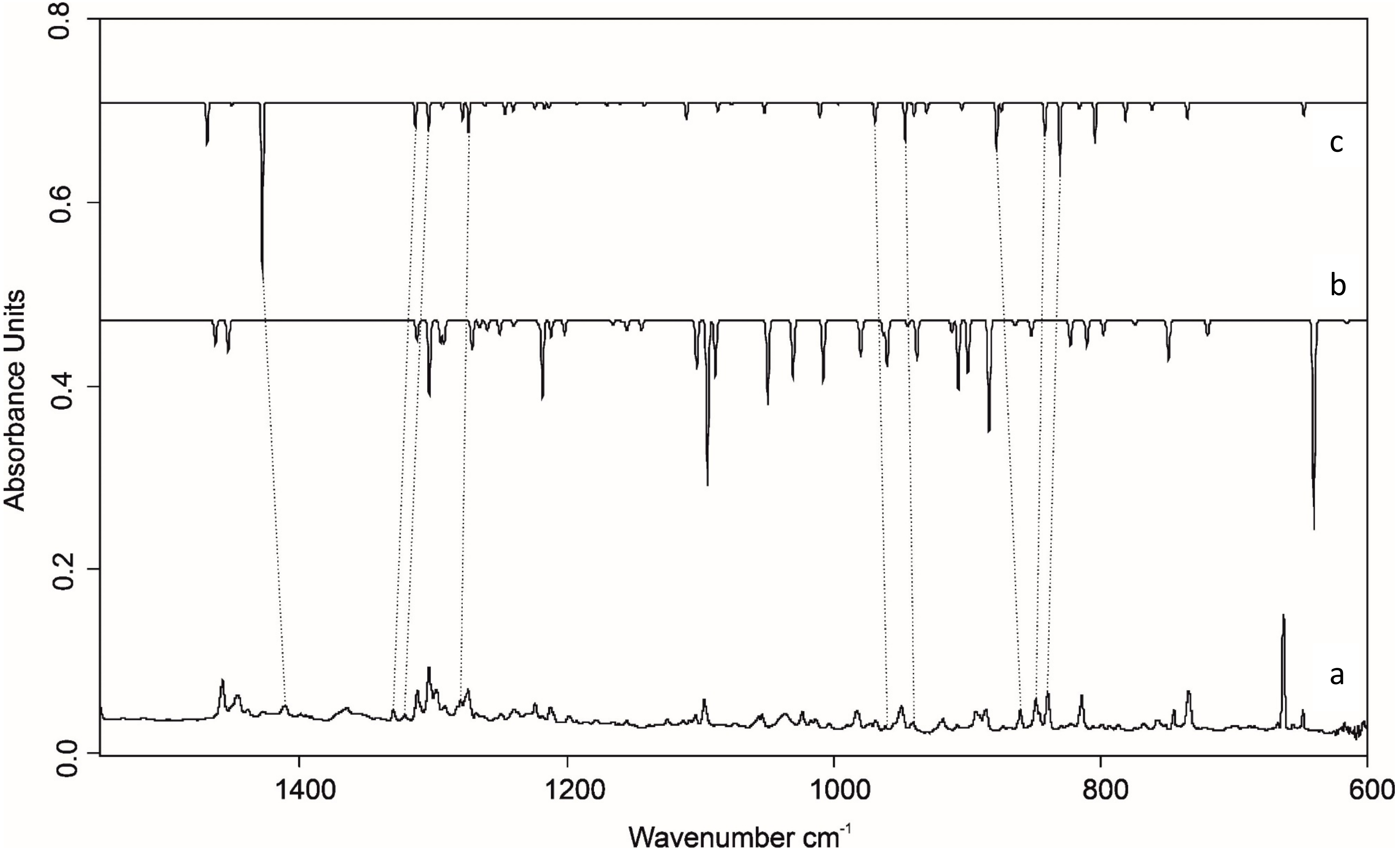

Figure S32: (a) IR spectra showing the photochemistry of **2** after irradiation with λ = 365 nm in argon at 3.5 K. Bands assigned to adamantane diazo **27** and **2** appear after 20 min irradiation. The bands to compound **27** are assigned. (b) IR spectrum of **2** computed at UB3LYP/6-311++G(3df,2pd) (anharmonic). (c) IR spectrum of **27** computed at B3LYP/6-311++G(3df,2pd) (anharmonic).

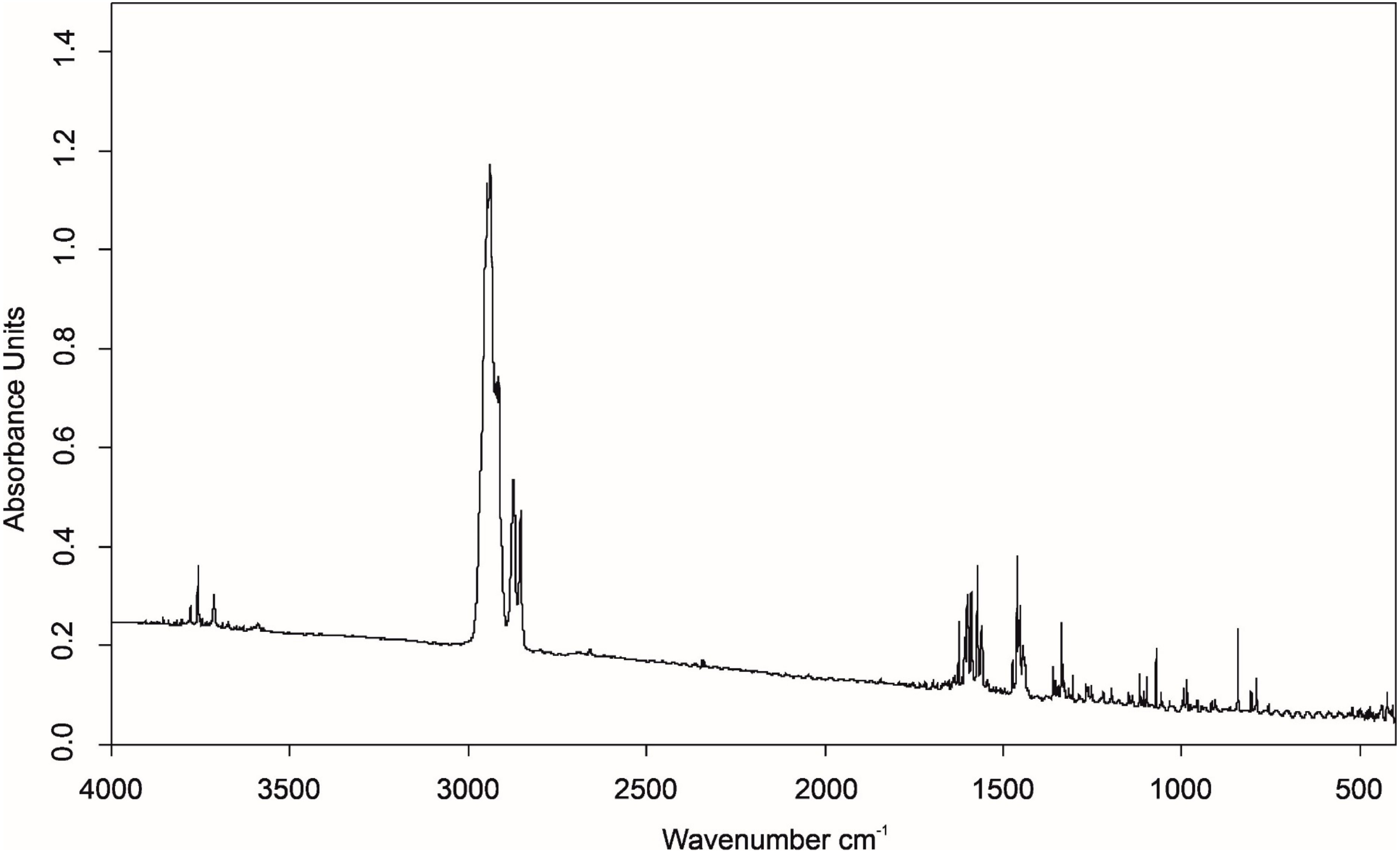

Figure S33: IR spectra showing the deposition of protoadamantane diazirine (**13**) with subsequent trapping in a nitrogen matrix at 3.5 K.

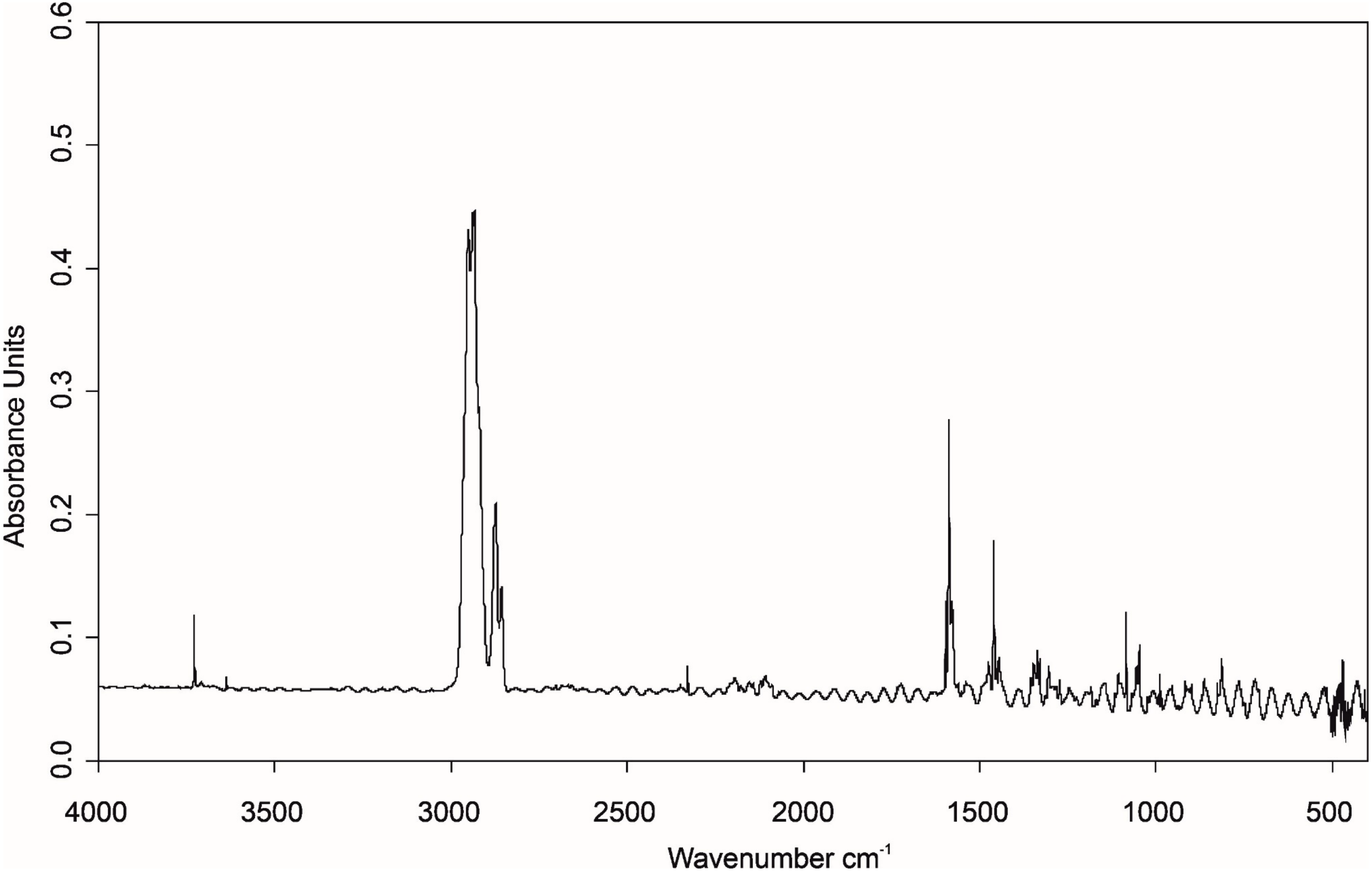

Figure S34: IR spectra showing the deposition of d_2-protoadamantane diazirine (d_2-**13**) with subsequent trapping in a nitrogen matrix at 3.5 K.

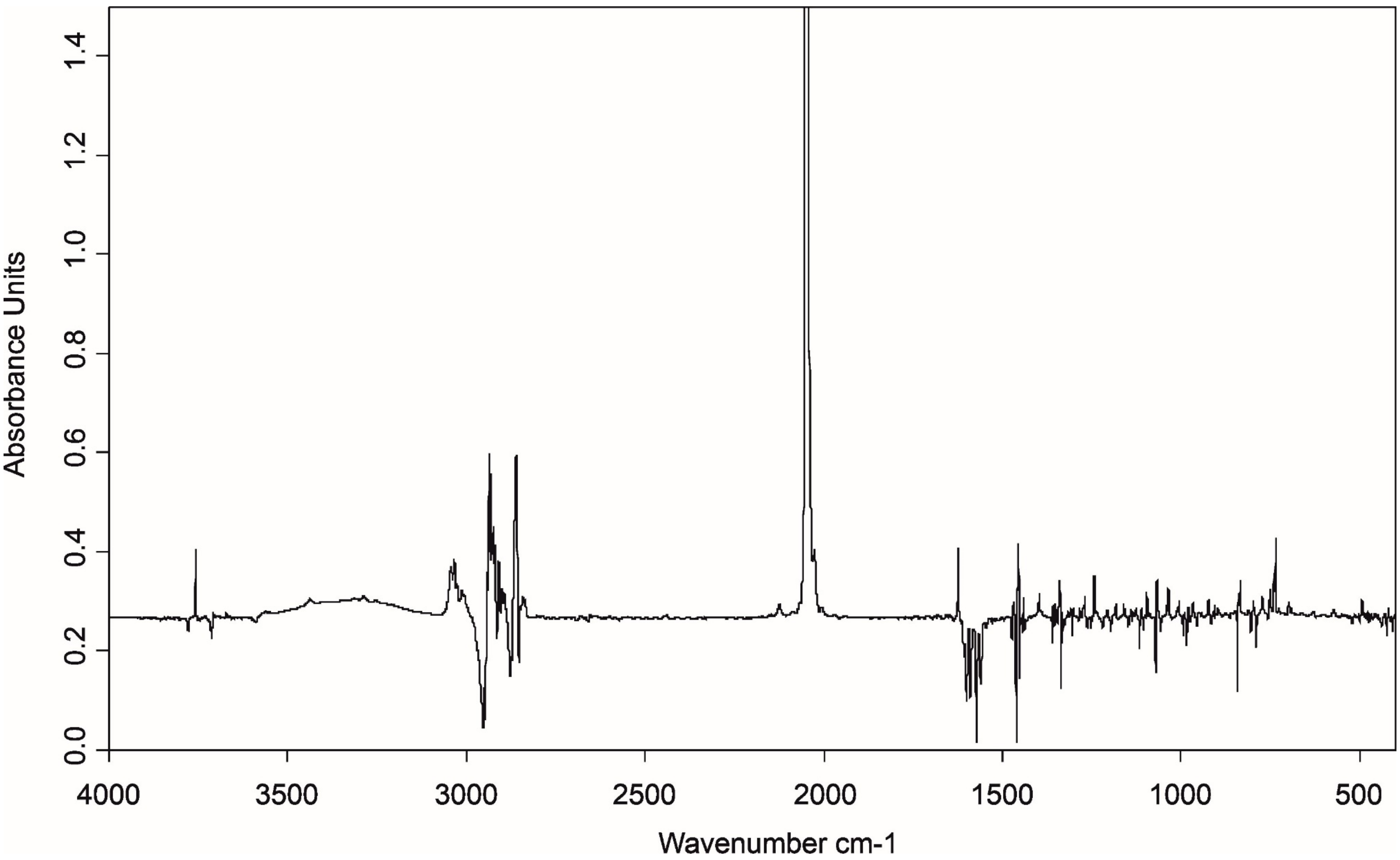

Figure S35: IR difference spectra showing the photochemistry of **13** after irradiation with λ = 365 nm in argon at 3.5 K. Downward bands assigned to **13** disappear after 20 min irradiation. Upward bands assigned to protoadamantane diazo (**28**) appear after 20 min irradiation.

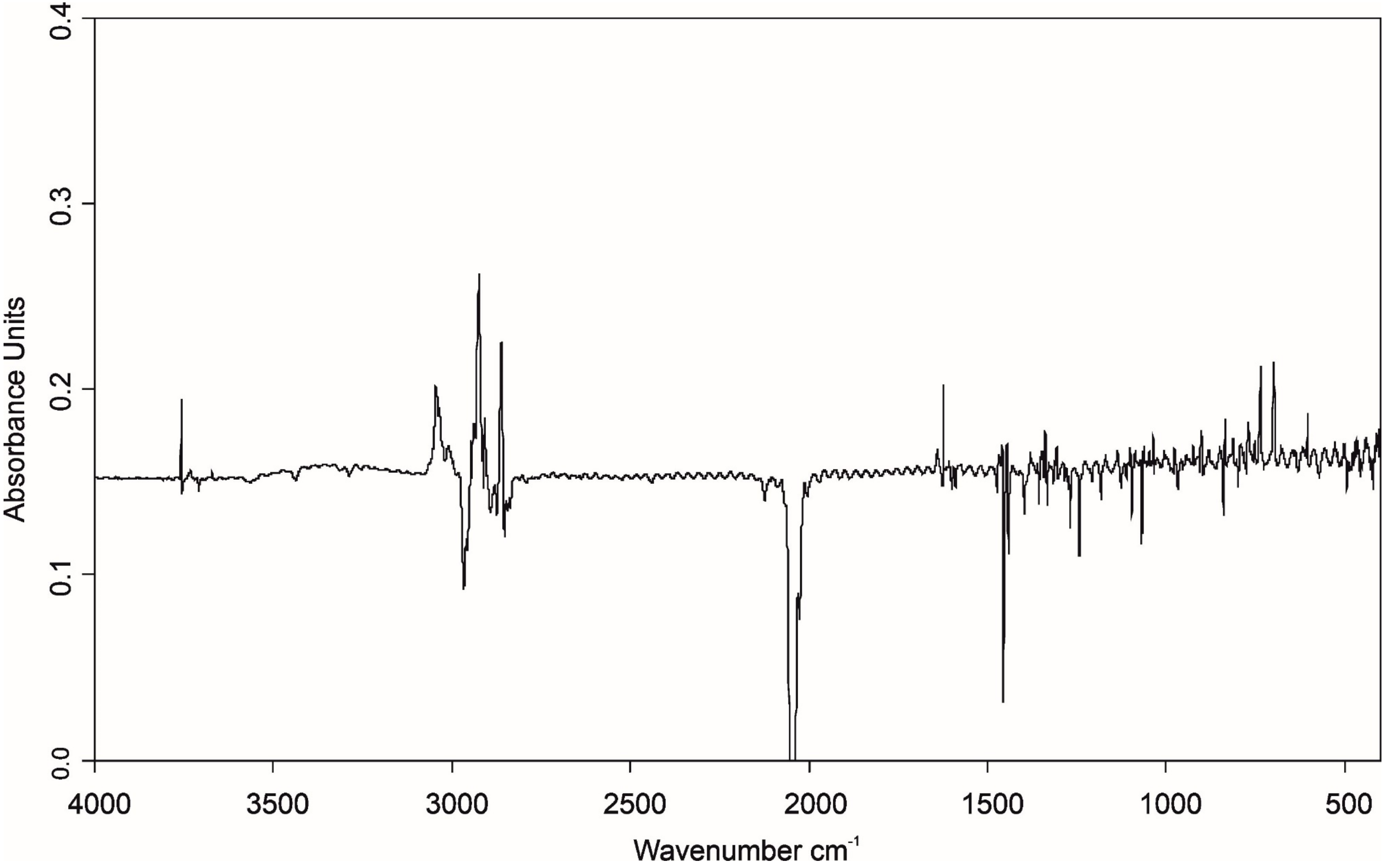

Figure S36: IR difference spectra showing the photochemistry of **28** after irradiation with λ = 254 nm in argon at 3.5 K. Downward bands assigned to **28** disappear after 20 min irradiation. Upward bands assigned to **4** and **12** appear after 20 min irradiation.

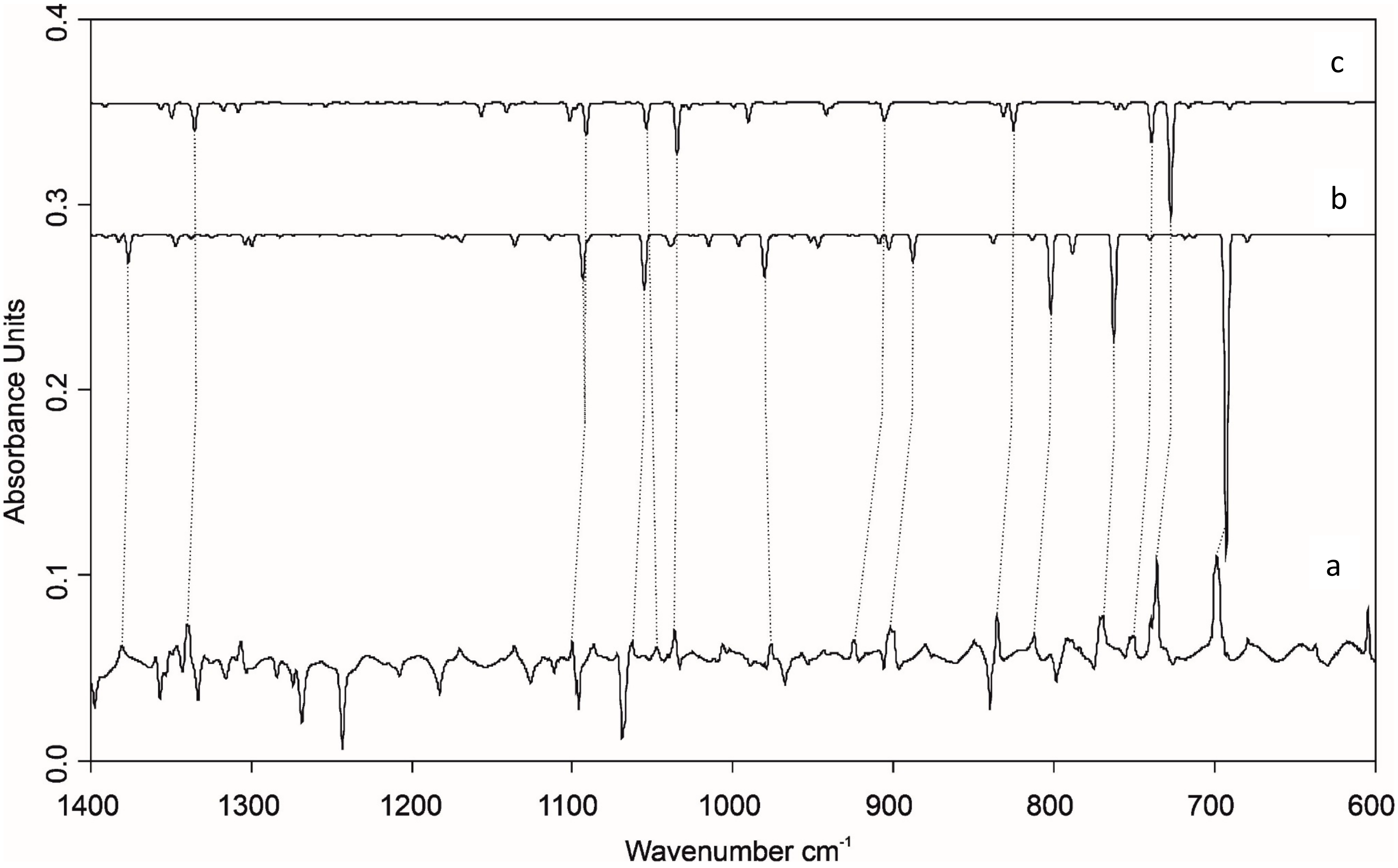

Figure S37: (a) IR difference spectra showing the photochemistry of **28** after irradiation with λ = 254 nm in argon at 3.5 K. Downward bands assigned to **28** disappear after 20 min irradiation. Upward bands assigned to **12** and **4** appear after 20 min irradiation. (b) IR spectrum of **12** computed at B3LYP/6-311++G(3df,2pd) (anharmonic). (c) IR spectrum of **4** computed at B3LYP/6-311++G(3df,2pd) (anharmonic).

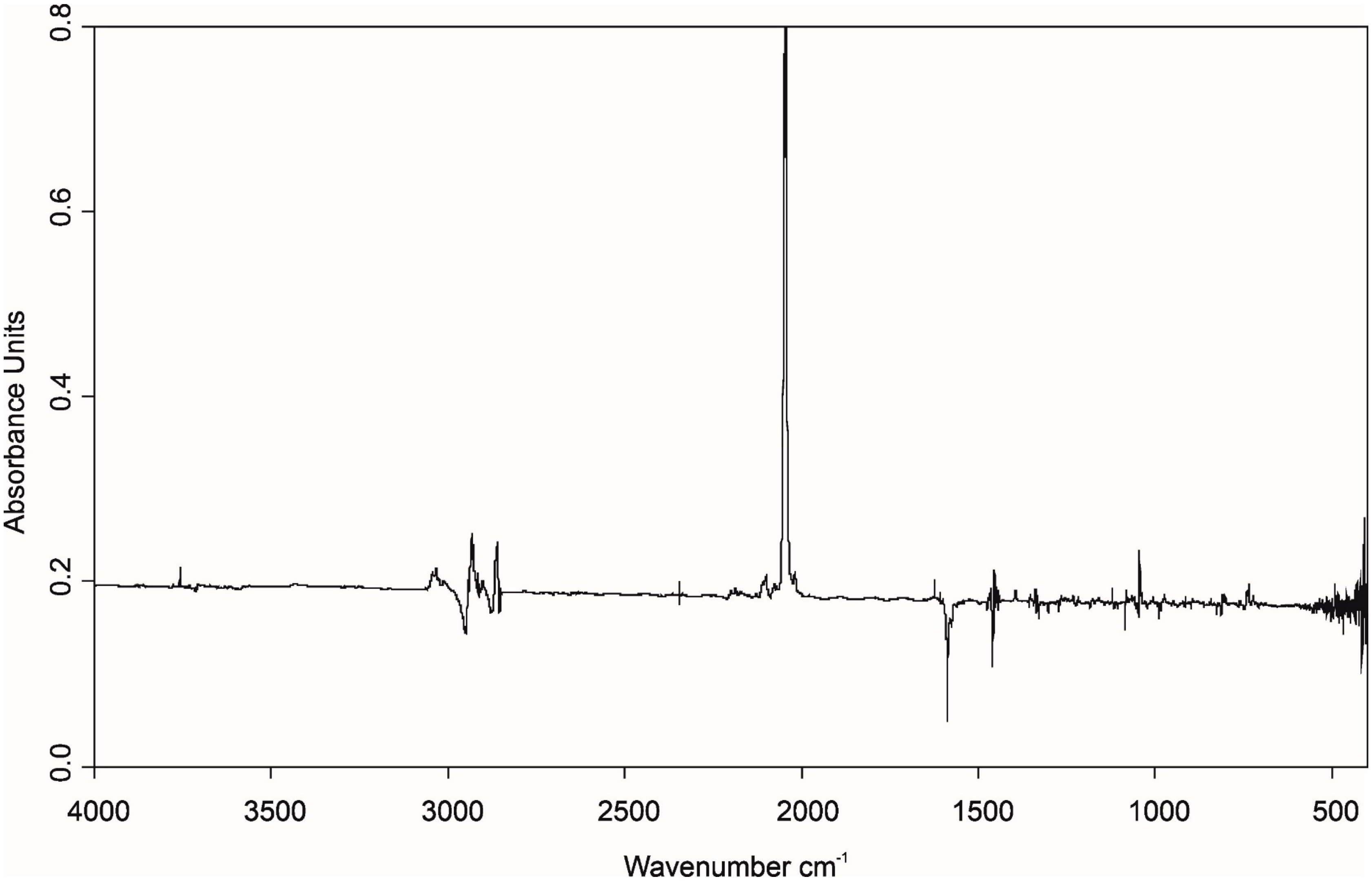

Figure S38: IR difference spectra showing the photochemistry of d_2-**28** after irradiation with λ = 365 nm in argon at 3.5 K. Downward bands assigned to d_2-**28** disappear after 20 min irradiation. Upward bands assigned to d_2-**28** appear after 20 min irradiation.

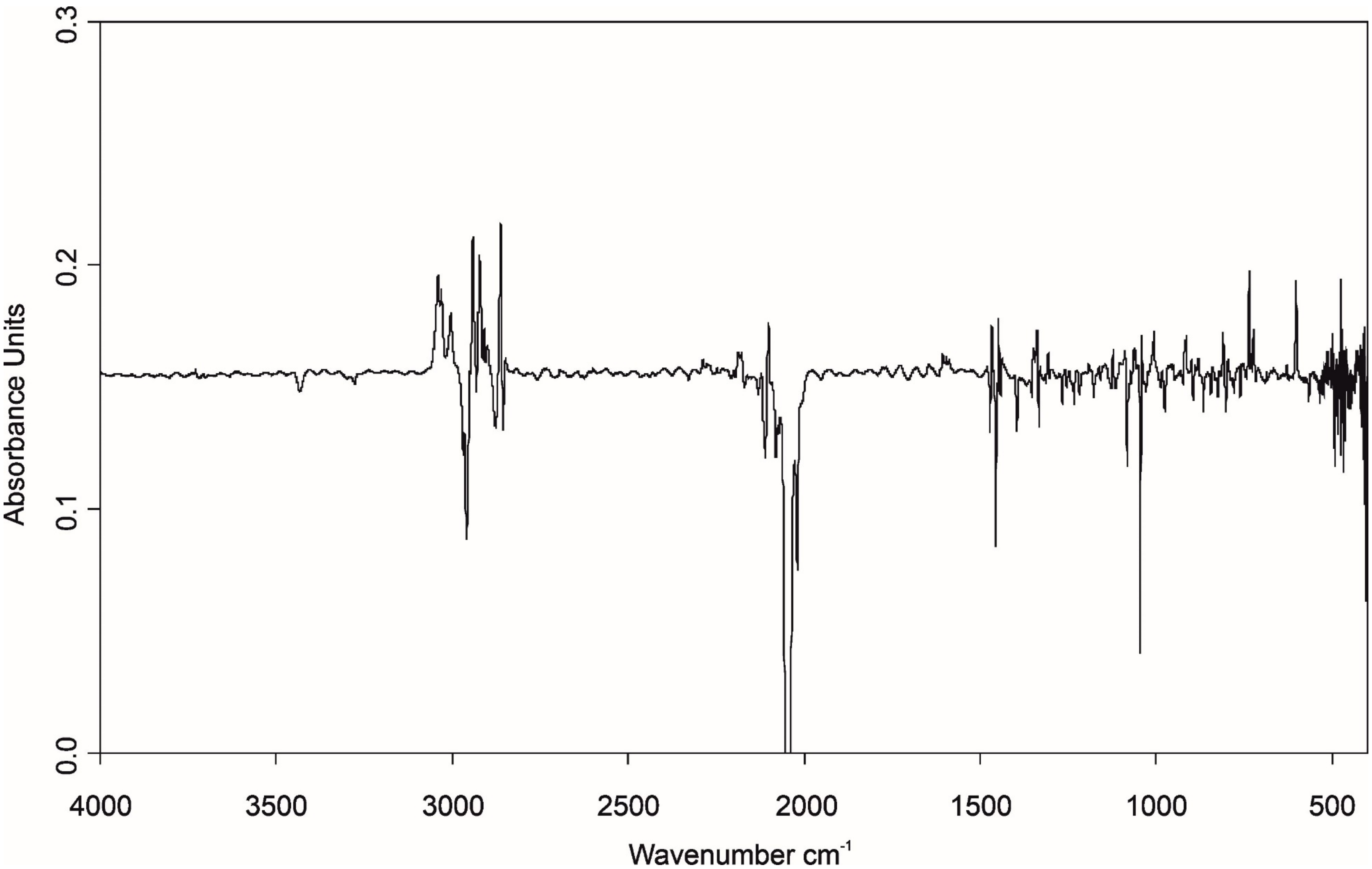

Figure S39: IR difference spectra showing the photochemistry of d_2-**28** after irradiation with λ = 254 nm in argon at 3.5 K. Downward bands assigned to d_2-protoadamantane diazo disappear after 20 min irradiation. Upward bands assigned to d_2-**12** and d_2-**4** appear after 20 min irradiation.

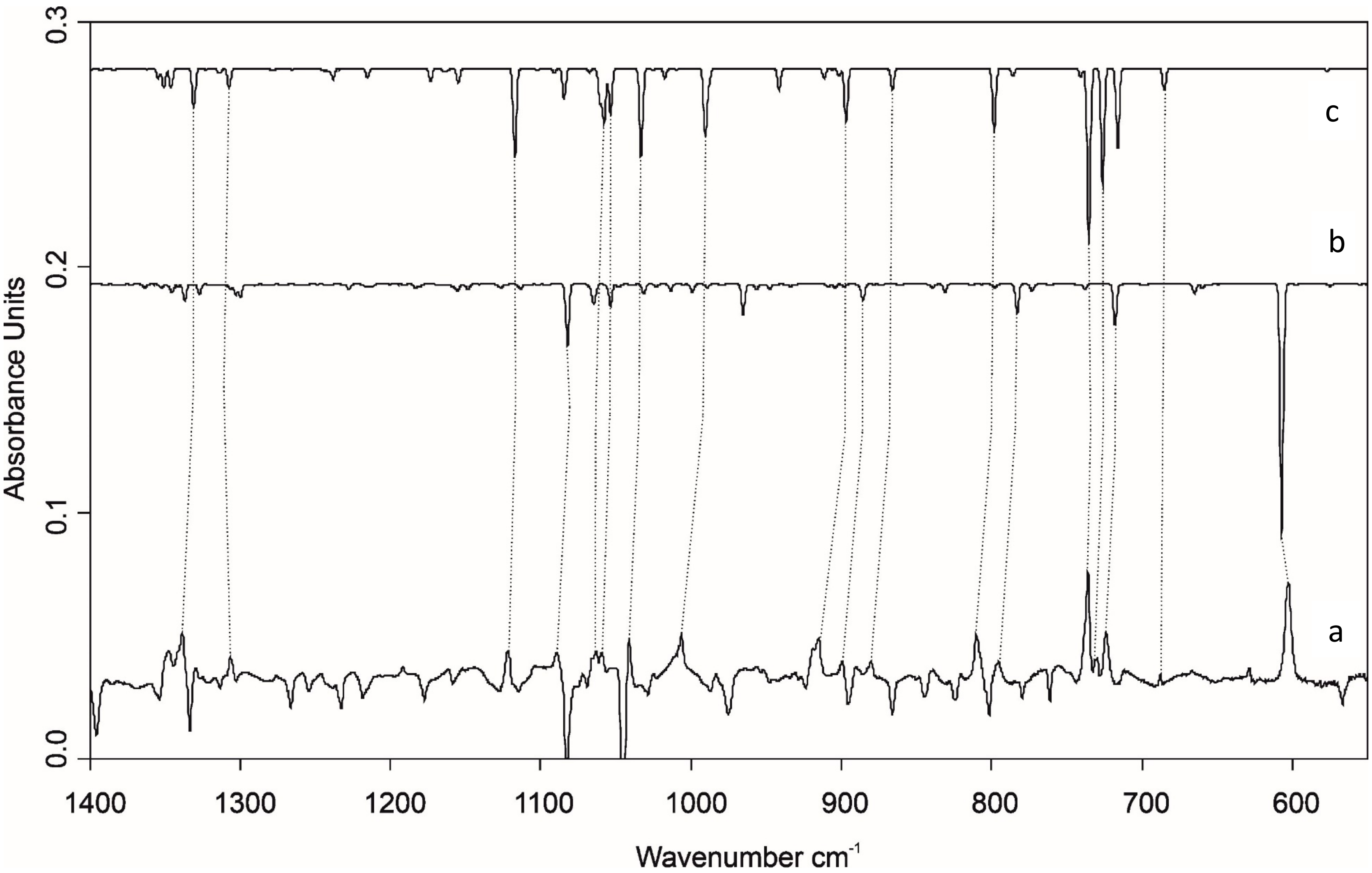

Figure S40: (a) IR difference spectra showing the photochemistry of d_2-**28** after irradiation with λ = 254 nm in argon at 3.5 K. Downward bands assigned to d_2-**28** disappear after 20 min irradiation. Upward bands assigned to d_2-**12** and d_2-**4** appear after 20 min irradiation. (b) IR spectrum of d_2-**12** computed at B3LYP/6-311++G(3df,2pd) (anharmonic). (c) IR spectrum of d_2-**4** computed at B3LYP/6-311++G(3df,2pd) (anharmonic).

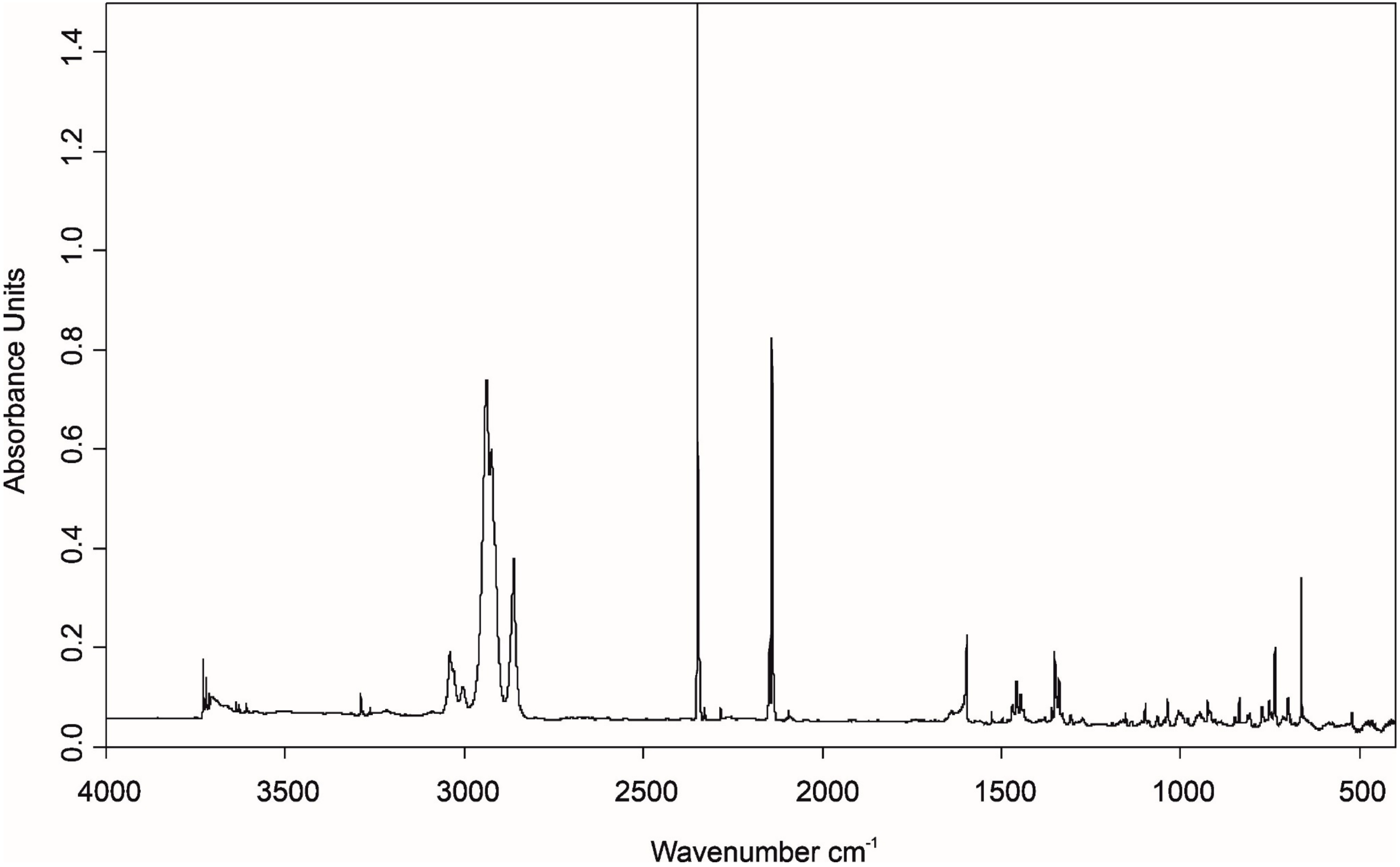

Figure S41: IR spectra showing the pyrolysis product of **13** with subsequent trapping in an argon matrix at 3.5 K.

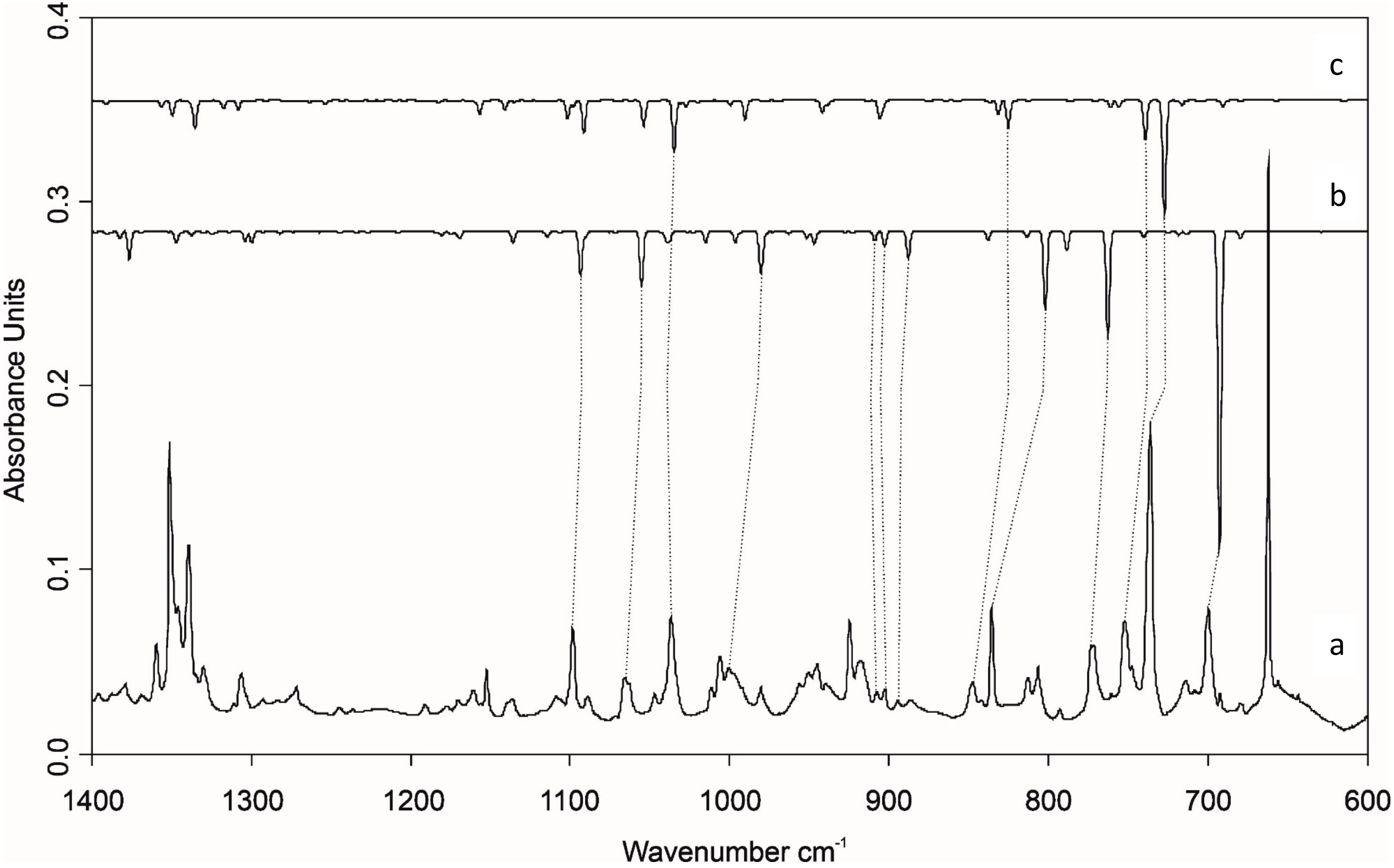

Figure S42: (a) IR spectra showing the pyrolysis product of **13** with subsequent trapping in an argon matrix at 3.5 K. (b) IR spectrum of **12** computed at B3LYP/6-311++G(3df,2pd) (anharmonic). (c) IR spectrum of **4** computed at B3LYP/6-311++G(3df,2pd) (anharmonic).

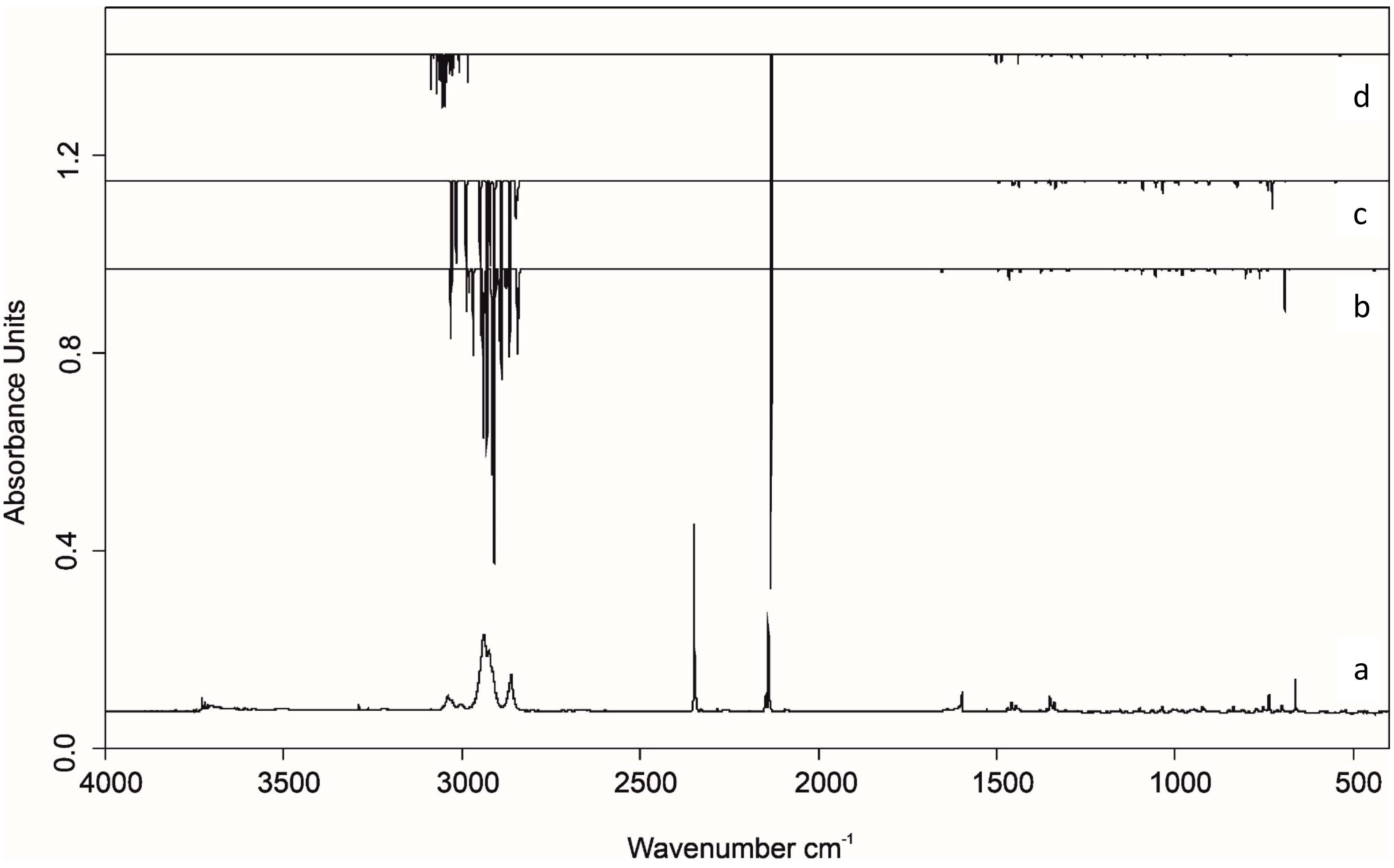

Figure S43: (a) IR spectra showing the pyrolysis of **13** with subsequent trapping in an argon matrix at 3.5 K. Bands assigned to adamantane diazo **28**, **12** and **4** appear after pyrolysis. (b) IR spectrum of 1**2** computed at UB3LYP/6-311++G(3df,2pd) (anharmonic). (c) IR spectrum of **4** computed at B3LYP/6-311++G(3df,2pd) (anharmonic). (d) IR spectrum of **28** computed at B3LYP/6-311++G(3df,2pd) (anharmonic).

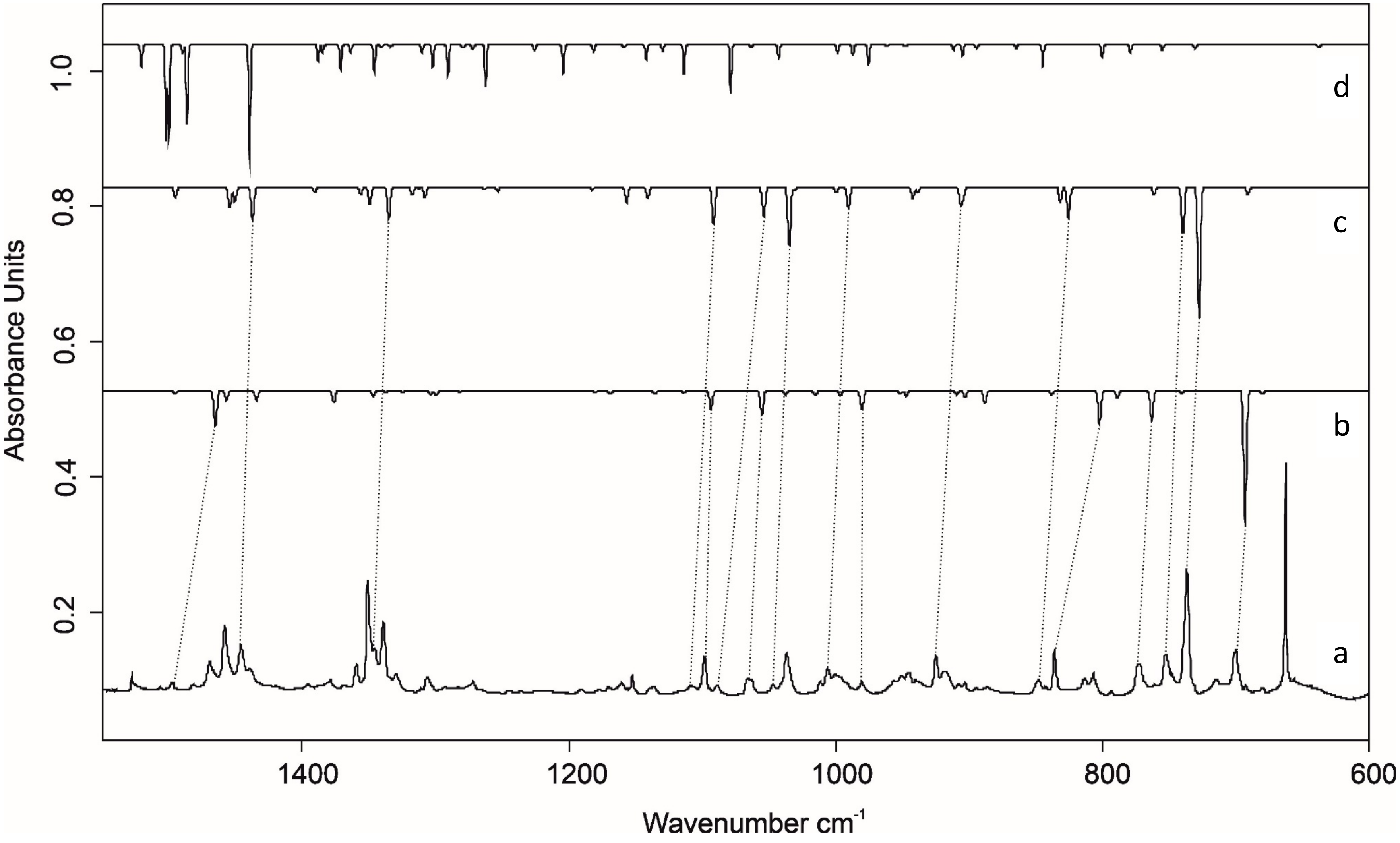

Figure S44: (a) IR spectra showing the pyrolysis of **13** with subsequent trapping in an argon matrix at 3.5 K. Bands assigned to adamantane diazo **28**, **12** and **4** appear after pyrolysis. The bands to compound **12** and **4** are assigned. (b) IR spectrum of 1**2** computed at UB3LYP/6-311++G(3df,2pd) (anharmonic). (c) IR spectrum of **4** computed at B3LYP/6-311++G(3df,2pd) (anharmonic). (d) IR spectrum of **28** computed at B3LYP/6-311++G(3df,2pd) (anharmonic).

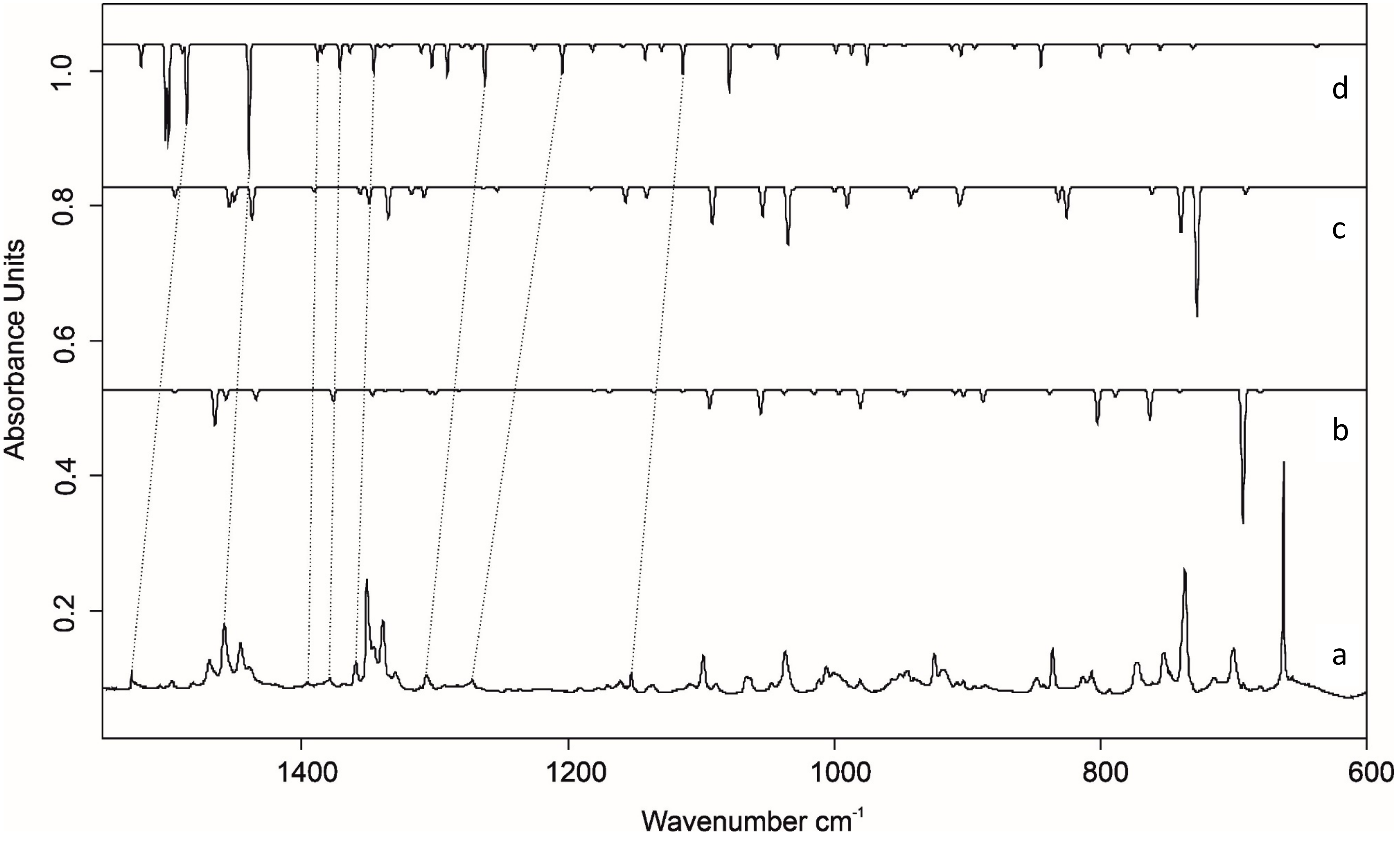

Figure S45: (a) IR spectra showing the pyrolysis of **13** with subsequent trapping in an argon matrix at 3.5 K. Bands assigned to adamantane diazo **28**, **12** and **4** appear after pyrolysis. The bands to compound **28** are assigned. (b) IR spectrum of 1**2** computed at UB3LYP/6-311++G(3df,2pd) (anharmonic). (c) IR spectrum of **4** computed at B3LYP/6-311++G(3df,2pd) (anharmonic). (d) IR spectrum of **28** computed at B3LYP/6-311++G(3df,2pd) (anharmonic).

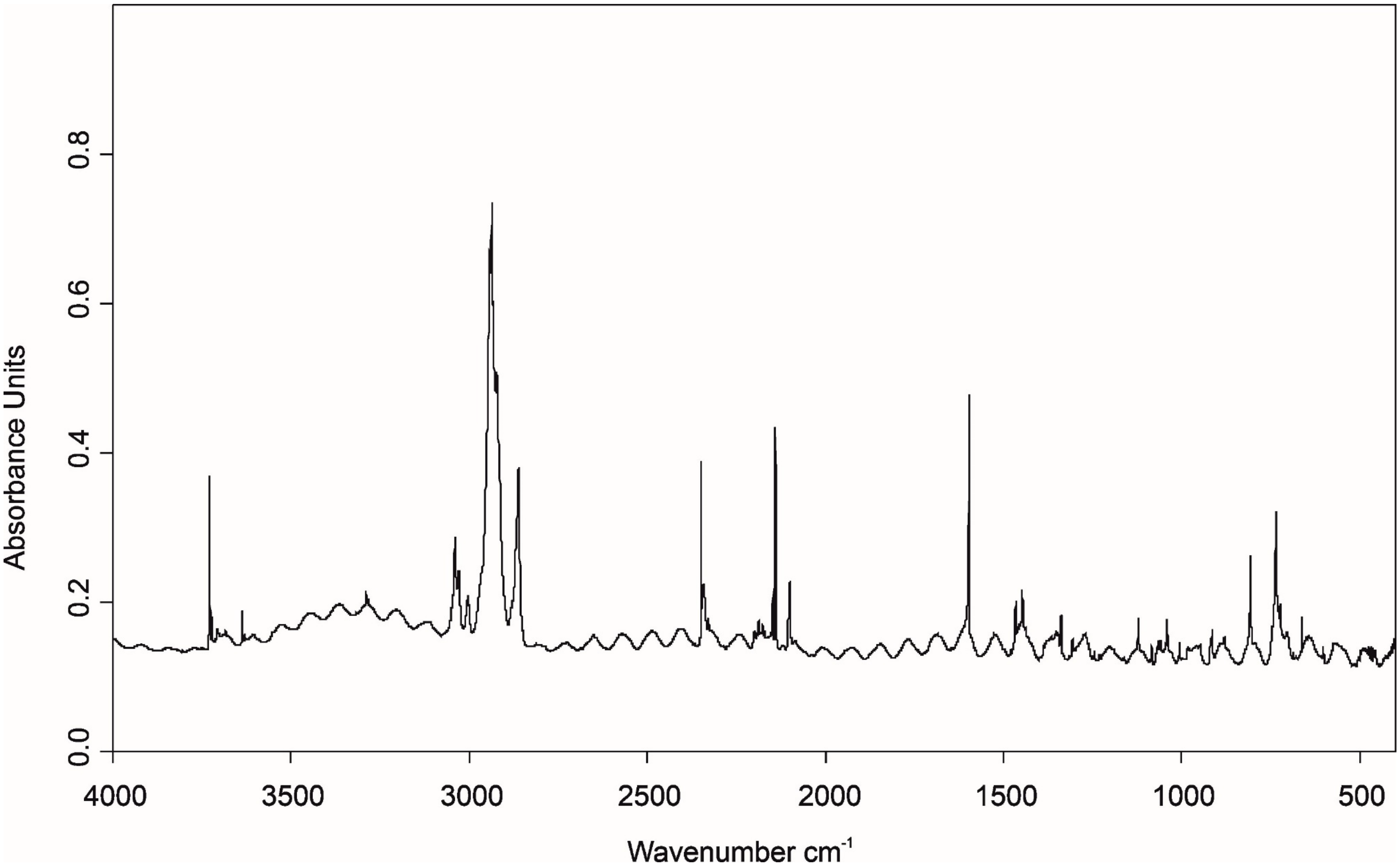

Figure S46: IR spectra showing the pyrolysis product of d_2-**13** with subsequent trapping in an argon matrix at 3.5 K.

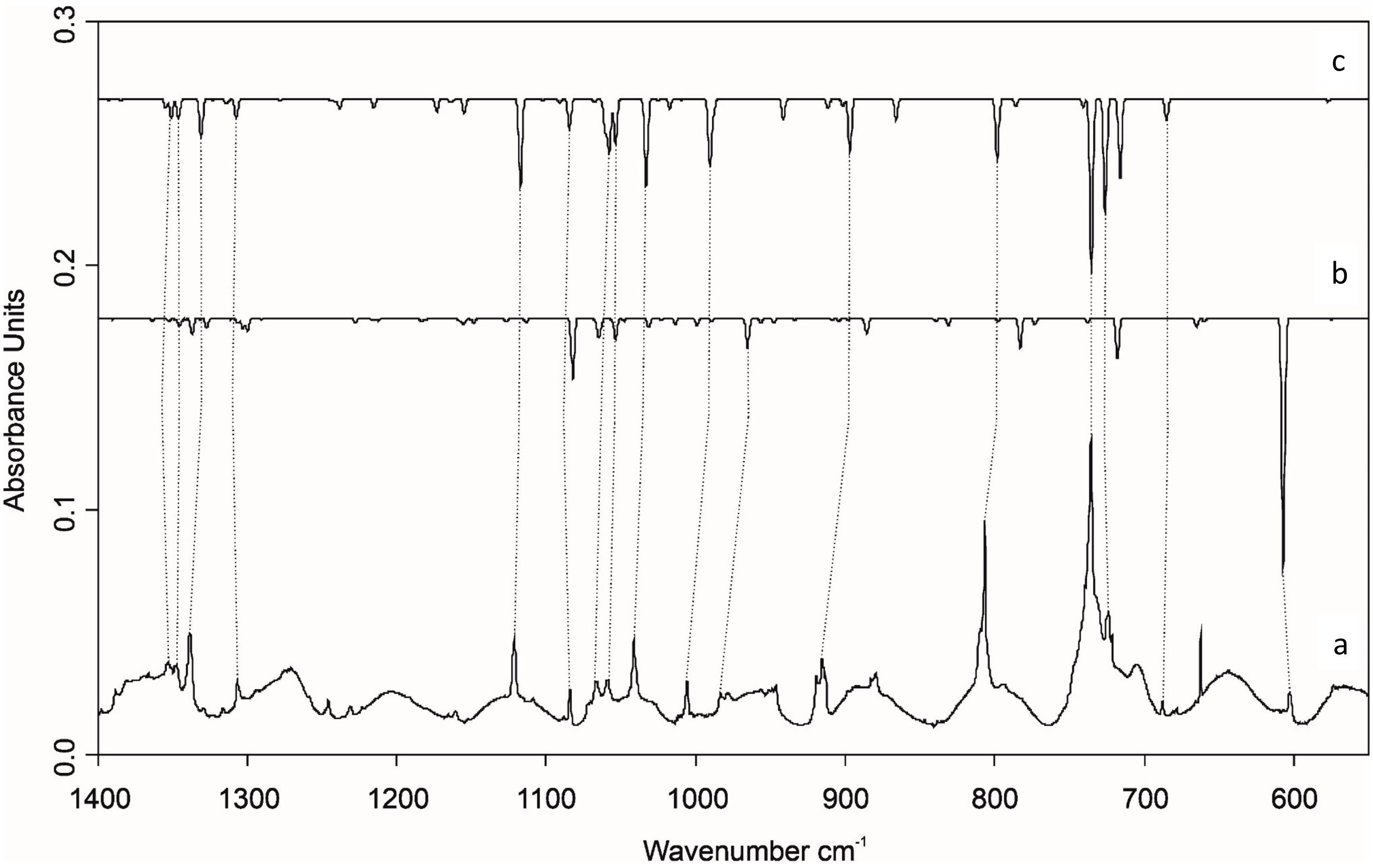

Figure S47: (a) IR spectra showing the pyrolysis product of d_2-**13** with subsequent trapping in an argon matrix at 3.5 K. (b) IR spectrum of d_2-**12** computed at B3LYP/6-311++G(3df,2pd) (anharmonic). (c) IR spectrum of d_2-**4** computed at B3LYP/6-311++G(3df,2pd) (anharmonic).

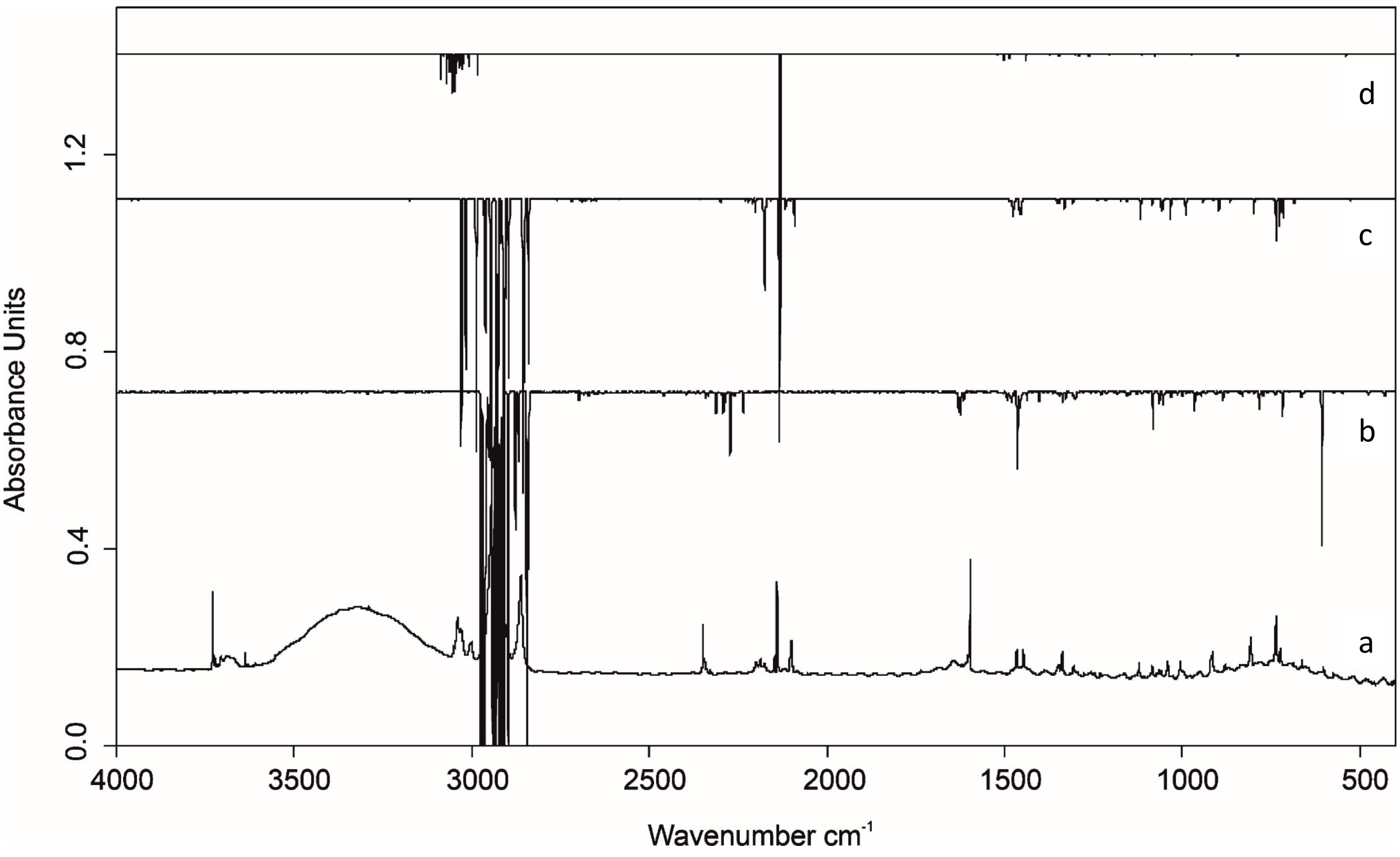

Figure S48: (a) IR spectra showing the pyrolysis of *d*$_2$-**13** with subsequent trapping in an argon matrix at 3.5 K.. Bands assigned to adamantane diazo *d*$_2$-**28**, *d*$_2$-**12** and *d*$_2$-**4** appear after pyrolysis. (b) IR spectrum of *d*$_2$-1**2** computed at UB3LYP/6-311++G(3df,2pd) (anharmonic). (c) IR spectrum of *d*$_2$-**4** computed at B3LYP/6-311++G(3df,2pd) (anharmonic). (d) IR spectrum of *d*$_2$-**28** computed at B3LYP/6-311++G(3df,2pd) (anharmonic).

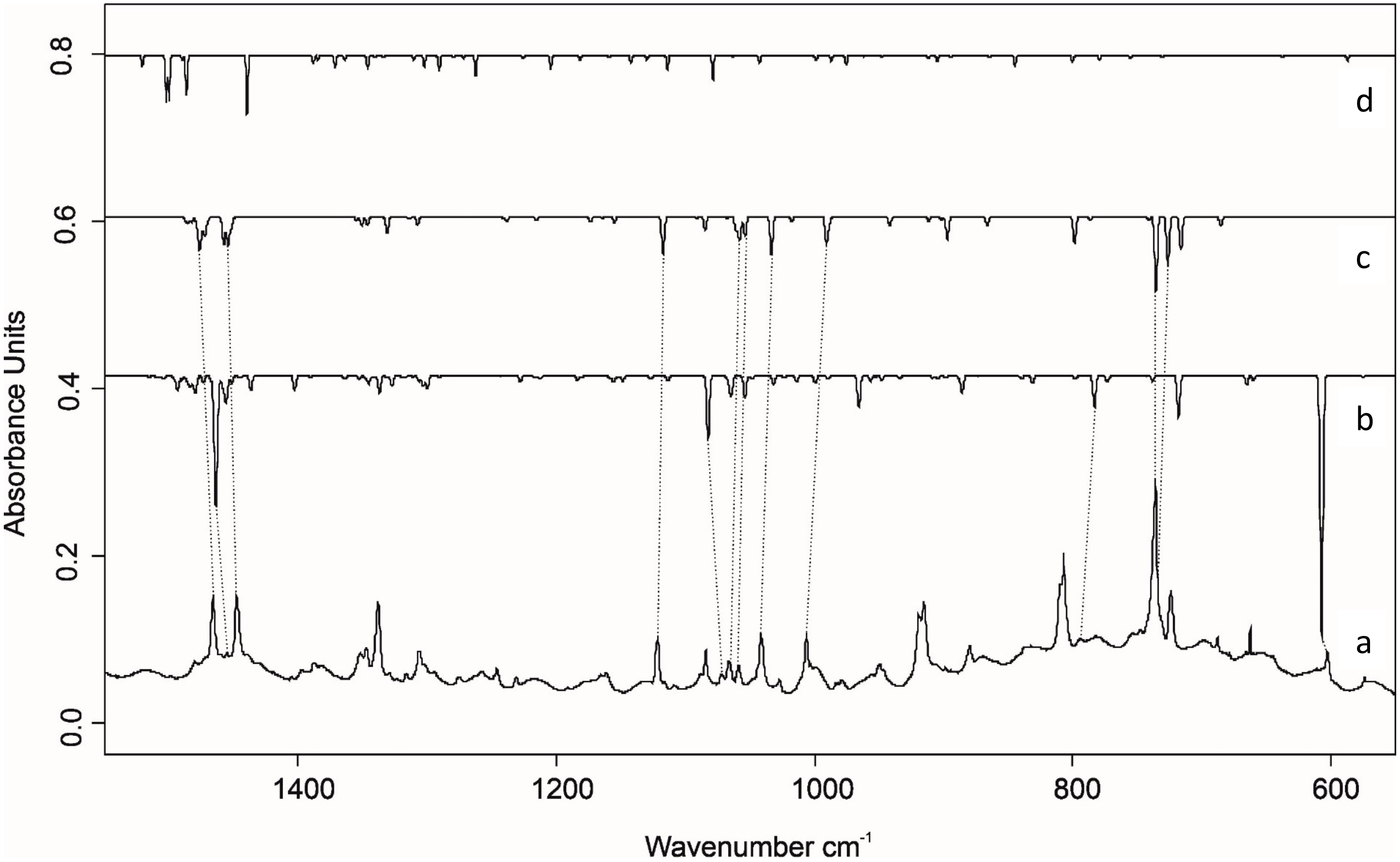

Figure S49: (a) IR spectra showing the pyrolysis of d_2-**13** with subsequent trapping in an argon matrix at 3.5 K.. Bands assigned to adamantane diazo d_2-**28**, d_2-**12** and d_2-**4** appear after pyrolysis. The bands to compound d_2-**12** and d_2-**4** are assigned. (b) IR spectrum of d_2-1**2** computed at UB3LYP/6-311++G(3df,2pd) (anharmonic). (c) IR spectrum of d_2-**4** computed at B3LYP/6-311++G(3df,2pd) (anharmonic). (d) IR spectrum of d_2-**28** computed at B3LYP/6-311++G(3df,2pd) (anharmonic).

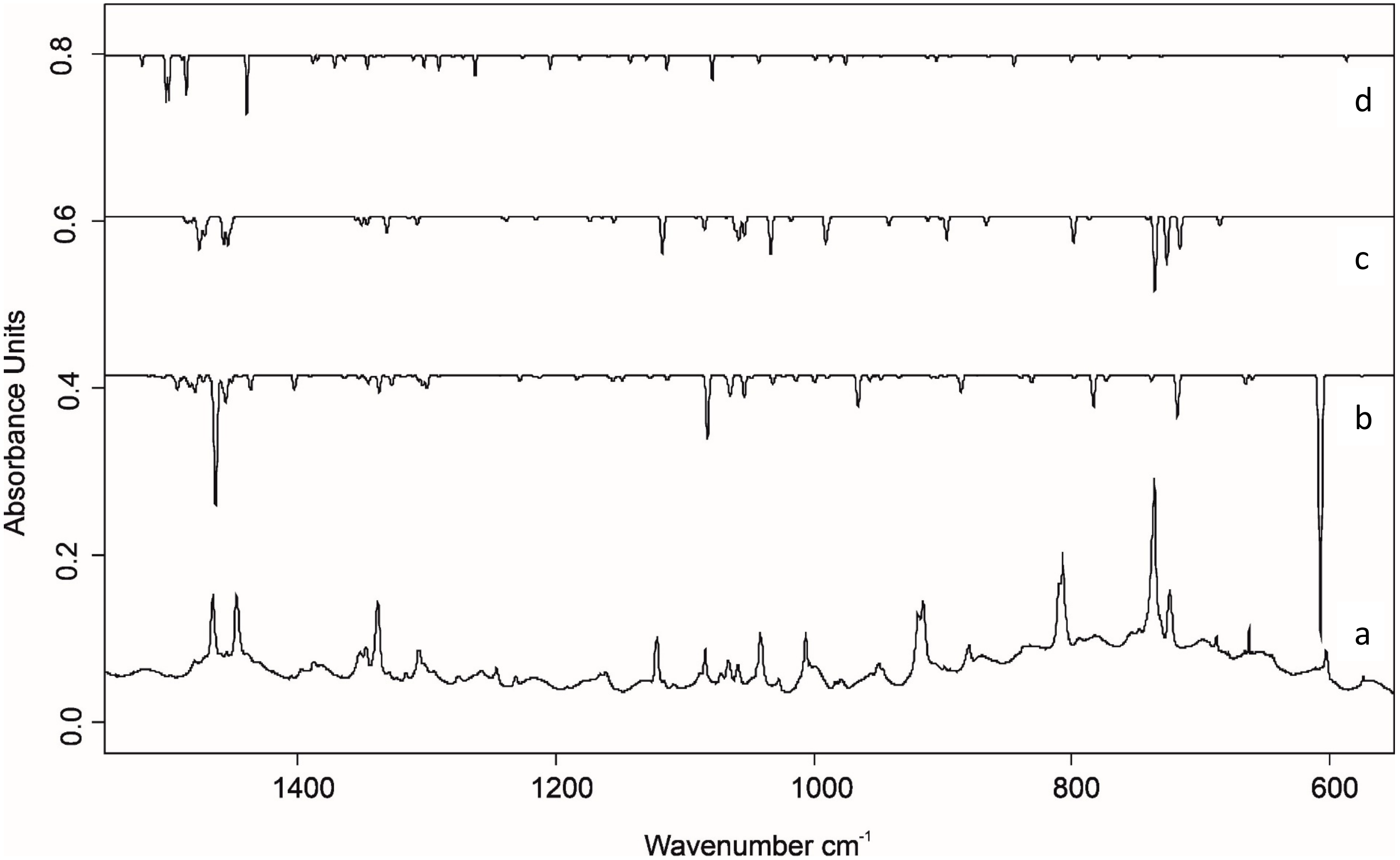

Figure S50: (a) IR spectra showing the pyrolysis of d_2-**13** with subsequent trapping in an argon matrix at 3.5 K.. Bands assigned to adamantane diazo d_2-**28**, d_2-**12** and d_2-**4** appear after pyrolysis. The bands to compound d_2-**28** cannot be assigned. (b) IR spectrum of d_2-1**2** computed at UB3LYP/6-311++G(3df,2pd) (anharmonic). (c) IR spectrum of d_2-**4** computed at B3LYP/6-311++G(3df,2pd) (anharmonic). (d) IR spectrum of d_2-**28** computed at B3LYP/6-311++G(3df,2pd) (anharmonic).

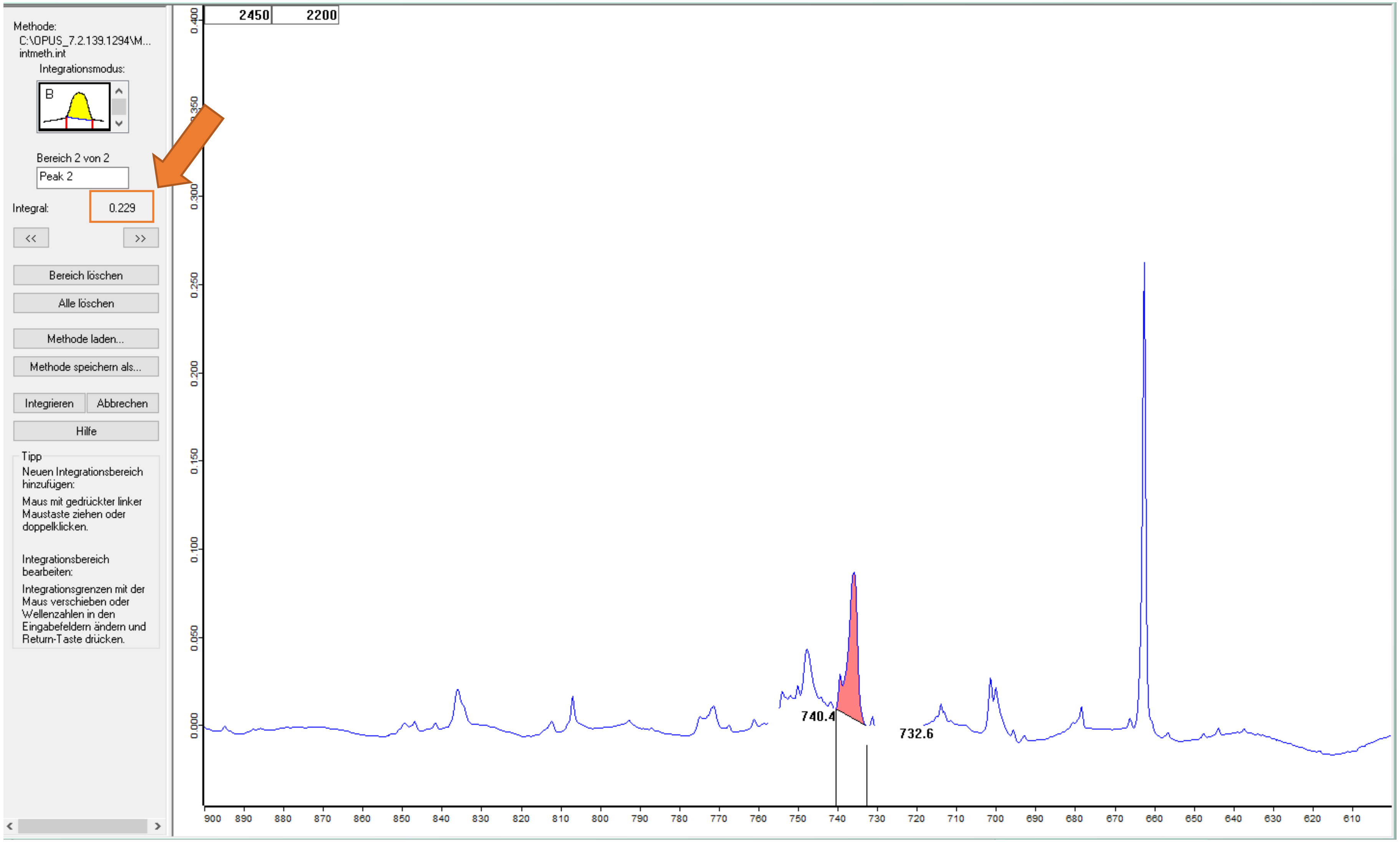

Figure S51: Screenshot of the IR spectrum showing the pyrolysis product of **13**, subsequently trapped in an argon matrix at 3.5 K. Integration of a peak from molecule **4**.

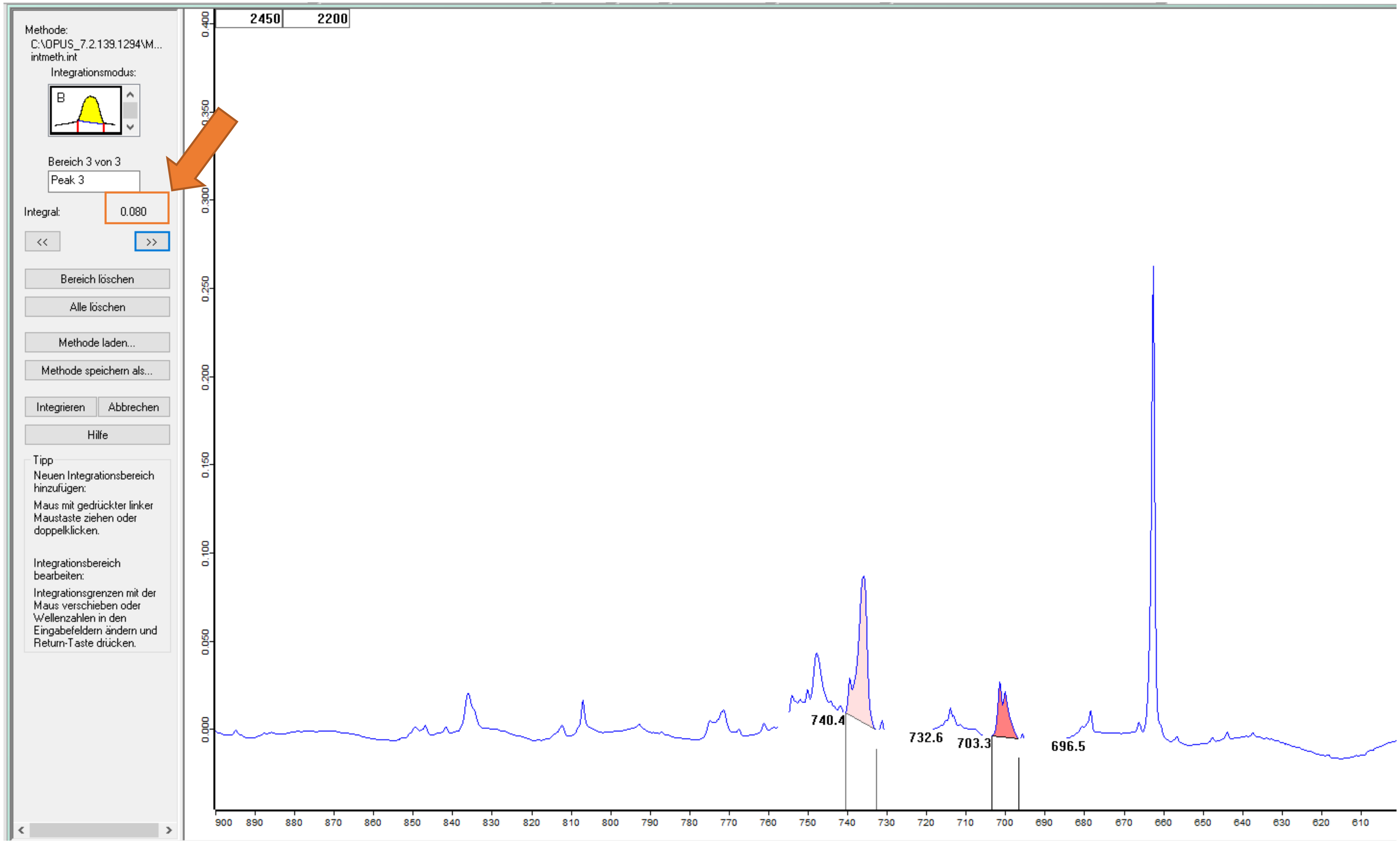

Figure S52: Screenshot of the IR spectrum showing the pyrolysis product of **13**, subsequently trapped in an argon matrix at 3.5 K. Integration of a peak from molecule **12**.

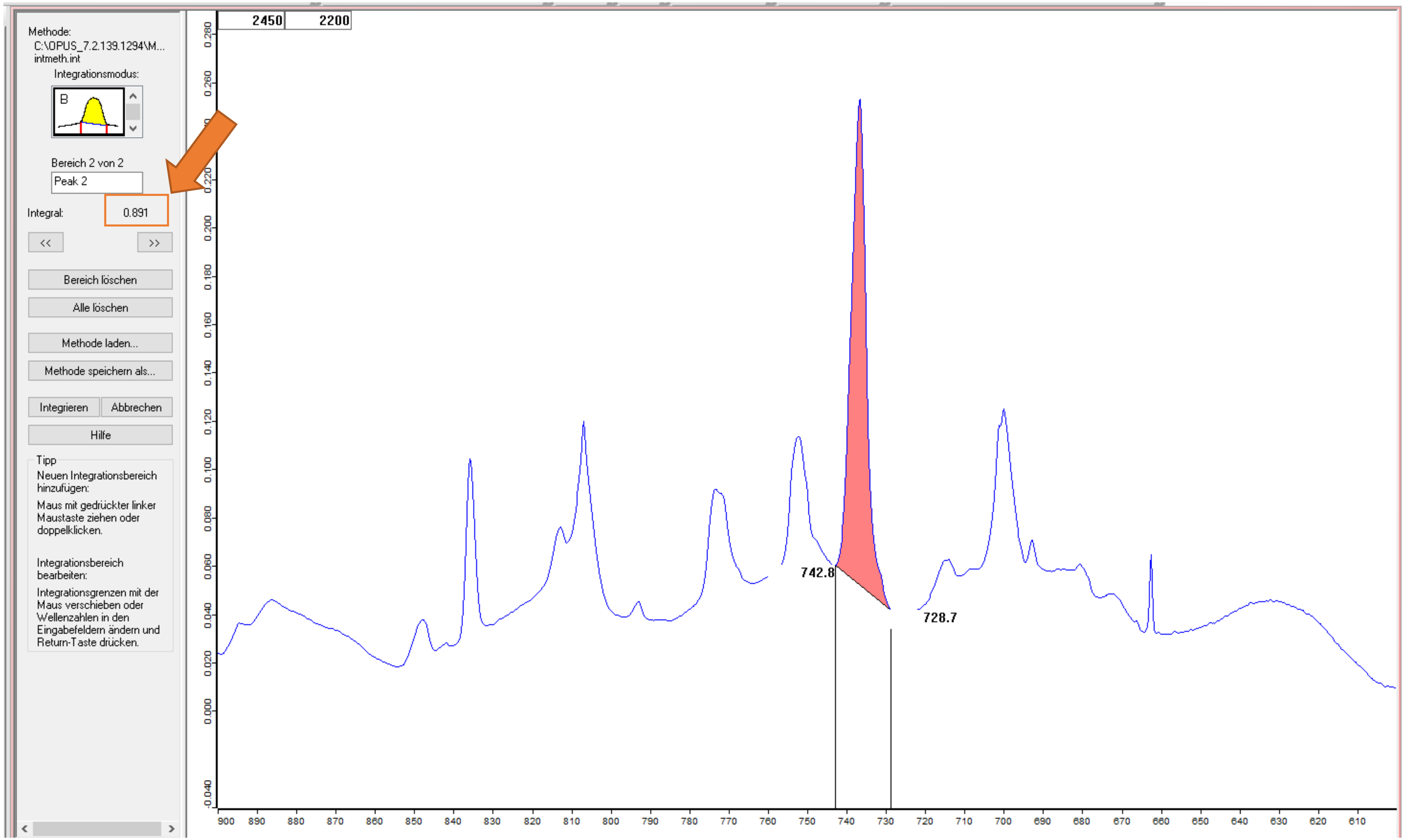

Figure S53: Screenshot of the IR spectrum showing the pyrolysis product of **13**, subsequently trapped in an argon matrix at 3.5 K. Integration of a peak from molecule **4**.

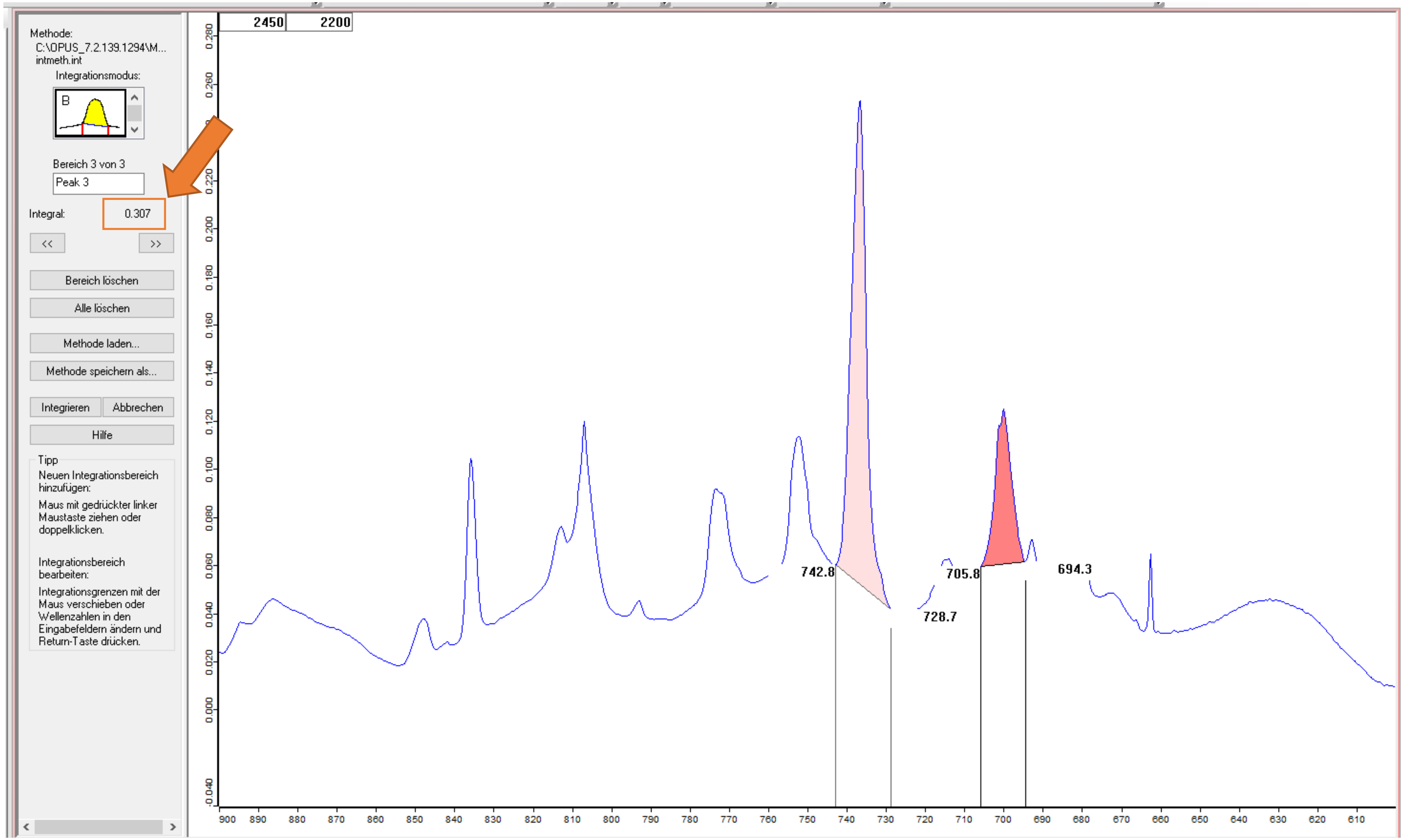

Figure S54: Screenshot of the IR spectrum showing the pyrolysis product of **13**, subsequently trapped in an argon matrix at 3.5 K. Integration of a peak from molecule **12**.

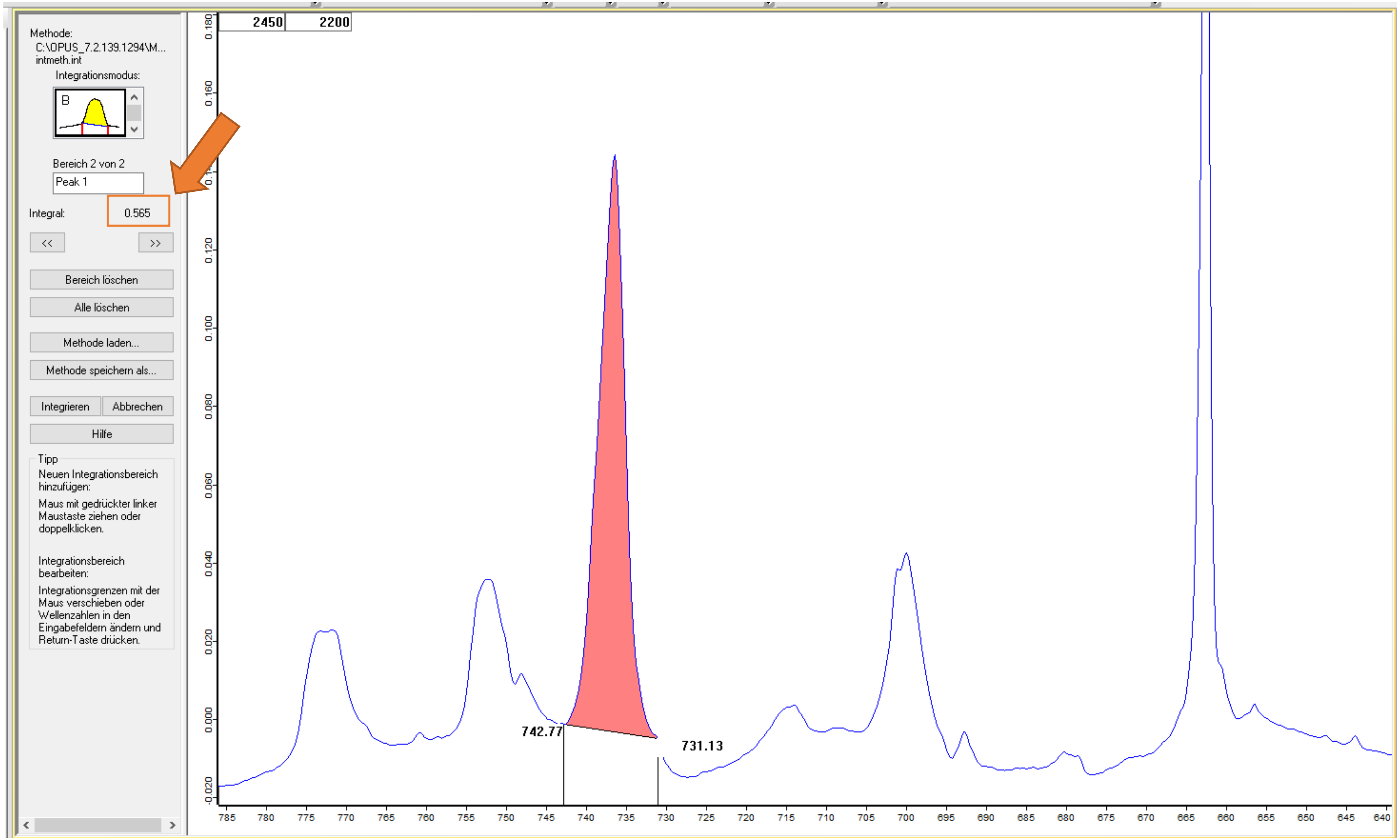

Figure S55: Screenshot of the IR spectrum showing the pyrolysis product of **13**, subsequently trapped in an argon matrix at 3.5 K. Integration of a peak from molecule **4**.

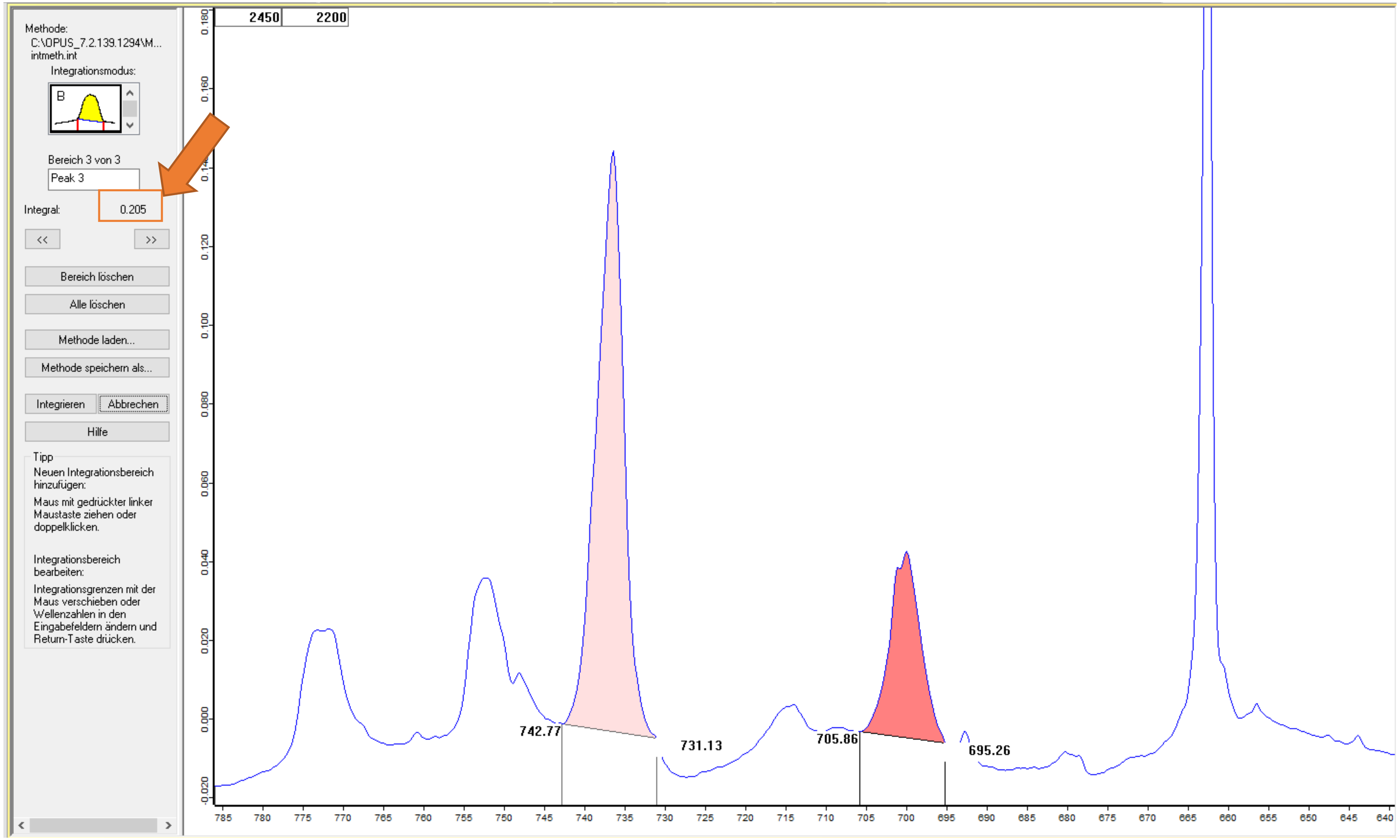

Figure S56: Screenshot of the IR spectrum showing the pyrolysis product of **13**, subsequently trapped in an argon matrix at 3.5 K. Integration of a peak from molecule **12**.

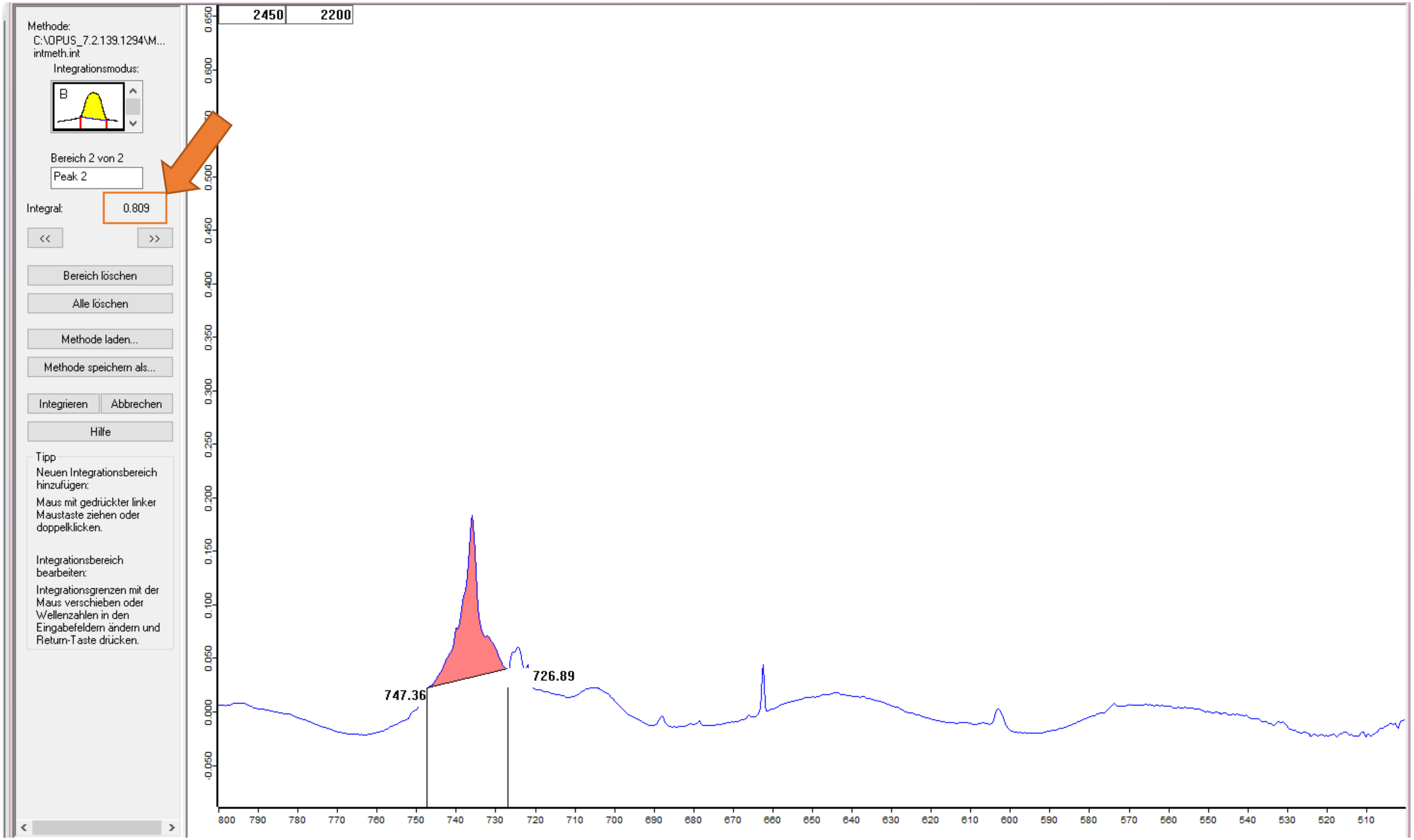

Figure S57: Screenshot of the IR spectrum showing the pyrolysis product of d_2-**13**, subsequently trapped in an argon matrix at 3.5 K. Integration of a peak from molecule d_2-**4**.

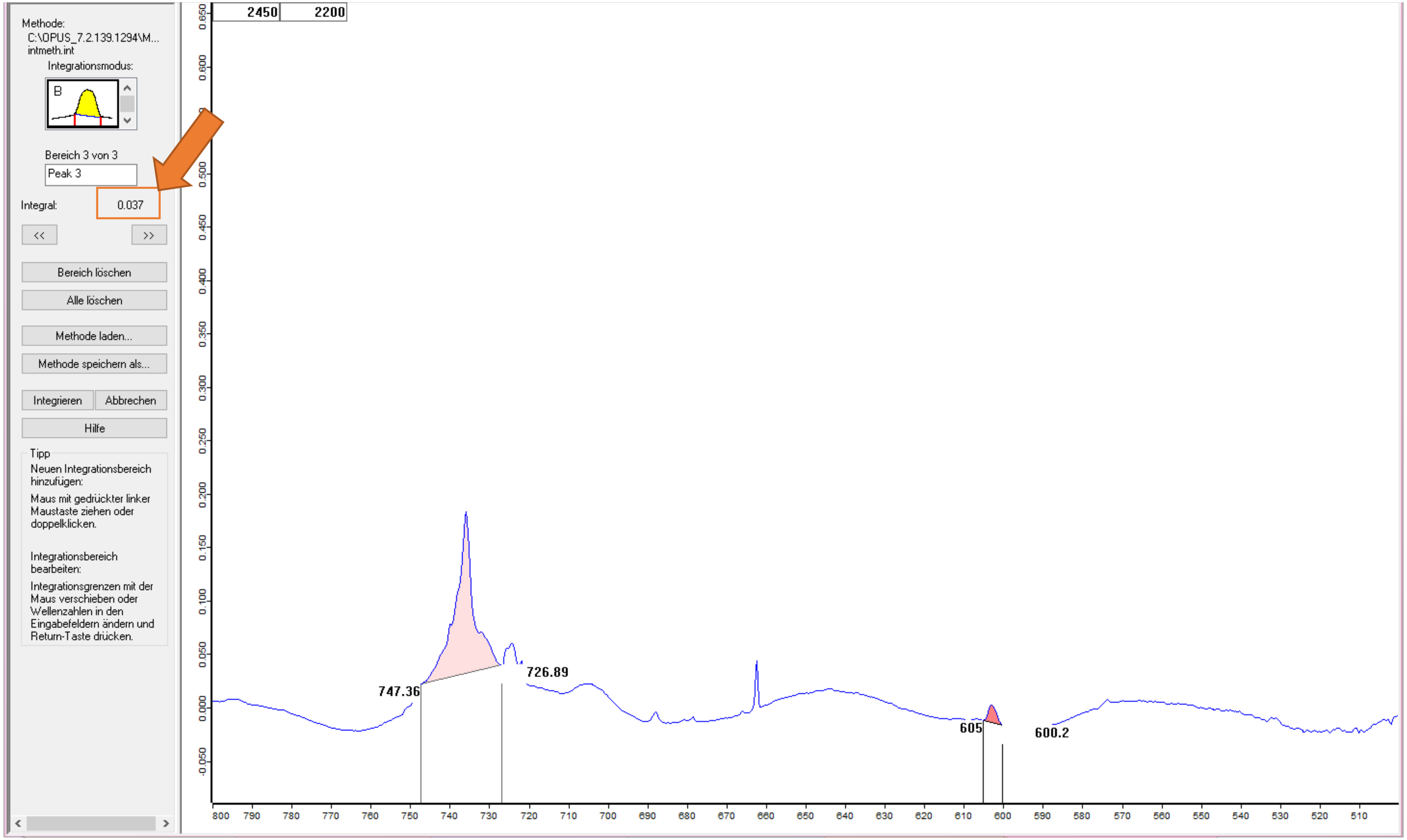

Figure S58: Screenshot of the IR spectrum showing the pyrolysis product of d_2-**13**, subsequently trapped in an argon matrix at 3.5 K. Integration of a peak from molecule d_2-**12**.

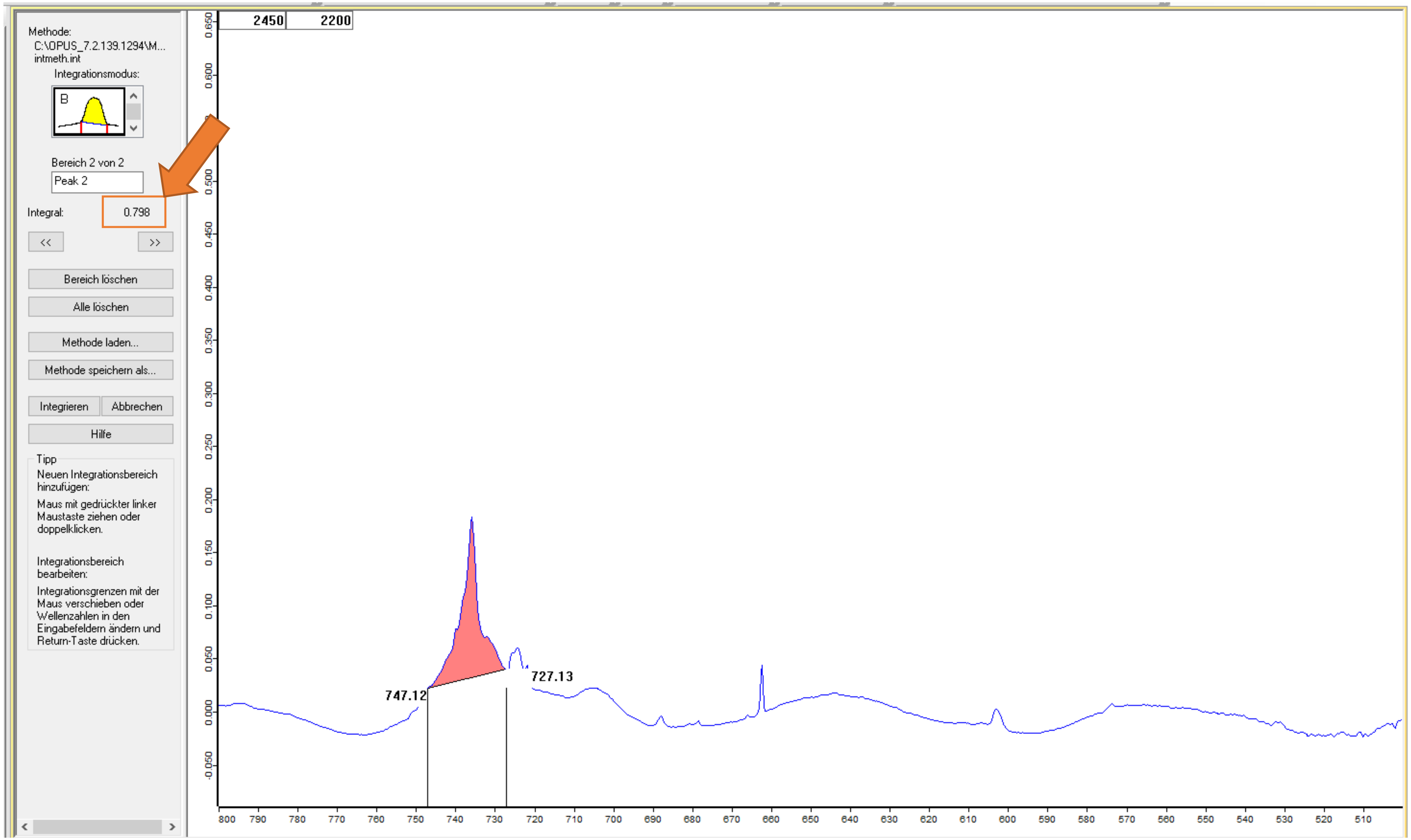

Figure 59: Screenshot of the IR spectrum showing the pyrolysis product of d_2-**13**, subsequently trapped in an argon matrix at 3.5 K. Integration of a peak from molecule d_2-**4**.

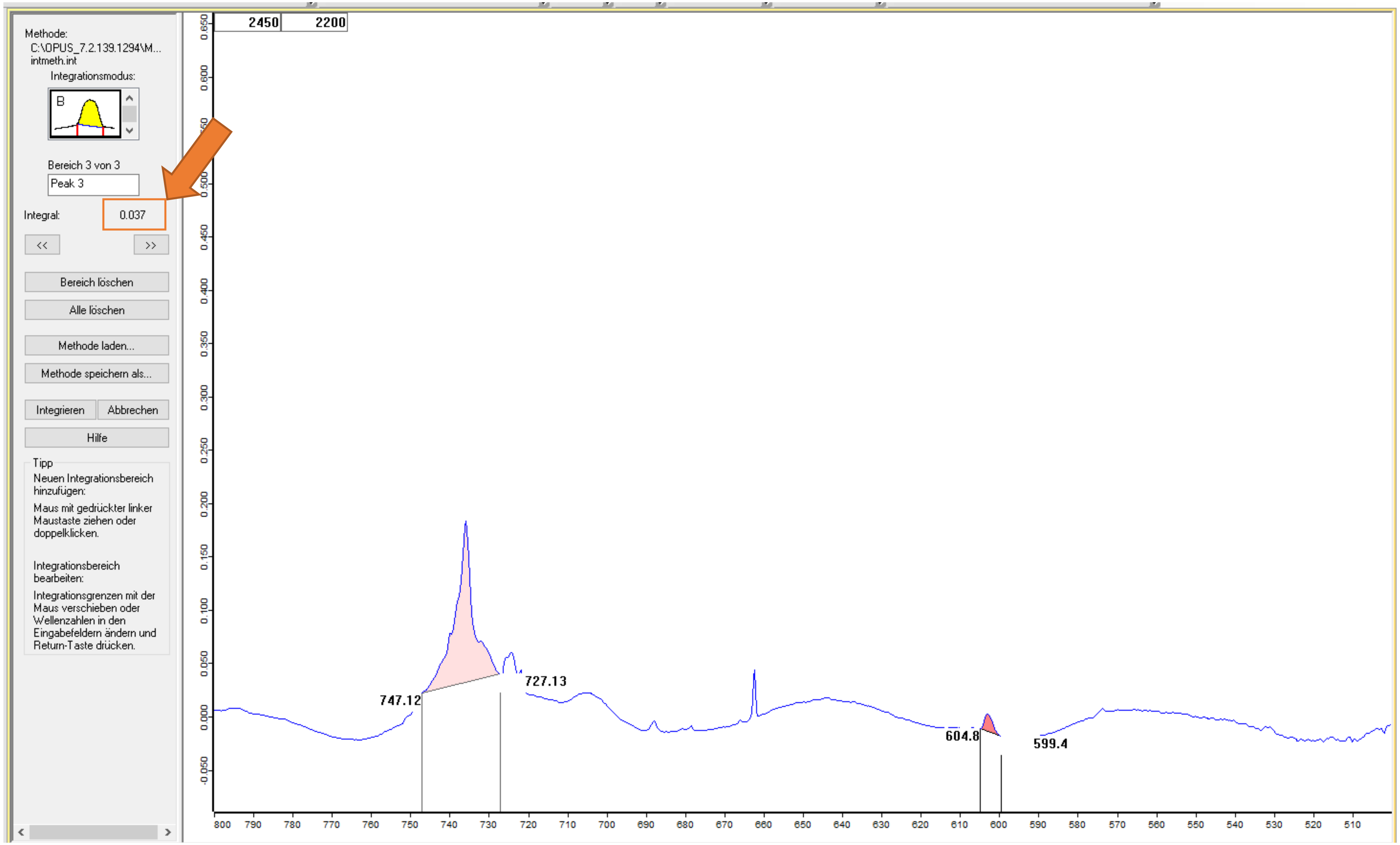

Figure S60: Screenshot of the IR spectrum showing the pyrolysis product of *d*$_2$-**13**, subsequently trapped in an argon matrix at 3.5 K. Integration of a peak from molecule *d*$_2$-**12**.

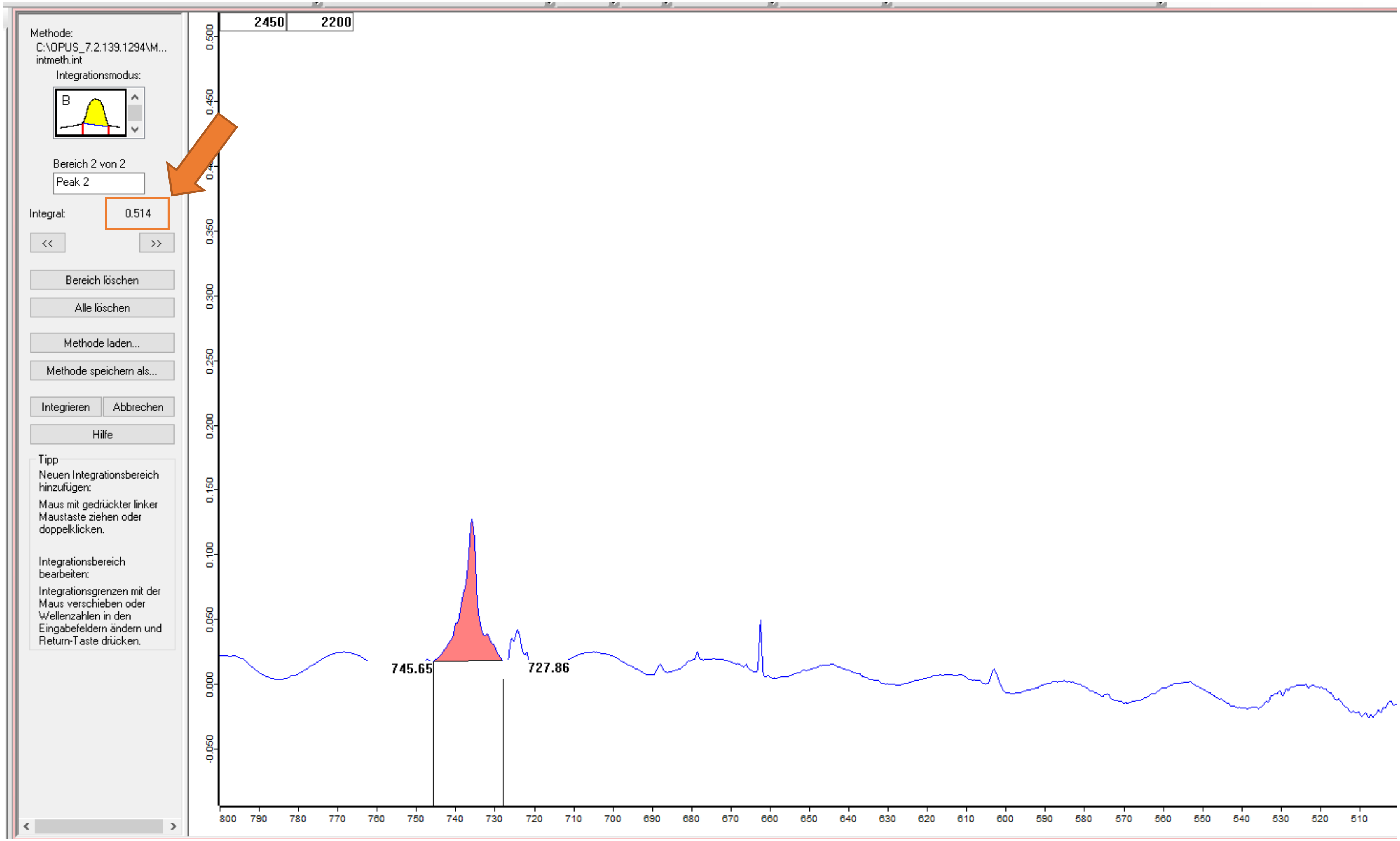

Figure S61: Screenshot of the IR spectrum showing the pyrolysis product of *d*$_2$-**13**, subsequently trapped in an argon matrix at 3.5 K. Integration of a peak from molecule *d*$_2$-**4**.

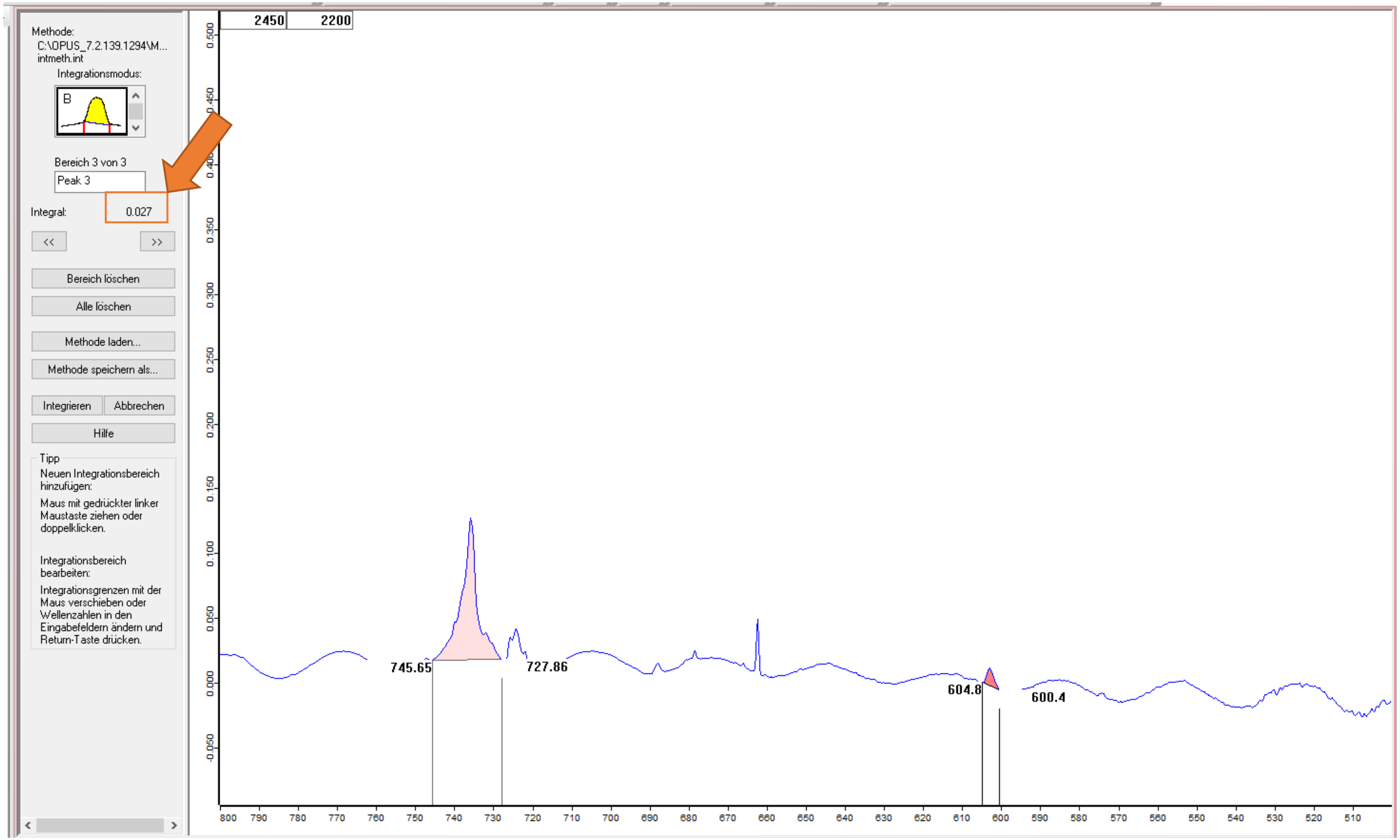

Figure S62: Screenshot of the IR spectrum showing the pyrolysis product of d_2-**13**, subsequently trapped in an argon matrix at 3.5 K. Integration of a peak from molecule d_2-**12**.

Table S1: Experimental (Ar matrix, 3.5 K) and computed IR frequencies of **1** band origins in cm^{-1}, computed intensities ($km\ mol^{-1}$) in parantheses.

Mode	**1** Computed[a]	**1** N_2 3.5 K[b]	Assignement
1	3056 (38.8)	not definitively identifiable	Antisymmetric stretching C–H (CH_2)
2	3056 (71.4)	not definitively identifiable	Antisymmetric stretching C–H (CH_2)
3	3054 (32.6)	not definitively identifiable	Antisymmetric stretching C–H (CH_2)
5	3043 (41.0)	not definitively identifiable	Antisymmetric stretching C–H (CH_2)
6	3035 (32.6)	not definitively identifiable	Symmetric stretching C–H (CH_2)
7	3026 (94.4)	not definitively identifiable	Symmetric stretching C–H (CH_2)
8	3026 (169.3)	not definitively identifiable	Symmetric stretching C–H (CH_2)
9	3024 (57.3)	not definitively identifiable	Symmetric stretching C–H (CH)
11	3010 (22.8)	not definitively identifiable	Symmetric stretching C–H (CH_2)
12	3010 (4.5)	not definitively identifiable	Symmetric stretching C–H (CH_2)
14	3006 (13.6)	not definitively identifiable	Symmetric stretching C–H (CH_2)
15	1516 (2.7)	not definitively identifiable	Scissoring C–H (CH_2)
16	1499 (9.6)	1470 (m)	Scissoring C–H (CH_2)
17	1495 (10.2)	1470 (m)	Scissoring C–H (CH_2)
18	1490 (3.5)	/	Scissoring C–H (CH_2)
24	1368 (1.0)	1357 (w)	Wagging C–H
27	1339 (0.7)	1313 (w)	Wagging C–H (CH)

30	1294 (2.8)	/	Twisting C–H (CH_2)
33	1238 (3.2)	1235 (m)	Twisting C–H (CH)
37	1113 (0.9)	1111 (w)	Wagging C–H
40	1046 (7.1)	1043 (m)	Scissoring C–C
41	1042 (4.5)	1038 (w)	Rocking C–C
43	978 (1.4)	982 (w)	Scissoring C–C
44	957 (1.5)	951 (m)	Scissoring C–C
45	932 (1.3)	927 (m)	Wagging C–C
49	886 (3.5)	870 (w)	Wagging C–C
51	784 (2.2)	775 (w)	Twisting C–C
54	643 (7.8)	602 (s)	Twisting C–C

[a]B3LYP/6-311++G(3df,2pd), anharmonic, unscaled frequencies, intensities (in parentheses) in km mol^{-1}. [b]Experiment: argon matrix, 3.5 K; approximate relative intensities (w: weak, m: medium, s: strong).

Table S2: Experimental (Ar matrix, 3.5 K) and computed IR frequencies of **2** band origins in cm^{-1}, computed intensities (km mol^{-1}) in parantheses.

Mode	**2** Computed[a]	**2** N_2 3.5 K[b]	Assignment
1	3006 (11.4)	not definitively identifiable	Symmetric stretching C–H (CH)
2	2982 (45.3)	not definitively identifiable	Symmetric stretching C–H
3	2974 (72.6)	not definitively identifiable	Symmetric stretching C–H (CH)
4	2964 (10.3)	not definitively identifiable	Symmetric stretching C–H (CH)

5	2951 (64.9)	not definitively identifiable	Symmetric stretching C–H (CH)
6	2942 (66.9)	not definitively identifiable	Antisymmetric stretching C–H (CH_2)
7	2935 (84.7)	not definitively identifiable	Antisymmetric stretching C–H (CH_2)
8	2925 (8.4)	not definitively identifiable	Antisymmetric stretching C–H (CH_2)
9	2911 (18.1)	not definitively identifiable	Antisymmetric stretching C–H (CH_2)
10	2865 (28.9)	not definitively identifiable	Antisymmetric stretching C–H (CH)
11	2847 (26.5)	not definitively identifiable	Antisymmetric stretching C–H (CH_2)
12	1463 (1.0)	1458 (m)	Scissoring C–H (CH_2)
13	1454 (1.3)	1446 (m)	Scissoring C–H (CH_2)
14	1311 (0.8)	1303 (m)	Scissoring C–H (CH2)
15	1303 (3.1)	1298 (m)	Scissoring C–H (CH_2)
16	1292 (1.0)	1291 (w)	Rocking C–H (CH_2)
17	1271 (1.2)	1272 (w)	Wagging C–H (CH_2)
18	1217 (3.3)	1223 (m)	Wagging C–H (CH)
19	1212 (0.7)	1211 (m)	Twisting C–H
20	1201 (0.6)	1198 (w)	Wagging C–H (CH_2)
21	1102 (2.1)	1104 (w)	Twisting C–H (CH)
22	1094 (7.1)	1096 (s)	Twisting C–H (CH_2)
23	1088 (2.4)	/	Twisting C–H (CH_2)
24	1049 (3.6)	1054 (m)	Twisting C–H (CH_2)
25	1030 (2.4)	1023 (m)	Wagging C–H
26	1008 (2.6)	1013 (w)	Rocking C–C
27	980 (1.6)	984 (w)	Rocking C–C
28	962 (0.6)	969 (w)	Scissoring C–C
29	938 (1.7)	951 (w)	Rocking C–C
30	906 (2.9)	909 (w)	Scissoring C–C

31	899 (2.2)	893 (s)	Scissoring C–C
32	883 (1.1)	860 (s)	Twisting C–C
33	823 (1.1)	/	Wagging C–C
34	810 (1.1)	/	Twisting C–C
35	749 (1.7)	/	Wagging C–C
36	640 (8.9)	/	Twisting C–C

[a]B3LYP/6-311++G(3df,2pd), anharmonic, unscaled frequencies, intensities (in parentheses) in km mol^{-1}. [b]Experiment: argon matrix, 3.5 K; approximate relative intensities (w: weak, m: medium, s: strong).

Table S3: Integrals, ratio, and mean of the undeuterated compounds.

	**Figure S39-S40**	**Figure S41-S42**	**Figure S43-S44**
**4**	0.229	0.891	0.565
**12**	0.080	0.307	0.205
**Ratio**	0.651	0.655	0.637
**Mean**		0.648	

Table S4: Integrals, ratio, and mean of the deuterated compounds.

	**Figure S45-S46**	**Figure S47-S48**	**Figure S49-S50**
***d_2*-4**	0.809	0.798	0.514
***d_2*-12**	0.037	0.037	0.027
**Ratio**	0.954	0.954	0.947
**Mean**		0.952	

Table S5: Determination of the vibrational frequency scaling factors for the deuterated and undeuterated compounds.

	Wavenumber	Intensity
**4**	727.395	8.8313
***d*$_2$-4**	726.107	4.2802
**Factor (4)**	2.063	
**12**	692.806	22.987
***d*$_2$-12**	615.781	18.357
**Factor (12)**	1.252	

Table S6: Normalized experimental intensities using scaling factor.

**4 (with correction)**	1.669	1.647	1.061
**12 (with correction)**	0.046	0.046	0.033
**Ratio (with correction)**	0.972	0.972	0.968
**Mean (with correction)**		0.971	

UV/Vis Spectral Data

Table S7: TD-B3LYP/6-311++G(3df,2pd) computed vertical excitation energies of adamantane diazirine (**5**).

Excitation Energy λ / nm	Oscillator Strength (*f*)
369.88	0.0007
234.59	0.0029
200.57	0.0019

Table S8: TD-B3LYP/6-311++G(3df,2pd) computed vertical excitation energies of diazoadamantane (**26**).

Excitation Energy λ / nm	Oscillator Strength (*f*)
295.64	0.0014
256.37	0.0217
233.00	0.0023
227.41	0.0098
225.25	0.0053
221.88	0.0065
212.50	0.1004
204.25	0.0059

Table S9: TD-B3LYP/6-311++G(3df,2pd) computed vertical excitation energies of adamantylidene (**1**).

Excitation Energy λ / nm	Oscillator Strength (*f*)
1083.82	0.0030
674.31	0.0011
296.77	0.0038
291.06	0.0058
258.88	0.0117
254.50	0.0063
251.39	0.0038
251.03	0.0013
246.71	0.0070

Table S10: TD-B3LYP/6-311++G(3df,2pd) computed vertical excitation energies of 2,4-dehydroadamantane (**4**).

Excitation Energy λ / nm	Oscillator Strength (*f*)
210.51	0.0076

Table S11: TD-B3LYP/6-311++G(3df,2pd) computed vertical excitation energies of adamantane alkene (**10**).

Excitation Energy λ / nm	Oscillator Strength (*f*)
339.62	0.0442
293.39	0.0148
261.6	0.0199
259.25	0.0206
250.98	0.0022
236.1	0.0035
233.77	0.0107
228.32	0.0054
224.94	0.0055
220.84	0.0119
209.63	0.0035
204.01	0.0156

Table S12: TD-B3LYP/6-311++G(3df,2pd) computed vertical excitation energies of pentacycloundecane diazirine (**6**).

Excitation Energy λ / nm	Oscillator Strength (*f*)
366.57	0.0009
240.64	0.0019
212.18	0.0014

Table S13: TD-B3LYP/6-311++G(3df,2pd) computed vertical excitation energies of pentacycloundecane diazo (**27**).

Excitation Energy λ / nm	Oscillator Strength (*f*)
258.31	0.0174
254.53	0.0173
252.75	0.0027
231.48	0.0379
226.98	0.0076
225.82	0.0095
223.26	0.0051
220.11	0.0128
214.9	0.0196

Table S14: TD-B3LYP/6-311++G(3df,2pd) computed vertical excitation energies of pentacycloundecane-carbene (**2**).

Excitation Energy *λ* / nm	Oscillator Strength (*f*)
974.94	0.0043
727.79	0.0010
274.78	0.0018
273.51	0.0010
251.71	0.0012
250.38	0.0046
248.18	0.0037
247.41	0.0038
244.18	0.0017

Table S15: TD-B3LYP/6-311++G(3df,2pd) computed vertical excitation energies of churchane (**11**).

Excitation Energy *λ* / nm	Oscillator Strength (*f*)
/	/

Table S16: TD-B3LYP/6-311++G(3df,2pd) computed vertical excitation energies of homohypostrophene (**9**).

Excitation Energy *λ* / nm	Oscillator Strength (*f*)
233.45	0.0279
221.66	0.0211
215.15	0.0092
210.17	0.0098
209.38	0.0097

Table S17: TD-B3LYP/6-311++G(3df,2pd) computed vertical excitation energies of protoadamantane diazirine (**13**).

Excitation Energy *λ* / nm	Oscillator Strength (*f*)
225.54	0.0036
215.34	0.0021
210.58	0.0016

Table S18: TD-B3LYP/6-311++G(3df,2pd) computed vertical excitation energies of protoadamantane diazo (**28**).

Excitation Energy *λ* / nm	Oscillator Strength (*f*)
295.5	0.0007
260.55	0.0077
256	0.0070
253.15	0.0028
235.05	0.0305
231.06	0.0378
228.98	0.0039
226.11	0.0112
221.5	0.0063
210.33	0.0878

Table S19: TD-B3LYP/6-311++G(3df,2pd) computed vertical excitation energies of protoadamantylidene (**3**).

Excitation Energy *λ* / nm	Oscillator Strength (*f*)
531.88	0.0033
308.42	0.0018
271.5	0.0045
267.77	0.0037
259.35	0.0374
238.76	0.0019
233.55	0.0030
223.82	0.0038
207.31	0.0065

Table S20: TD-B3LYP/6-311++G(3df,2pd) computed vertical excitation energies of protoadamantane dehydro (**4**).

Excitation Energy *λ* / nm	Oscillator Strength (*f*)
210.48	0.0076

Table S21: TD-B3LYP/6-311++G(3df,2pd) computed vertical excitation energies of protoadamantane alkene (**12**).

Excitation Energy *λ* / nm	Oscillator Strength (*f*)
228.43	0.0135
208.54	0.0269
206.9	0.0151
202.41	0.0609
201.45	0.0082

Table S22: TD-B3LYP/6-311++G(3df,2pd) computed vertical excitation energies of d_2-protoadamantane diazirine (d_2-**13**).

Excitation Energy λ / nm	Oscillator Strength (f)
225.54	0.0036
215.34	0.0021
210.58	0.0016

Table S23: TD-B3LYP/6-311++G(3df,2pd) computed vertical excitation energies of d_2-protoadamantane diazo (d_2-**28**).

Excitation Energy λ / nm	Oscillator Strength (f)
260.55	0.0077
256	0.0070
253.15	0.0028
235.05	0.0305
231.06	0.0378
228.98	0.0039
226.11	0.0112
221.5	0.0063
210.33	0.0878

Table S24: TD-B3LYP/6-311++G(3df,2pd) computed vertical excitation energies of d_2-protoadamantylidene (d_2-**3**).

Excitation Energy λ / nm	Oscillator Strength (f)
531.88	0.0033
308.42	0.0018
271.5	0.0045
267.77	0.0037
259.35	0.0374
238.76	0.0019
233.55	0.0030
223.82	0.0038
207.31	0.0065

Table S25: TD-B3LYP/6-311++G(3df,2pd) computed vertical excitation energies of d_2-protoadamantane dehydro (d_2-**4**).

Excitation Energy λ / nm	Oscillator Strength (f)
210.48	0.0076

Table S26: TD-B3LYP/6-311++G(3df,2pd) computed vertical excitation energies of d_2-protoadamantane alkene (d_2-**12**).

Excitation Energy *λ* / nm	Oscillator Strength (*f*)
228.43	0.0135
208.54	0.0269
206.9	0.0151
202.41	0.0609
201.45	0.0082

Computed Potential Energy Surfaces

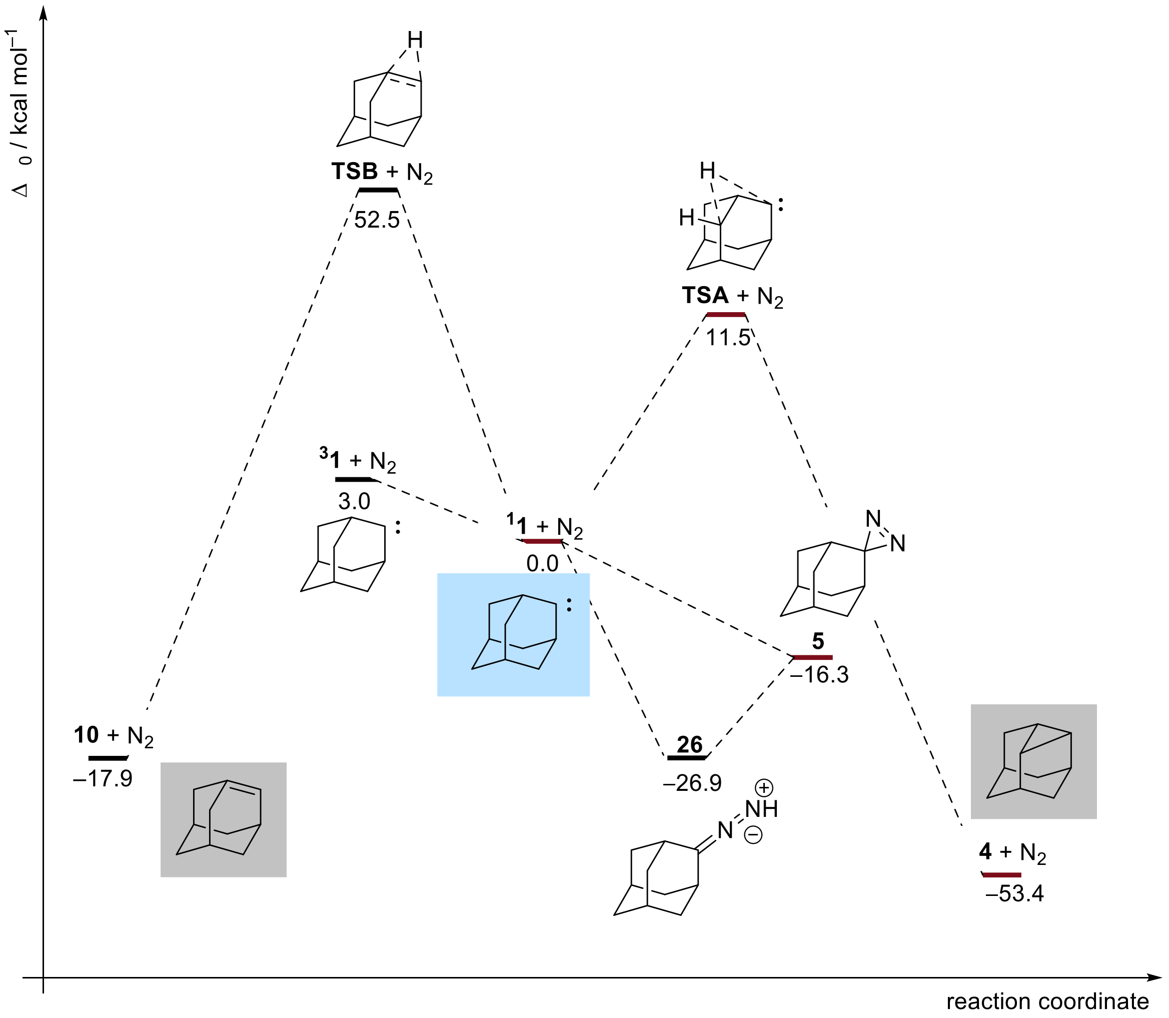

Figure S63: Potential energy profile (ΔH_0) in kcal mol^{-1} of the reactions of **1** at UB3LYP/def2TZVPP+ZPVE at 0 K.

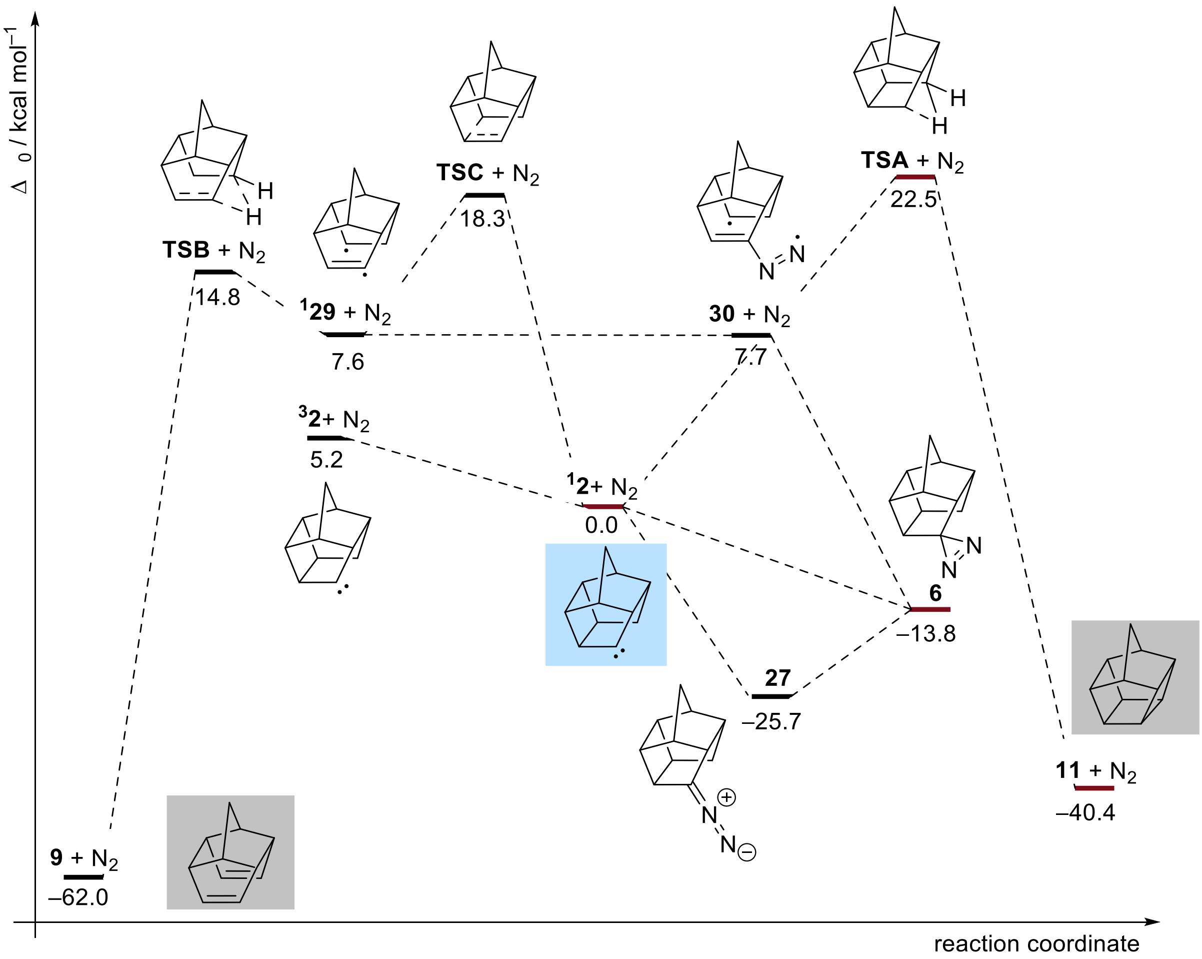

Figure S64: Potential energy profile (ΔH_0) in kcal mol^{-1} of the reactions of **3** at UB3LYP/def2TZVPP+ZPVE at 0 K.

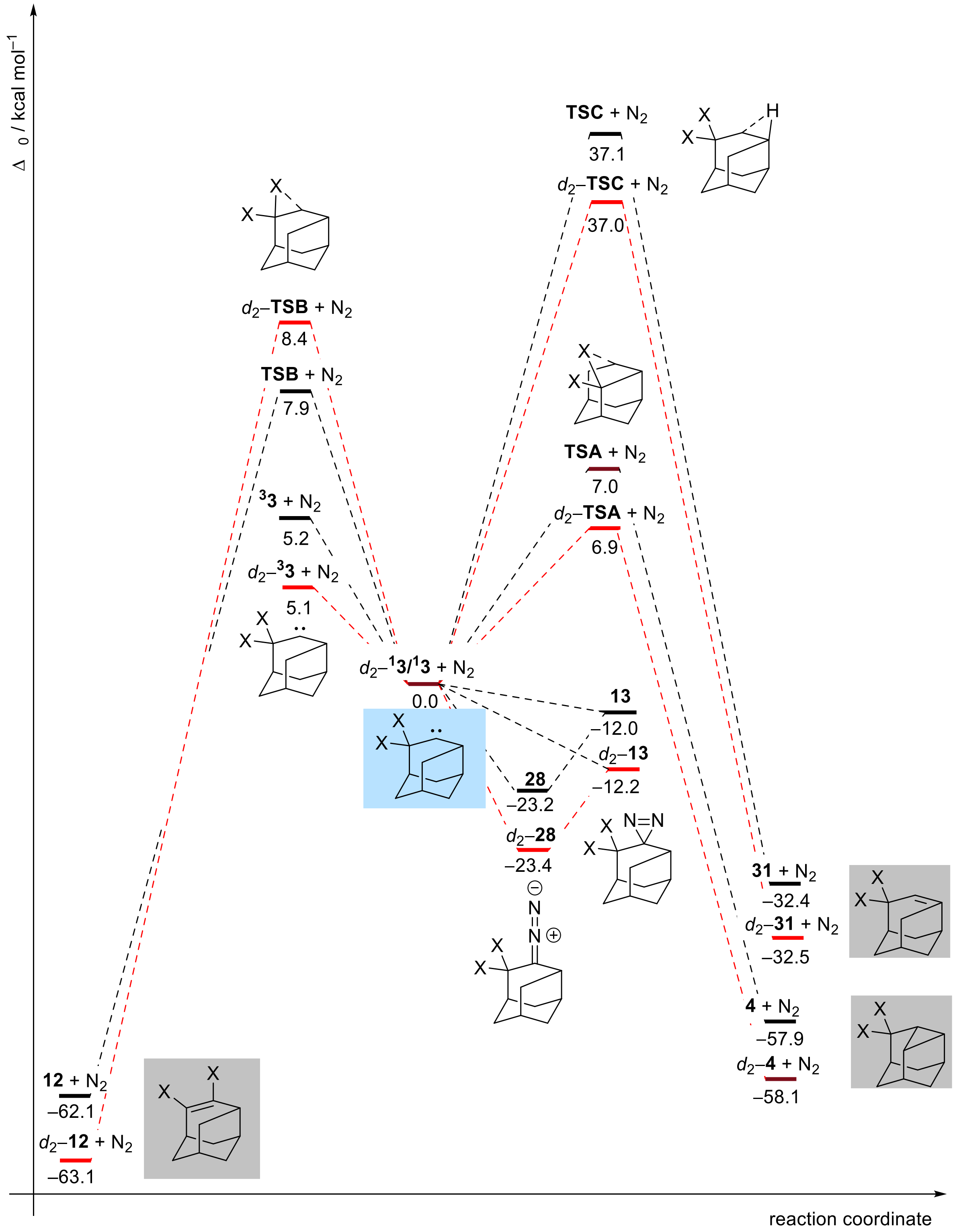

Figure S65: Potential energy profile (ΔH_0) in kcal mol^{-1} of the reactions of **13** and *d*$_2$–**13** at UB3LYP/def2TZVPP+ZPVE at 0 K. (X = H/D)

Tunneling Computations

Table S27: Overview of computed QMT half-lives and rate constants at the CVT/SCT//B3LYP/def2-TZVPP level of theory.

Reaction	QMT Half-Life (s^{-1}) at 10 K	Rate constants CVT/SCT 10 K
adamantane TSA (**1** to **4**)	5457851815	1.27×10^{-10}
adamantane TSB (**1** to **10**)	$> 3.28 \times 10^{30}$	$< 2.11 \times 10^{-31}$
PCU TSA (**2** to **11**)	$> 2.27 \times 10^{18}$	$< 3.05 \times 10^{-19}$
PCU TSB (**29** to **9**)		
PCU TSC (**2** to **29**)		
protoadamantane TSA (**3** to **4**)	3.30×10^{-5}	2.10×10^{4}
protoadamantane TSB (**3** to **12**)	4.01×10^{-3}	1.73×10^{2}
d_2-protoadamantane TSA (d_2-**3** to d_2-**4**)	2.75×10^{-5}	2.52×10^{4}
d_2-protoadamantane TSB (d_2-**3** to d_2-**12**)	245.80	2.82×10^{-3}

Table S28: Overview of computed QMT half-lives and rate constants at the CVT/SCT//B3LYP/6-31G(d) level of theory.

Reaction	QMT Half-Life (s^{-1}) at 10 K	Rate constants CVT/SCT 10 K
adamantane TSA (**1** to **4**)	70873.95	9.78×10^{-6}
adamantane TSB (**1** to **10**)	$> 3.08 \times 10^{26}$	$< 2.25 \times 10^{-27}$
PCU TSA (**2** to **11**)	$> 2.91 \times 10^{15}$	$< 2.38 \times 10^{-16}$
PCU TSB (**29** to **9**)	2.46×10^{-5}	2.82×10^{4}
PCU TSC (**2** to **29**)	$> 8.15 \times 10^{37}$	$< 8.50 \times 10^{-39}$
protoadamantane TSA (**3** to **4**)	1.35×10^{-5}	5.14×10^{4}
protoadamantane TSB (**3** to **12**)	1.18	5.88×10^{-1}
d_2-protoadamantane TSA (d_2-**3** to d_2-**4**)	1.13×10^{-5}	6.13×10^{4}
d_2-protoadamantane TSB (d_2-**3** to d_2-**12**)	50228.06	1.38×10^{-5}

Cartesian Coordinates of Computed Geometries

In the following, the cartesian coordinates of all computationally optimized geometries are given in Ångstrom. Electronic energies (in hartree mol^{-1}) and zero-point vibrational energies (ZPVE, in hartree) are provided.

B3LYP/6-311++G(3df,2pd)

Adamantane diazirine (C_{2v})

6	0.000000000	0.000000000	-2.269240941
6	1.259223000	0.000000000	-1.383939941
6	-1.259223000	0.000000000	-1.383939941
1	0.000000000	-0.878020000	-2.920655941
1	0.000000000	0.878020000	-2.920655941
6	1.261032000	-1.257869000	-0.495210941
6	-1.261032000	-1.257869000	-0.495210941
6	0.000000000	-1.264289000	0.394488059
1	-1.278536000	-2.157610000	-1.115276941
1	-2.158096000	-1.279732000	0.127724059
1	1.278536000	-2.157610000	-1.115276941
1	2.158096000	-1.279732000	0.127724059
6	-1.261032000	1.257869000	-0.495210941
1	-2.151432000	0.000000000	-2.014071941
6	1.261032000	1.257869000	-0.495210941
1	2.151432000	0.000000000	-2.014071941
6	0.000000000	0.000000000	1.226864059
1	0.000000000	-2.137851000	1.048525059
6	0.000000000	1.264289000	0.394488059
1	1.278536000	2.157610000	-1.115276941
1	2.158096000	1.279732000	0.127724059
1	-2.158096000	1.279732000	0.127724059
1	-1.278536000	2.157610000	-1.115276941
1	0.000000000	2.137851000	1.048525059
7	0.613341000	0.000000000	2.565146059
7	-0.613341000	0.000000000	2.565146059

E = −498.870751
ZVPE = 0.228429

2-Diazoadamantane (C_{2v})

6	0.000000000	0.000000000	2.352855992
6	-1.258517000	0.000000000	1.466089992
6	1.258517000	0.000000000	1.466089992
1	0.000000000	-0.878155000	3.004093992
1	0.000000000	0.878155000	3.004093992
6	-1.259571000	-1.263753000	0.583800992
6	1.259571000	-1.263753000	0.583800992
6	0.000000000	-1.273422000	-0.311123008
1	1.271583000	-2.157018000	1.214465992
1	2.156697000	-1.293411000	-0.038085008
1	-1.271583000	-2.157018000	1.214465992
1	-2.156697000	-1.293411000	-0.038085008
6	1.259571000	1.263753000	0.583800992
1	2.151057000	0.000000000	2.095906992
6	-1.259571000	1.263753000	0.583800992
1	-2.151057000	0.000000000	2.095906992
6	0.000000000	0.000000000	-1.129328008
1	0.000000000	-2.146777000	-0.963660008

6	0.000000000	1.273422000	-0.311123008
1	-1.271583000	2.157018000	1.214465992
1	-2.156697000	1.293411000	-0.038085008
1	2.156697000	1.293411000	-0.038085008
1	1.271583000	2.157018000	1.214465992
1	0.000000000	2.146777000	-0.963660008
7	0.000000000	0.000000000	-2.409272008
7	0.000000000	0.000000000	-3.553974008

E = −498.887387
ZVPE = 0.228512

Singlet adamantylidene (C_s)

6	-1.393498000	0.998124000	0.000000000
6	-1.407777000	-0.540077000	0.000000000
6	0.058278000	1.506596000	0.000000000
1	-1.922915000	1.377336000	0.878194000
1	-1.922915000	1.377336000	-0.878194000
6	-0.682991000	-1.067713000	1.244417000
6	0.791386000	0.990311000	1.244268000
6	0.791386000	-0.566863000	1.236963000
1	0.308747000	1.336459000	2.162335000
1	1.817675000	1.362553000	1.262772000
1	-1.165990000	-0.721701000	2.162325000
1	-0.705201000	-2.159195000	1.263396000
6	0.791386000	0.990311000	-1.244268000
1	0.064374000	2.599650000	0.000000000
6	-0.682991000	-1.067713000	-1.244417000
1	-2.440652000	-0.897784000	0.000000000
6	1.461269000	-1.047298000	0.000000000
1	1.302319000	-0.932829000	2.130089000
6	0.791386000	-0.566863000	-1.236963000
1	-1.165990000	-0.721701000	-2.162325000
1	-0.705201000	-2.159195000	-1.263396000
1	1.817675000	1.362553000	-1.262772000
1	0.308747000	1.336459000	-2.162335000
1	1.302319000	-0.932829000	-2.130089000

E = −389.290947
ZVPE = 0.216203

Triplet adamantylidene (C_1)

6	1.379712000	0.985715000	0.000000000
6	-0.072813000	1.496961000	0.000000000
6	1.392511000	-0.553913000	0.000000000
1	1.909549000	1.364312000	0.878349000
1	1.909549000	1.364312000	-0.878349000
6	-0.792339000	0.990667000	1.265085000
6	0.680030000	-1.070474000	1.265420000
6	-0.792339000	-0.566182000	1.269550000
1	1.196528000	-0.711803000	2.160946000
1	0.695046000	-2.161402000	1.294355000
1	-0.285466000	1.362453000	2.160622000
1	-1.819693000	1.357742000	1.293920000
6	0.680030000	-1.070474000	-1.265420000
1	2.425650000	-0.910258000	0.000000000
6	-0.792339000	0.990667000	-1.265085000
1	-0.074415000	2.589897000	0.000000000

6	-1.407285000	-1.006388000	0.000000000
1	-1.320071000	-0.942310000	2.147919000
6	-0.792339000	-0.566182000	-1.269550000
1	-0.285466000	1.362453000	-2.160622000
1	-1.819693000	1.357742000	-1.293920000
1	0.695046000	-2.161402000	-1.294355000
1	1.196528000	-0.711803000	-2.160946000
1	-1.320071000	-0.942310000	-2.147919000

E = −389.282811
ZVPE = 0.216850

2,4-Dehydroadamantane (C_s)

6	-0.207780028	-1.142344494	1.271884000
6	-1.079812471	-1.107049711	0.000000000
6	0.902536535	-0.078368265	1.185929000
1	0.248008171	-2.127928978	1.396585000
1	-0.838767848	-0.970810261	2.147738000
6	-0.207780028	-1.142344494	-1.271884000
6	1.824189046	-0.433306336	0.000000000
6	0.902536535	-0.078368265	-1.185929000
1	-0.838767848	-0.970810261	-2.147738000
1	0.248008171	-2.127928978	-1.396585000
1	2.155210812	-1.475002170	0.000000000
1	2.709716652	0.205187965	0.000000000
6	-1.887753076	0.196243804	0.000000000
1	-1.763864607	-1.958211030	0.000000000
6	0.361327169	1.302176527	0.759739000
1	1.438323755	-0.013364316	2.133047000
6	0.361327169	1.302176527	-0.759739000
1	1.438323755	-0.013364316	-2.133047000
6	-0.941496991	1.373159073	0.000000000
1	-2.537355652	0.237789693	0.879026000
1	-2.537355652	0.237789693	-0.879026000
1	-1.418035383	2.346955939	0.000000000
1	0.685786171	2.171945526	1.314605000
1	0.685786171	2.171945526	-1.314605000

E = −389.374314
ZVPE = 0.218794

Adamantane Alkene (C_1)

6	-0.232226000	-0.960438000	1.391463000
6	1.027194000	-0.139720000	1.048315000
6	-1.282839000	-0.818836000	0.272097000
1	-0.644425000	-0.618927000	2.344223000
1	0.026351000	-2.015050000	1.520180000
6	0.591987000	1.338525000	0.884997000
6	-1.685605000	0.653023000	0.053837000
6	-0.455658000	1.495986000	-0.301844000
1	1.454943000	1.980747000	0.704094000
1	0.129057000	1.677880000	1.815778000
1	-2.147460000	1.058524000	0.957696000
1	-2.433071000	0.717181000	-0.739452000
6	1.713629000	-0.718643000	-0.281878000
1	1.754759000	-0.214709000	1.860441000
6	-0.628733000	-1.294918000	-1.034917000
1	-2.167135000	-1.414019000	0.514314000
6	0.593028000	-0.426199000	-1.239491000
1	1.969163000	-1.773222000	-0.148378000

1	2.620286000	-0.150572000	-0.491514000
1	-1.316643000	-1.194604000	-1.873773000
1	-0.344563000	-2.347428000	-0.968967000
6	0.436647000	0.914932000	-1.387787000
1	-0.724760000	2.545697000	-0.432162000
1	1.358952000	1.486229000	-1.491234000

E = -389.315485
ZVPE = 0.217172

PCU diazirine (C_1)

6	1.440544000	0.643028000	0.914295000
6	0.199074000	1.568958000	0.702010000
6	0.124202000	1.779118000	-0.811959000
6	0.626665000	0.420270000	-1.322832000
1	0.806628000	2.572998000	-1.123452000
1	-0.872725000	2.040344000	-1.165481000
6	1.911275000	0.166612000	-0.485498000
6	0.502592000	-0.515797000	1.380534000
6	-0.749209000	0.402805000	1.176447000
6	-1.443528000	-0.223492000	0.001268000
6	-0.326339000	-0.762927000	-0.855939000
6	0.556024000	-1.512170000	0.186562000
1	0.665343000	-0.956423000	2.360366000
1	2.228296000	0.971849000	1.587303000
1	0.796141000	0.390946000	-2.397665000
6	1.983636000	-1.356941000	-0.344221000
1	0.215938000	-2.519869000	0.414755000
1	2.150154000	-1.871684000	-1.291933000
1	2.741887000	-1.687314000	0.367739000
1	2.798278000	0.666763000	-0.870018000
1	-1.385122000	0.613118000	2.032233000
1	0.141341000	2.476836000	1.297318000
1	-0.664577000	-1.379867000	-1.685603000
7	-2.748905000	-0.888836000	0.105475000
7	-2.761267000	0.178196000	-0.501127000

E = -535.739023
ZVPE = 0.210977

PCU diazo (C_1)

6	1.549567000	0.752257000	0.777641000
6	0.276083000	1.624642000	0.540238000
6	0.099130000	1.658650000	-0.978321000
6	0.616610000	0.267939000	-1.370536000
1	0.720981000	2.436718000	-1.426350000
1	-0.932862000	1.838923000	-1.283316000
6	1.952189000	0.135575000	-0.588226000
6	0.673298000	-0.370011000	1.420352000
6	-0.617465000	0.494688000	1.197088000
6	-1.351314000	-0.272527000	0.141273000
6	-0.280115000	-0.882340000	-0.720716000
6	0.686688000	-1.488873000	0.343948000
1	0.899755000	-0.695575000	2.432282000
1	2.365446000	1.168149000	1.363398000
1	0.723793000	0.124582000	-2.444700000
6	2.075160000	-1.362104000	-0.288591000
1	0.386285000	-2.472058000	0.698923000

1	2.198949000	-1.973750000	-1.183987000
1	2.883972000	-1.596535000	0.405479000
1	2.802422000	0.609482000	-1.075620000
1	-1.202314000	0.788590000	2.064101000
1	0.225710000	2.592591000	1.033897000
1	-0.642488000	-1.593836000	-1.458655000
7	-2.613340000	-0.302085000	-0.060279000
7	-3.745036000	-0.351438000	-0.221201000

E = −535.757551
ZVPE = 0.210894

PCU singlet carbene (C_1)

6	0.013489000	0.349912000	-1.367941000
6	1.451312000	0.261463000	-0.767626000
6	1.450275000	1.250793000	0.396441000
6	0.013440000	1.080437000	0.904385000
1	1.591296000	2.268062000	0.021483000
1	2.215876000	1.055104000	1.145845000
6	-0.843281000	1.192157000	-0.382469000
6	-0.281714000	-1.114923000	-0.925994000
6	1.149283000	-1.239290000	-0.293004000
6	0.975390000	-1.222840000	1.174693000
6	-0.273826000	-0.437024000	1.366304000
6	-1.263827000	-0.934365000	0.259152000
1	-0.574883000	-1.836618000	-1.684185000
1	-0.094211000	0.605946000	-2.418982000
1	-0.270181000	1.773798000	1.693249000
6	-2.067575000	0.313446000	-0.117325000
1	-1.831041000	-1.822502000	0.529846000
1	-2.696148000	0.687006000	0.692687000
1	-2.693160000	0.164536000	-0.999856000
1	-1.033739000	2.218206000	-0.693836000
1	1.836349000	-1.981744000	-0.690210000
1	2.293393000	0.334037000	-1.450407000
1	-0.681343000	-0.464435000	2.374670000

E = −426.161270
ZVPE = 0.198940

PCU triplet carbene (C_1)

6	0.054221000	0.319774000	-1.371245000
6	1.482000000	0.257621000	-0.744364000
6	1.442476000	1.241925000	0.423748000
6	-0.013979000	1.105636000	0.886800000
1	1.638498000	2.261271000	0.081140000
1	2.165737000	1.001312000	1.204058000
6	-0.830183000	1.185018000	-0.431260000
6	-0.250258000	-1.135116000	-0.898543000
6	1.193284000	-1.234005000	-0.255381000
6	0.881749000	-1.226860000	1.178085000
6	-0.319441000	-0.391541000	1.390426000
6	-1.270008000	-0.923505000	0.259032000
1	-0.532475000	-1.879824000	-1.638579000
1	-0.034486000	0.540303000	-2.432303000
1	-0.311933000	1.839879000	1.633761000
6	-2.062253000	0.309196000	-0.182135000
1	-1.834566000	-1.808269000	0.543031000
1	-2.719621000	0.699476000	0.596978000

1	-2.655396000	0.137680000	-1.082388000
1	-1.015614000	2.200211000	-0.779155000
1	1.873338000	-1.992683000	-0.631449000
1	2.333746000	0.360019000	-1.413019000
1	-0.752879000	-0.408230000	2.386941000

E = -426.150287
ZVPE = 0.199445

PCU homopentaprismane (C_{2v})

6	-1.205610000	0.787212000	0.173871000
6	-0.777021000	0.782659000	-1.321239000
6	0.000000000	1.143320000	1.073497000
6	1.205610000	0.787212000	0.173871000
6	0.777021000	0.782659000	-1.321239000
6	-1.205610000	-0.787212000	0.173871000
1	-2.139026000	1.282978000	0.431935000
6	1.205610000	-0.787212000	0.173871000
1	2.139026000	1.282978000	0.431935000
6	0.000000000	-1.143320000	1.073497000
6	-0.777021000	-0.782659000	-1.321239000
1	-1.315366000	1.390502000	-2.043697000
1	-2.139026000	-1.282978000	0.431935000
6	0.777021000	-0.782659000	-1.321239000
1	1.315366000	1.390502000	-2.043697000
1	2.139026000	-1.282978000	0.431935000
1	-1.315366000	-1.390502000	-2.043697000
1	1.315366000	-1.390502000	-2.043697000
6	0.000000000	0.000000000	2.110176000
1	0.000000000	2.156947000	1.471609000
1	-0.885174000	0.000000000	2.748812000
1	0.885174000	0.000000000	2.748812000
1	0.000000000	-2.156947000	1.471609000

E = -426.226649
ZVPE = 0.200716

PCU dialkene_1 (C_{2v})

6	1.173949000	0.806088000	0.078802000
6	1.173949000	-0.806088000	0.078802000
6	0.000000000	1.117958000	1.056171000
6	0.000000000	-1.117958000	1.056171000
6	-1.173949000	-0.806088000	0.078802000
6	-1.173949000	0.806088000	0.078802000
6	0.000000000	0.000000000	2.082222000
1	0.000000000	2.141169000	1.427959000
1	-0.885862000	0.000000000	2.720429000
1	0.885862000	0.000000000	2.720429000
1	0.000000000	-2.141169000	1.427959000
6	0.665684000	1.423961000	-1.204697000
1	2.141000000	1.185302000	0.406603000
6	-0.665684000	1.423961000	-1.204697000
1	-2.141000000	1.185302000	0.406603000
1	1.300996000	1.747444000	-2.017270000
1	-1.300996000	1.747444000	-2.017270000
6	-0.665684000	-1.423961000	-1.204697000
1	-2.141000000	-1.185302000	0.406603000
6	0.665684000	-1.423961000	-1.204697000
1	2.141000000	-1.185302000	0.406603000
1	-1.300996000	-1.747444000	-2.017270000

1	1.300996000	-1.747444000	-2.017270000

E = – 426.260526
ZVPE = 0.199790

PCU dialkene_2 (C_{2v})

6	1.320392000	0.788454000	0.211280000
6	1.320392000	-0.788454000	0.211280000
6	0.000000000	1.147674000	0.939879000
6	0.000000000	-1.147674000	0.939879000
6	-1.320392000	-0.788454000	0.211280000
6	-1.320392000	0.788454000	0.211280000
6	0.000000000	0.000000000	1.995676000
1	0.000000000	2.156383000	1.350653000
1	-0.885851000	0.000000000	2.633257000
1	0.885851000	0.000000000	2.633257000
1	0.000000000	-2.156383000	1.350653000
6	1.525141000	-0.666606000	-1.289788000
1	2.159552000	-1.247942000	0.738913000
6	1.525141000	0.666606000	-1.289788000
1	2.159552000	1.247942000	0.738913000
6	-1.525141000	-0.666606000	-1.289788000
1	-2.159552000	-1.247942000	0.738913000
6	-1.525141000	0.666606000	-1.289788000
1	-2.159552000	1.247942000	0.738913000
1	-1.632840000	-1.405528000	-2.072967000
1	-1.632840000	1.405528000	-2.072967000
1	1.632840000	-1.405528000	-2.072967000
1	1.632840000	1.405528000	-2.072967000

E = -426.179384
ZVPE = 0.197019

Protoadamantane diazirine (C_1)

6	0.437832000	1.576145000	-0.139995000
1	0.568643000	2.658833000	-0.184227000
6	1.550441000	0.940792000	-1.012795000
6	1.773598000	-0.567460000	-0.774444000
1	2.591280000	-0.916125000	-1.407435000
6	0.492064000	-1.384573000	-0.987366000
6	-0.372360000	-1.058880000	0.271683000
6	-1.458215000	-0.078504000	-0.085804000
7	-2.769417000	-0.589234000	-0.519318000
7	-2.754532000	-0.139993000	0.619776000
6	-0.997563000	1.260930000	-0.619489000
6	2.020162000	-0.824690000	0.719009000
6	0.648590000	-0.446616000	1.305835000
1	0.489850000	-0.848202000	2.305832000
6	0.548972000	1.090619000	1.315943000
1	2.489641000	1.459822000	-0.801789000
1	1.329364000	1.121826000	-2.068049000
1	0.733023000	-2.447966000	-1.012128000
1	-0.025789000	-1.151366000	-1.917866000
1	-0.826913000	-1.963250000	0.673168000
1	-1.678209000	2.034656000	-0.256652000
1	-1.060528000	1.281496000	-1.711844000
1	2.832484000	-0.230488000	1.140141000
1	2.253760000	-1.877872000	0.890124000
1	1.429950000	1.508548000	1.807129000
1	-0.320048000	1.428093000	1.884931000

E = -498.857697

ZVPE = 0.227837

Protoadamantane diazo (C_1)

7	-3.744270000	-0.419073000	0.067033000
7	-2.621794000	-0.220544000	-0.024694000
6	-1.364437000	0.014403000	-0.130298000
6	-0.864967000	1.376584000	-0.552593000
6	0.574876000	1.574837000	-0.017906000
1	0.756104000	2.650372000	0.025635000
6	1.674718000	0.958561000	-0.921621000
6	1.845053000	-0.570257000	-0.790715000
1	2.666343000	-0.896861000	-1.430756000
6	0.545991000	-1.330178000	-1.086922000
6	-0.343469000	-1.051553000	0.168961000
6	2.050598000	-0.943587000	0.684608000
6	0.680090000	-0.561369000	1.269588000
1	0.479859000	-1.032492000	2.231350000
6	0.633488000	0.972735000	1.396428000
1	-1.511183000	2.153203000	-0.137452000
1	-0.884912000	1.500732000	-1.641255000
1	2.628917000	1.426733000	-0.663902000
1	1.474842000	1.222540000	-1.963527000
1	0.755590000	-2.398111000	-1.169214000
1	0.057689000	-1.022875000	-2.011118000
1	-0.848673000	-1.959161000	0.493627000
1	2.872830000	-0.409486000	1.163178000
1	2.246220000	-2.013659000	0.782542000
1	1.515626000	1.320070000	1.937899000
1	-0.238452000	1.295262000	1.969446000

E = −498.875073

ZVPE = 0.227894

Singlet protoadamantylidene (C_1)

6	1.087201000	-1.043019000	-0.241525000
1	1.762591000	-1.881484000	-0.429856000
6	-0.047614000	-1.089634000	-1.290410000
6	-1.223537000	-0.143768000	-0.962369000
1	-1.981314000	-0.216480000	-1.745344000
6	-0.756824000	1.306436000	-0.789109000
6	-0.104387000	1.307954000	0.683157000
6	1.240260000	1.525548000	0.155622000
6	1.890954000	0.269497000	-0.312810000
6	-1.771173000	-0.461768000	0.433118000
6	-0.575074000	-0.051667000	1.310854000
1	-0.851726000	0.097230000	2.354370000
6	0.510289000	-1.128652000	1.179368000
1	-0.437326000	-2.109410000	-1.352819000
1	0.357670000	-0.851002000	-2.277702000
1	-1.582026000	2.016027000	-0.780150000
1	-0.072345000	1.641656000	-1.572023000
1	-0.487218000	2.157249000	1.246257000
1	2.765685000	0.212649000	0.355217000
1	2.342083000	0.433921000	-1.296702000
1	-2.054065000	-1.508476000	0.564650000
1	-2.650370000	0.150350000	0.647250000
1	0.079857000	-2.112476000	1.380765000
1	1.307935000	-0.975312000	1.910707000

E = −389.281186

ZVPE = 0.215491

Triplet protoadamantylidene (C_1)

6	1.176520000	-0.952570000	-0.185699000
1	1.870783000	-1.787537000	-0.303729000
6	0.005436000	-1.170286000	-1.179622000
6	-1.279023000	-0.359252000	-0.886279000
1	-2.051710000	-0.648438000	-1.601087000
6	-1.041639000	1.154640000	-0.881695000
6	-0.224894000	1.389618000	0.442712000
6	1.206272000	1.475382000	0.129026000
6	1.981020000	0.361105000	-0.442885000
6	-1.705682000	-0.572644000	0.573081000
6	-0.531517000	0.086282000	1.315478000
1	-0.768528000	0.353996000	2.344249000
6	0.659920000	-0.887189000	1.262235000
1	-0.261317000	-2.230923000	-1.159046000
1	0.350757000	-0.962122000	-2.195843000
1	-1.998176000	1.679350000	-0.835515000
1	-0.506496000	1.521956000	-1.756466000
1	-0.575753000	2.282328000	0.960165000
1	2.962657000	0.274474000	0.035554000
1	2.177061000	0.482530000	-1.517475000
1	-1.833123000	-1.621859000	0.845424000
1	-2.648965000	-0.059331000	0.771453000
1	0.337969000	-1.871192000	1.610642000
1	1.466364000	-0.563758000	1.923562000

E = −389.272711

ZVPE = 0.215979

2,4-Dehydroprotoadamantane (C_1)

6	1.069611000	-1.099318000	0.000000000
1	1.747280000	-1.955579000	0.000000000
6	0.197587000	-1.128212000	-1.271886000
6	-0.905284000	-0.056598000	-1.186028000
1	-1.440672000	0.012375000	-2.133026000
6	-0.354025000	1.320144000	-0.759563000
1	-0.672292000	2.192550000	-1.313845000
6	-0.354024000	1.320144000	0.759563000
6	0.949129000	1.381760000	-0.000001000
1	1.433246000	2.351812000	-0.000001000
6	1.887017000	0.197786000	-0.000001000
6	-1.829032000	-0.405314000	0.000001000
6	-0.905283000	-0.056597000	1.186029000
1	-1.440671000	0.012377000	2.133028000
6	0.197588000	-1.128211000	1.271888000
1	-0.265027000	-2.110598000	-1.396902000
1	0.829642000	-0.961025000	-2.147791000
1	-0.672291000	2.192551000	1.313844000
1	2.536963000	0.234603000	0.878941000
1	2.536962000	0.234602000	-0.878943000
1	-2.167290000	-1.444714000	0.000002000
1	-2.710169000	0.239163000	0.000001000
1	-0.265026000	-2.110597000	1.396904000
1	0.829644000	-0.961024000	2.147792000

E = −389.374298

ZVPE = 0.218812

Protoadamantane alkene (C_1)

6	-0.195200000	-1.121905000	-1.250053000
6	1.024627000	-1.124609000	-0.282965000
6	-1.335593000	-0.145118000	-0.865436000
1	-0.616474000	-2.131938000	-1.269035000
1	0.148573000	-0.909224000	-2.264997000
6	0.537069000	-1.102769000	1.182693000
6	-1.745417000	-0.369357000	0.595980000
6	-0.455057000	0.061969000	1.317239000
1	1.394764000	-0.988328000	1.846083000
1	0.038735000	-2.037052000	1.452368000
1	-2.026441000	-1.398468000	0.825504000
1	-2.591363000	0.272264000	0.853331000
6	1.915471000	0.091597000	-0.417580000
1	1.607361000	-2.026629000	-0.477938000
6	-0.882095000	1.318293000	-0.778648000
1	-2.165135000	-0.275273000	-1.563784000
6	1.460890000	1.251211000	0.045398000
6	0.025015000	1.335818000	0.495456000
1	-0.617604000	0.315304000	2.364440000
1	-1.757862000	1.956613000	-0.640640000
1	-0.350443000	1.671745000	-1.660990000
1	-0.141375000	2.242792000	1.075544000
1	2.916099000	-0.014648000	-0.818954000
1	2.062894000	2.152059000	0.026574000

E = −389.379737
ZVPE = 0.218349

d_2-Protoadamantane diazirine (C_1)

6	0.437832000	1.576145000	-0.139995000
1	0.568643000	2.658833000	-0.184227000
6	1.550441000	0.940792000	-1.012795000
6	1.773598000	-0.567460000	-0.774444000
1	2.591280000	-0.916125000	-1.407435000
6	0.492064000	-1.384573000	-0.987366000
6	-0.372360000	-1.058880000	0.271683000
6	-1.458215000	-0.078504000	-0.085804000
7	-2.769417000	-0.589234000	-0.519318000
7	-2.754532000	-0.139993000	0.619776000
6	-0.997563000	1.260930000	-0.619489000
6	2.020162000	-0.824690000	0.719009000
6	0.648590000	-0.446616000	1.305835000
1	0.489850000	-0.848202000	2.305832000
6	0.548972000	1.090619000	1.315943000
1	2.489641000	1.459822000	-0.801789000
1	1.329364000	1.121826000	-2.068049000
1	0.733023000	-2.447966000	-1.012128000
1	-0.025789000	-1.151366000	-1.917866000
1	-0.826913000	-1.963250000	0.673168000
1(Iso=2)	-1.678209000	2.034656000	-0.256652000
1(Iso=2)	-1.060528000	1.281496000	-1.711844000
1	2.832484000	-0.230488000	1.140141000
1	2.253760000	-1.877872000	0.890124000
1	1.429950000	1.508548000	1.807129000
1	-0.320048000	1.428093000	1.884931000

E = −498.864337

ZVPE = 0.221196

d_2-Protoadamantane diazo (C_1)

7	-3.744270000	-0.419073000	0.067033000
7	-2.621794000	-0.220544000	-0.024694000
6	-1.364437000	0.014403000	-0.130298000
6	-0.864967000	1.376584000	-0.552593000
6	0.574876000	1.574837000	-0.017906000
1	0.756104000	2.650372000	0.025635000
6	1.674718000	0.958561000	-0.921621000
6	1.845053000	-0.570257000	-0.790715000
1	2.666343000	-0.896861000	-1.430756000
6	0.545991000	-1.330178000	-1.086922000
6	-0.343469000	-1.051553000	0.168961000
6	2.050598000	-0.943587000	0.684608000
6	0.680090000	-0.561369000	1.269588000
1	0.479859000	-1.032492000	2.231350000
6	0.633488000	0.972735000	1.396428000
1(Iso=2)	-1.511183000	2.153203000	-0.137452000
1(Iso=2)	-0.884912000	1.500732000	-1.641255000
1	2.628917000	1.426733000	-0.663902000
1	1.474842000	1.222540000	-1.963527000
1	0.755590000	-2.398111000	-1.169214000
1	0.057689000	-1.022875000	-2.011118000
1	-0.848673000	-1.959161000	0.493627000
1	2.872830000	-0.409486000	1.163178000
1	2.246220000	-2.013659000	0.782542000
1	1.515626000	1.320070000	1.937899000
1	-0.238452000	1.295262000	1.969446000

E = - 498.881703

ZVPE = 0.221264

d_2-Singlet protoadamantylidene (C_1)

6	1.087201000	-1.043019000	-0.241525000
1	1.762591000	-1.881484000	-0.429856000
6	-0.047614000	-1.089634000	-1.290410000
6	-1.223537000	-0.143768000	-0.962369000
1	-1.981314000	-0.216480000	-1.745344000
6	-0.756824000	1.306436000	-0.789109000
6	-0.104387000	1.307954000	0.683157000
6	1.240260000	1.525548000	0.155622000
6	1.890954000	0.269497000	-0.312810000
6	-1.771173000	-0.461768000	0.433118000
6	-0.575074000	-0.051667000	1.310854000
1	-0.851726000	0.097230000	2.354370000
6	0.510289000	-1.128652000	1.179368000
1	-0.437326000	-2.109410000	-1.352819000
1	0.357670000	-0.851002000	-2.277702000
1	-1.582026000	2.016027000	-0.780150000
1	-0.072345000	1.641656000	-1.572023000
1	-0.487218000	2.157249000	1.246257000
1(Iso=2)	2.765685000	0.212649000	0.355217000
1(Iso=2)	2.342083000	0.433921000	-1.296702000
1	-2.054065000	-1.508476000	0.564650000
1	-2.650370000	0.150350000	0.647250000
1	0.079857000	-2.112476000	1.380765000
1	1.307935000	-0.975312000	1.910707000

E = −389.287561

ZVPE = 0.209115

d_2-Triplet protoadamantylidene (C_1)

6	1.176540000	-0.952558000	-0.185677000
1	1.870802000	-1.787530000	-0.303688000
6	0.005450000	-1.170318000	-1.179592000
6	-1.279027000	-0.359297000	-0.886264000
1	-2.051710000	-0.648518000	-1.601059000
6	-1.041683000	1.154594000	-0.881721000
6	-0.224916000	1.389627000	0.442671000
6	1.206253000	1.475388000	0.128984000
6	1.981016000	0.361113000	-0.442914000
6	-1.705669000	-0.572649000	0.573108000
6	-0.531510000	0.086320000	1.315481000
1	-0.768520000	0.354050000	2.344250000
6	0.659951000	-0.887129000	1.262259000
1	-0.261286000	-2.230959000	-1.158981000
1	0.350768000	-0.962171000	-2.195813000
1	-1.998234000	1.679272000	-0.835536000
1	-0.506572000	1.521909000	-1.756515000
1	-0.575761000	2.282354000	0.960098000
1	2.962660000	0.274510000	0.035516000
1	2.177033000	0.482519000	-1.517507000
1	-1.833078000	-1.621859000	0.845480000
1	-2.648967000	-0.059360000	0.771475000
1	0.338032000	-1.871122000	1.610714000
1	1.466397000	-0.563645000	1.923561000

E = −389.279205

ZVPE = 0.209485

d_2-2,4-Dehydroprotoadamantane (C_1)

6	-1.069612000	-1.099318000	0.000001000
1	-1.747282000	-1.955578000	0.000001000
6	-0.197589000	-1.128209000	1.271888000
6	0.905283000	-0.056597000	1.186028000
1	1.440671000	0.012377000	2.133028000
6	0.354026000	1.320145000	0.759563000
1	0.672293000	2.192551000	1.313843000
6	0.354027000	1.320143000	-0.759564000
6	-0.949128000	1.381761000	-0.000002000
1	-1.433244000	2.351813000	-0.000003000
6	-1.887017000	0.197787000	0.000000000
6	1.829032000	-0.405315000	0.000001000
6	0.905284000	-0.056599000	-1.186028000
1	1.440673000	0.012373000	-2.133026000
6	-0.197588000	-1.128213000	-1.271886000
1	0.265024000	-2.110596000	1.396905000
1	-0.829645000	-0.961022000	2.147793000
1	0.672293000	2.192548000	-1.313846000
1(Iso=2)	-2.536962000	0.234603000	-0.878944000
1(Iso=2)	-2.536963000	0.234605000	0.878941000
1	2.167289000	-1.444716000	0.000002000
1	2.710168000	0.239161000	0.000001000
1	0.265026000	-2.110599000	-1.396901000
1	-0.829643000	-0.961026000	-2.147792000

E = −389.380965

ZVPE = 0.212146

d_2-Protoadamantane alkene (C_1)

6	-0.195200000	-1.121905000	-1.250053000
6	1.024627000	-1.124609000	-0.282965000
6	-1.335593000	-0.145118000	-0.865436000
1	-0.616474000	-2.131938000	-1.269035000
1	0.148573000	-0.909224000	-2.264997000
6	0.537069000	-1.102769000	1.182693000
6	-1.745417000	-0.369357000	0.595980000
6	-0.455057000	0.061969000	1.317239000
1	1.394764000	-0.988328000	1.846083000
1	0.038735000	-2.037052000	1.452368000
1	-2.026441000	-1.398468000	0.825504000
1	-2.591363000	0.272264000	0.853331000
6	1.915471000	0.091597000	-0.417580000
1	1.607361000	-2.026629000	-0.477938000
6	-0.882095000	1.318293000	-0.778648000
1	-2.165135000	-0.275273000	-1.563784000
6	1.460890000	1.251211000	0.045398000
6	0.025015000	1.335818000	0.495456000
1(Iso=2)	-0.617604000	0.315304000	2.364440000
1(Iso=2)	-1.757862000	1.956613000	-0.640640000
1	-0.350443000	1.671745000	-1.660990000
1	-0.141375000	2.242792000	1.075544000
1	2.916099000	-0.014648000	-0.818954000
1	2.062894000	2.152059000	0.026574000

E = −389.386135
ZVPE = 0.211951

B3LYP/def2TZVPP

Adamantane diazirine (C_{2v})

6	0.000000000	0.000000000	2.197777000
6	0.000000000	1.256798000	1.314449000
6	0.000000000	-1.256798000	1.314449000
1	-0.878974000	0.000000000	2.847533000
1	0.878974000	0.000000000	2.847533000
6	-1.255300000	1.258606000	0.427538000
6	-1.255300000	-1.258606000	0.427538000
6	-1.261975000	0.000000000	-0.459212000
1	-2.154370000	-1.276237000	1.048201000
1	-1.274463000	-2.154068000	-0.197476000
1	-2.154370000	1.276237000	1.048201000
1	-1.274463000	2.154068000	-0.197476000
6	1.255300000	-1.258606000	0.427538000
1	0.000000000	-2.148703000	1.944502000
6	1.255300000	1.258606000	0.427538000
1	0.000000000	2.148703000	1.944502000
6	0.000000000	0.000000000	-1.287745000
1	-2.134084000	0.000000000	-1.114711000
6	1.261975000	0.000000000	-0.459212000
1	2.154370000	1.276237000	1.048201000
1	1.274463000	2.154068000	-0.197476000
1	1.274463000	-2.154068000	-0.197476000
1	2.154370000	-1.276237000	1.048201000
1	2.134084000	0.000000000	-1.114711000
7	0.000000000	0.613029000	-2.624393000
7	0.000000000	-0.613029000	-2.624393000

E = −498.947862

ZVPE = 0.229215

2-Diazoadamantane (C_{2v})

6	0.000000000	0.000000000	-2.274801000
6	-1.255950000	0.000000000	-1.389963000
6	1.255950000	0.000000000	-1.389963000
1	0.000000000	0.879016000	-2.924392000
1	0.000000000	-0.879016000	-2.924392000
6	-1.256968000	1.261449000	-0.509729000
6	1.256968000	1.261449000	-0.509729000
6	0.000000000	1.271169000	0.383131000
1	1.268346000	2.153771000	-1.141268000
1	2.152867000	1.289053000	0.113539000
1	-1.268346000	2.153771000	-1.141268000
1	-2.152867000	1.289053000	0.113539000
6	1.256968000	-1.261449000	-0.509729000
1	2.148137000	0.000000000	-2.019667000
6	-1.256968000	-1.261449000	-0.509729000
1	-2.148137000	0.000000000	-2.019667000
6	0.000000000	0.000000000	1.198397000
1	0.000000000	2.143423000	1.036669000
6	0.000000000	-1.271169000	0.383131000
1	-1.268346000	-2.153771000	-1.141268000
1	-2.152867000	-1.289053000	0.113539000
1	2.152867000	-1.289053000	0.113539000
1	1.268346000	-2.153771000	-1.141268000
1	0.000000000	-2.143423000	1.036669000
7	0.000000000	0.000000000	2.478210000
7	0.000000000	0.000000000	3.621733000

E = –498.964783
ZVPE = 0.229292

Singlet adamantylidene (C_s)

6	0.000000000	0.000000000	-1.716425000
6	-1.255625000	0.000000000	-0.834336000
6	1.255625000	0.000000000	-0.834336000
1	0.000000000	0.878995000	-2.365992000
1	0.000000000	-0.878995000	-2.365992000
6	-1.263230000	1.235330000	0.065284000
6	1.263230000	1.235330000	0.065284000
6	0.000000000	1.223571000	0.972723000
1	1.257431000	2.159684000	-0.517901000
1	2.164238000	1.250241000	0.681449000
1	-1.257431000	2.159684000	-0.517901000
1	-2.164238000	1.250241000	0.681449000
6	1.263230000	-1.235330000	0.065284000
1	2.148290000	0.000000000	-1.464730000
6	-1.263230000	-1.235330000	0.065284000
1	-2.148290000	0.000000000	-1.464730000
6	0.000000000	0.000000000	1.815555000
1	0.000000000	2.122705000	1.592510000
6	0.000000000	-1.223571000	0.972723000
1	-1.257431000	-2.159684000	-0.517901000
1	-2.164238000	-1.250241000	0.681449000
1	2.164238000	-1.250241000	0.681449000
1	1.257431000	-2.159684000	-0.517901000
1	0.000000000	-2.122705000	1.592510000

E = −389.353457
ZVPE = 0.216449

Triplet adamantylidene (C_1)

6	0.000000000	0.000000000	-1.692488000
6	-1.257394000	0.000000000	-0.809648000
6	1.257394000	0.000000000	-0.809648000
1	0.000000000	0.879179000	-2.342086000
1	0.000000000	-0.879179000	-2.342086000
6	-1.263468000	1.262601000	0.068411000
6	1.263468000	1.262601000	0.068411000
6	0.000000000	1.267043000	0.972225000
1	1.269371000	2.157464000	-0.560736000
1	2.159395000	1.290387000	0.690516000
1	-1.269371000	2.157464000	-0.560736000
1	-2.159395000	1.290387000	0.690516000
6	1.263468000	-1.262601000	0.068411000
1	2.147548000	0.000000000	-1.443128000
6	-1.263468000	-1.262601000	0.068411000
1	-2.147548000	0.000000000	-1.443128000
6	0.000000000	0.000000000	1.729449000
1	0.000000000	2.146427000	1.618382000
6	0.000000000	-1.267043000	0.972225000
1	-1.269371000	-2.157464000	-0.560736000
1	-2.159395000	-1.290387000	0.690516000
1	2.159395000	-1.290387000	0.690516000
1	1.269371000	-2.157464000	-0.560736000
1	0.000000000	-2.146427000	1.618382000

E = −389.348668
ZVPE = 0.217606

2,4-Dehydroadamantane (C_s)

6	-0.601162000	-0.972154000	1.269792000
6	-1.398270000	-0.623127000	0.000000000
6	0.817722000	-0.387644000	1.184226000
1	-0.535131000	-2.055793000	1.393945000
1	-1.126550000	-0.580277000	2.144133000
6	-0.601162000	-0.972154000	-1.269792000
6	1.543968000	-1.054046000	0.000000000
6	0.817722000	-0.387644000	-1.184226000
1	-1.126550000	-0.580277000	-2.144133000
1	-0.535131000	-2.055793000	-1.393945000
1	1.468248000	-2.144177000	0.000000000
1	2.601183000	-0.783109000	0.000000000
6	-1.675278000	0.882086000	0.000000000
1	-2.345153000	-1.166305000	0.000000000
6	0.817722000	1.092486000	0.759008000
1	1.340490000	-0.522957000	2.130743000
6	0.817722000	1.092486000	-0.759008000
1	1.340490000	-0.522957000	-2.130743000
6	-0.367639000	1.632838000	0.000000000
1	-2.264069000	1.156476000	0.879733000
1	-2.264069000	1.156476000	-0.879733000
1	-0.457218000	2.712915000	0.000000000
1	1.437695000	1.783502000	1.312942000
1	1.437695000	1.783502000	-1.312942000

E = −389.438509
ZVPE = 0.219508

Adamantane alkene (C_1)

6	-0.232765000	-0.961118000	1.386891000
6	1.023964000	-0.140929000	1.047174000
6	-1.280287000	-0.818424000	0.269234000
1	-0.646231000	-0.619956000	2.338873000
1	0.026701000	-2.015596000	1.512351000
6	0.588390000	1.334338000	0.886238000
6	-1.682458000	0.650784000	0.053685000
6	-0.454995000	1.493820000	-0.298766000
1	1.451738000	1.976331000	0.707824000
1	0.123158000	1.669909000	1.816866000
1	-2.142891000	1.053976000	0.958962000
1	-2.428633000	0.715466000	-0.740347000
6	1.710641000	-0.716796000	-0.279575000
1	1.750124000	-0.216388000	1.859911000
6	-0.626063000	-1.290771000	-1.035922000
1	-2.164123000	-1.414448000	0.509080000
6	0.594133000	-0.423196000	-1.239276000
1	1.963257000	-1.771852000	-0.146601000
1	2.617427000	-0.148214000	-0.486072000
1	-1.314040000	-1.188131000	-1.874192000
1	-0.340946000	-2.342855000	-0.970184000
6	0.437459000	0.917443000	-1.386183000
1	-0.725008000	2.543236000	-0.426580000
1	1.361353000	1.487612000	-1.480889000

E = −389.381968
ZVPE = 0.217923

Adamantane TSA (C_1)

6	-0.238761000	-0.939719000	1.418141000
6	0.942029000	0.006425000	1.157122000
6	-1.163609000	-0.982748000	0.190991000
1	-0.809736000	-0.613990000	2.291263000
1	0.149135000	-1.935943000	1.643280000
6	0.497892000	1.396298000	0.688753000
6	-1.806310000	0.390545000	0.026081000
6	-0.729368000	1.427117000	-0.234675000
1	-2.378571000	0.663267000	0.916884000
1	-2.508031000	0.384909000	-0.811574000
1	0.573428000	2.245190000	1.362464000
1	1.293538000	1.729360000	-0.139418000
6	-0.379128000	-1.279871000	-1.095213000
1	-1.935029000	-1.740520000	0.341240000
6	1.743466000	-0.529520000	-0.037832000
1	1.563966000	0.097683000	2.048426000
6	0.374561000	1.184871000	-1.216773000
1	-1.146772000	2.430598000	-0.304893000
6	0.798166000	-0.294927000	-1.235128000
1	2.004234000	-1.585490000	0.062291000
1	2.667223000	0.037098000	-0.163951000
1	-1.049968000	-1.200283000	-1.953921000
1	0.010047000	-2.301600000	-1.093023000
1	1.332899000	-0.481102000	-2.167870000

E = −389.335187
ZVPE = 0.215103

Adamantane TSB (C_1)

6	0.150480000	-0.855666000	1.463762000
6	-0.999631000	0.091634000	1.082094000
6	1.170354000	-0.949439000	0.315577000
1	-0.240411000	-1.852578000	1.684239000
1	0.635805000	-0.496051000	2.374254000
6	-1.808899000	-0.474429000	-0.166300000
6	0.419280000	-1.442623000	-0.920675000
6	-0.698845000	-0.447792000	-1.193938000
1	0.006840000	-2.442065000	-0.764269000
1	1.085112000	-1.497588000	-1.783125000
1	-2.215662000	-1.468427000	0.042568000
1	-2.612401000	0.221440000	-0.404942000
6	1.770186000	0.425359000	-0.039609000
1	1.973071000	-1.636519000	0.594117000
6	-0.383390000	1.475196000	0.774748000
1	-1.691995000	0.188995000	1.921745000
6	-0.436136000	0.947177000	-1.336521000
1	-0.941576000	0.346646000	-2.325475000
6	0.666605000	1.406174000	-0.455267000
1	0.190473000	1.814928000	1.640467000
1	-1.150382000	2.216116000	0.564780000
1	2.492609000	0.311349000	-0.850721000
1	2.317800000	0.833949000	0.814328000
1	1.050696000	2.406256000	-0.651194000

E = 389.269783
ZVPE = 0.210986

PCU diazirine (C_1)

6	1.437438000	0.647004000	0.910232000
6	0.193413000	1.565733000	0.700762000
6	0.112033000	1.772197000	-0.811085000
6	0.620691000	0.418463000	-1.321524000
1	0.788617000	2.569444000	-1.125836000
1	-0.888606000	2.024072000	-1.160236000
6	1.905812000	0.172936000	-0.487834000
6	0.506778000	-0.513802000	1.378965000
6	-0.746752000	0.398284000	1.177346000
6	-1.437439000	-0.227751000	0.004051000
6	-0.323711000	-0.765140000	-0.852649000
6	0.560602000	-1.508667000	0.187566000
1	0.674509000	-0.953186000	2.357963000
1	2.225337000	0.978843000	1.580791000
1	0.788687000	0.388861000	-2.396118000
6	1.984512000	-1.348124000	-0.345299000
1	0.223326000	-2.516574000	0.417067000
1	2.149775000	-1.862074000	-1.293452000
1	2.744764000	-1.673489000	0.366538000
1	2.788948000	0.677289000	-0.874624000
1	-1.383275000	0.605978000	2.032767000
1	0.133263000	2.473930000	1.294538000
1	-0.662076000	-1.384124000	-1.680134000
7	-2.742762000	-0.890416000	0.105259000
7	-2.752030000	0.176733000	-0.499893000

E = −535.818690
ZVPE = 0.211683

PCU diazo (C_1)

6	1.533438000	0.796078000	0.748950000
6	0.224292000	1.615742000	0.536905000
6	0.008993000	1.629650000	-0.974891000
6	0.572037000	0.260507000	-1.370321000
1	0.587712000	2.428184000	-1.443301000
1	-1.036929000	1.764083000	-1.254209000
6	1.926786000	0.187310000	-0.619866000
6	0.717563000	-0.352760000	1.418257000
6	-0.607366000	0.458657000	1.221008000
6	-1.334281000	-0.343561000	0.188738000
6	-0.261889000	-0.916192000	-0.693459000
6	0.748568000	-1.475934000	0.350508000
1	0.981220000	-0.662463000	2.425489000
1	2.346187000	1.246049000	1.312389000
1	0.660506000	0.113606000	-2.445184000
6	2.114117000	-1.300102000	-0.314011000
1	0.493849000	-2.466494000	0.718676000
1	2.239255000	-1.912147000	-1.208613000
1	2.946248000	-1.497005000	0.363518000
1	2.745546000	0.690990000	-1.129690000
1	-1.182794000	0.736211000	2.099172000
1	0.148394000	2.584750000	1.024020000
1	-0.612988000	-1.646441000	-1.417828000
7	-2.591436000	-0.328359000	-0.049417000
7	-3.718529000	-0.348168000	-0.235632000

E = −535.837582
ZVPE = 0.211636

PCU singlet carbene (C_1)

6	-0.000171000	0.278538000	-1.375866000
6	1.440063000	0.199626000	-0.786596000
6	1.464489000	1.256512000	0.313504000
6	0.039187000	1.115245000	0.854807000
1	1.583087000	2.244963000	-0.139028000
1	2.246697000	1.118950000	1.055517000
6	-0.832640000	1.176534000	-0.420661000
6	-0.317073000	-1.158705000	-0.872817000
6	1.105449000	-1.285841000	-0.242171000
6	1.029546000	-1.101030000	1.212983000
6	-0.262043000	-0.387925000	1.377696000
6	-1.275620000	-0.910846000	0.310980000
1	-0.632992000	-1.901320000	-1.600394000
1	-0.109706000	0.493602000	-2.435205000
1	-0.228443000	1.837700000	1.621337000
6	-2.064491000	0.330855000	-0.104682000
1	-1.853420000	-1.776566000	0.628002000
1	-2.672127000	0.748644000	0.699497000
1	-2.704601000	0.160231000	-0.972128000
1	-1.010798000	2.191301000	-0.772201000
1	1.789836000	-2.046087000	-0.607435000
1	2.278555000	0.210973000	-1.475816000
1	-0.646266000	-0.360163000	2.394790000

E = −426.228274
ZVPE = 0.199734

PCU triplet carbene (C_1)

6	0.052631000	0.316319000	-1.370048000
6	1.477915000	0.257252000	-0.745113000
6	1.438600000	1.241157000	0.421114000
6	-0.015555000	1.105252000	0.883400000
1	1.632365000	2.259330000	0.075757000
1	2.161832000	1.000297000	1.201241000
6	-0.829800000	1.181261000	-0.432929000
6	-0.249810000	-1.135746000	-0.894953000
6	1.193198000	-1.230721000	-0.253330000
6	0.883916000	-1.220428000	1.179897000
6	-0.317965000	-0.386970000	1.389241000
6	-1.266191000	-0.923151000	0.260621000
1	-0.529755000	-1.881454000	-1.633883000
1	-0.037480000	0.534128000	-2.430976000
1	-0.315092000	1.840322000	1.628147000
6	-2.058798000	0.305830000	-0.181855000
1	-1.828795000	-1.807295000	0.548513000
1	-2.714242000	0.697518000	0.597621000
1	-2.651165000	0.131064000	-1.081869000
1	-1.015241000	2.195479000	-0.782150000
1	1.873880000	-1.988492000	-0.629522000
1	2.329228000	0.359916000	-1.413480000
1	-0.754387000	-0.401144000	2.384338000

E = −426.219023
ZVPE = 0.200109

PCU diradical (C_1)

6	0.350336000	-0.422225000	1.283511000
6	1.748058000	-0.174201000	0.847192000
6	1.885139000	-0.439843000	-0.618205000
6	0.457328000	-0.832390000	-1.041348000
1	2.584854000	-1.260306000	-0.821462000
1	2.260867000	0.428158000	-1.171336000
6	-0.150457000	-1.465162000	0.240862000
6	-0.654583000	0.810595000	1.023100000
6	0.039434000	1.993547000	0.347948000
6	0.071655000	1.718232000	-0.940655000
6	-0.519072000	0.391080000	-1.299919000
6	-1.543149000	0.248957000	-0.122387000
1	-1.203763000	1.068642000	1.927812000
1	0.261664000	-0.752395000	2.318403000
1	0.444832000	-1.506210000	-1.896158000
6	-1.650512000	-1.242841000	0.129280000
1	-2.460900000	0.810228000	-0.279426000
1	-2.106675000	-1.787171000	-0.699247000
1	-2.191356000	-1.483829000	1.046234000
1	0.185678000	-2.481547000	0.438950000
1	0.476333000	2.831370000	0.869521000
1	2.514828000	0.278972000	1.457729000
1	-0.971426000	0.339589000	-2.287301000

E = -426.217700
ZVPE = 0.197065

PCU homopentaprismane (C_{2v})

6	-1.203856000	0.786073000	0.173893000
6	-0.776071000	0.781892000	-1.319165000
6	0.000000000	1.141697000	1.071195000
6	1.203856000	0.786073000	0.173893000
6	0.776071000	0.781892000	-1.319165000
6	-1.203856000	-0.786073000	0.173893000
1	-2.137009000	1.280524000	0.432742000
6	1.203856000	-0.786073000	0.173893000
1	2.137009000	1.280524000	0.432742000
6	0.000000000	-1.141697000	1.071195000
6	-0.776071000	-0.781892000	-1.319165000
1	-1.314070000	1.389226000	-2.041372000
1	-2.137009000	-1.280524000	0.432742000
6	0.776071000	-0.781892000	-1.319165000
1	1.314070000	1.389226000	-2.041372000
1	2.137009000	-1.280524000	0.432742000
1	-1.314070000	-1.389226000	-2.041372000
1	1.314070000	-1.389226000	-2.041372000
6	0.000000000	0.000000000	2.107030000
1	0.000000000	2.155200000	1.468352000
1	-0.886008000	0.000000000	2.743905000
1	0.886008000	0.000000000	2.743905000
1	0.000000000	-2.155200000	1.468352000

E = −426.294063
ZVPE = 0.201342

PCU dialkene_1 (C_{2v})

6	1.172841000	0.804013000	0.080721000
6	1.172841000	-0.804013000	0.080721000
6	0.000000000	1.116482000	1.055130000
6	0.000000000	-1.116482000	1.055130000
6	-1.172841000	-0.804013000	0.080721000
6	-1.172841000	0.804013000	0.080721000
6	0.000000000	0.000000000	2.079551000
1	0.000000000	2.139609000	1.425801000
1	-0.886741000	0.000000000	2.716129000
1	0.886741000	0.000000000	2.716129000
1	0.000000000	-2.139609000	1.425801000
6	0.665554000	1.414136000	-1.204566000
1	2.138754000	1.184166000	0.409096000
6	-0.665554000	1.414136000	-1.204566000
1	-2.138754000	1.184166000	0.409096000
1	1.300427000	1.725933000	-2.021710000
1	-1.300427000	1.725933000	-2.021710000
6	-0.665554000	-1.414136000	-1.204566000
1	-2.138754000	-1.184166000	0.409096000
6	0.665554000	-1.414136000	-1.204566000
1	2.138754000	-1.184166000	0.409096000
1	-1.300427000	-1.725933000	-2.021710000
1	1.300427000	-1.725933000	-2.021710000

E = −426.329686
ZVPE = 0.200397

PCU dialkene_2 (C_{2v})

6	1.315162000	0.788300000	0.211841000
6	1.315162000	-0.788300000	0.211841000
6	0.000000000	1.146138000	0.942387000
6	0.000000000	-1.146138000	0.942387000
6	-1.315162000	-0.788300000	0.211841000
6	-1.315162000	0.788300000	0.211841000
6	0.000000000	0.000000000	1.995839000
1	0.000000000	2.154353000	1.353202000
1	-0.886567000	0.000000000	2.632033000
1	0.886567000	0.000000000	2.632033000
1	0.000000000	-2.154353000	1.353202000
6	1.505810000	-0.666723000	-1.290599000
1	2.157209000	-1.247003000	0.734476000
6	1.505810000	0.666723000	-1.290599000
1	2.157209000	1.247003000	0.734476000
6	-1.505810000	-0.666723000	-1.290599000
1	-2.157209000	-1.247003000	0.734476000
6	-1.505810000	0.666723000	-1.290599000
1	-2.157209000	1.247003000	0.734476000
1	-1.597068000	-1.405818000	-2.075465000
1	-1.597068000	1.405818000	-2.075465000
1	1.597068000	-1.405818000	-2.075465000
1	1.597068000	1.405818000	-2.075465000

E = –426.245913
ZVPE = 0.197615

PCU TSA (C_1)

6	-0.122249000	-0.689509000	-1.257807000
6	1.361383000	-0.340257000	-0.942864000
6	1.350384000	1.110698000	-0.438315000
6	-0.116218000	1.413449000	-0.100381000
1	1.977281000	1.866424000	-0.906566000
1	1.951298000	1.035549000	0.746927000
6	-1.000911000	0.562062000	-1.039967000
6	-0.215649000	-1.438769000	0.117919000
6	1.263399000	-1.069401000	0.419292000
6	1.264049000	0.155960000	1.371065000
6	-0.199686000	0.665711000	1.265045000
6	-1.133232000	-0.526362000	0.953737000
1	-0.489858000	-2.490577000	0.101013000
1	-0.334002000	-1.255430000	-2.160995000
1	-0.378564000	2.468197000	-0.060855000
6	-2.101190000	0.051386000	-0.092830000
1	-1.583219000	-0.996954000	1.825350000
1	-2.733589000	0.849532000	0.298457000
1	-2.738549000	-0.706086000	-0.551217000
1	-1.329217000	1.065024000	-1.947576000
1	1.955629000	-1.867125000	0.677740000
1	2.133477000	-0.609257000	-1.658511000
1	-0.531173000	1.270893000	2.106865000

E = –426.193898
ZVPE = 0.196384

PCU TSB (C_1)

6	0.057240000	-0.401048000	1.326049000
6	1.459426000	-0.600165000	0.911084000
6	1.569373000	-1.123954000	-0.440682000
6	0.126797000	-1.062270000	-0.947998000

1	2.068248000	-2.097225000	-0.504342000
1	2.191311000	-0.402876000	-1.030665000
6	-0.747454000	-1.279961000	0.314587000
6	-0.383673000	1.087181000	0.932290000
6	0.835249000	1.755095000	0.260854000
6	0.949732000	1.318998000	-1.003166000
6	-0.235513000	0.442302000	-1.329277000
6	-1.319942000	0.807689000	-0.263088000
1	-0.858428000	1.623383000	1.756064000
1	-0.123832000	-0.602359000	2.382035000
1	-0.087023000	-1.759010000	-1.755247000
6	-2.032116000	-0.494393000	0.062608000
1	-1.924893000	1.666418000	-0.545647000
1	-2.610763000	-0.890517000	-0.773634000
1	-2.678230000	-0.441670000	0.940005000
1	-0.857407000	-2.323496000	0.607713000
1	1.475990000	2.454070000	0.784802000
1	2.309641000	-0.434394000	1.558550000
1	-0.579329000	0.510832000	-2.359203000

E = −426.185930
ZVPE = 0.195423

Protoadamantane diazirine (C_1)

6	1.556672000	0.951218000	-0.991931000
6	0.436224000	1.572322000	-0.125938000
6	1.775825000	-0.556256000	-0.770028000
1	2.493182000	1.466885000	-0.762922000
1	1.345091000	1.144581000	-2.046632000
6	0.532209000	1.072986000	1.323392000
6	2.011187000	-0.828676000	0.720063000
6	0.637138000	-0.460712000	1.299983000
1	-0.345680000	1.400247000	1.884155000
1	1.406403000	1.488611000	1.827833000
1	2.818458000	-0.235890000	1.152169000
1	2.244387000	-1.883208000	0.881430000
6	-0.988616000	1.255182000	-0.625731000
1	0.563339000	2.655498000	-0.157378000
6	-1.452617000	-0.079797000	-0.094344000
1	-1.032633000	1.269550000	-1.718915000
1	-1.676528000	2.027989000	-0.276129000
6	-0.371908000	-1.062569000	0.255080000
1	0.471849000	-0.871674000	2.294614000
6	0.496401000	-1.366830000	-1.001936000
1	2.597570000	-0.899052000	-1.400329000
1	0.736188000	-2.429568000	-1.044334000
1	-0.017174000	-1.114273000	-1.929479000
1	-0.827309000	-1.972329000	0.641923000
7	-2.768463000	-0.582793000	-0.518143000
7	-2.741855000	-0.135574000	0.621334000

E = − 498.933811
ZVPE = 0.228617

Protoadamantane diazo (C_1)

6	1.684360000	0.974934000	-0.887935000
6	0.569115000	1.570439000	0.003773000
6	1.854387000	-0.551992000	-0.779931000
1	2.632594000	1.439570000	-0.604202000
1	1.500345000	1.255437000	-1.928147000
6	0.604883000	0.948398000	1.407121000

6	2.039461000	-0.947030000	0.689876000
6	0.661766000	-0.580613000	1.259974000
1	-0.280492000	1.256679000	1.966738000
1	1.475364000	1.291924000	1.968915000
1	2.851887000	-0.415677000	1.187363000
1	2.236853000	-2.017715000	0.773576000
6	-0.856181000	1.370461000	-0.561700000
1	0.743304000	2.645757000	0.067116000
6	-1.358557000	0.008775000	-0.154186000
1	-0.850177000	1.495693000	-1.650169000
1	-1.514576000	2.142814000	-0.158872000
6	-0.341514000	-1.056836000	0.140031000
1	0.449583000	-1.066030000	2.211543000
6	0.562384000	-1.306049000	-1.107253000
1	2.684993000	-0.867543000	-1.412857000
1	0.774710000	-2.371203000	-1.210675000
1	0.084622000	-0.976458000	-2.029003000
1	-0.846958000	-1.971748000	0.441698000
7	-2.615415000	-0.219127000	-0.030519000
7	-3.736394000	-0.412932000	0.076000000

E = – 498.951663
ZVPE = 0.228662

Singlet protoadamantylidene (C_1)

6	-0.047905000	-1.091050000	-1.285490000
6	1.081811000	-1.044487000	-0.236689000
6	-1.218447000	-0.142334000	-0.963140000
1	-0.441308000	-2.109274000	-1.344709000
1	0.361009000	-0.854750000	-2.271519000
6	0.503959000	-1.124858000	1.180778000
6	-1.769510000	-0.455903000	0.429297000
6	-0.577088000	-0.047489000	1.308142000
1	1.301234000	-0.969833000	1.911737000
1	0.070430000	-2.106809000	1.383020000
1	-2.053423000	-1.502003000	0.561170000
1	-2.646874000	0.159520000	0.639812000
6	1.886105000	0.263898000	-0.311031000
1	1.755797000	-1.884418000	-0.421220000
6	1.240814000	1.522211000	0.155154000
1	2.333708000	0.424869000	-1.296877000
1	2.761252000	0.205358000	0.355772000
6	-0.103507000	1.306871000	0.681223000
1	-0.856074000	0.102892000	2.350384000
6	-0.746423000	1.303537000	-0.791475000
1	-1.974435000	-0.213127000	-1.747459000
1	-1.567496000	2.017342000	-0.790586000
1	-0.054371000	1.630731000	-1.571069000
1	-0.488291000	2.157116000	1.240934000

E = – 389.346262
ZVPE = 0.216205

Triplet protoadamantylidene (C_1)

6	0.003075000	-1.180811000	-1.166201000
6	1.170136000	-0.955916000	-0.175336000
6	-1.274585000	-0.362431000	-0.884367000
1	-0.268417000	-2.239635000	-1.132781000
1	0.351417000	-0.983617000	-2.183269000
6	0.653217000	-0.876139000	1.268614000
6	-1.705349000	-0.560859000	0.573179000
6	-0.533367000	0.099214000	1.312216000

1	1.459807000	-0.547647000	1.926818000
1	0.327417000	-1.855977000	1.624203000
1	-1.835794000	-1.607413000	0.853227000
1	-2.646509000	-0.041388000	0.764060000
6	1.973919000	0.352003000	-0.446286000
1	1.863132000	-1.792667000	-0.284254000
6	1.209519000	1.471411000	0.125627000
1	2.157329000	0.465847000	-1.523931000
1	2.959726000	0.265389000	0.022844000
6	-0.221563000	1.391398000	0.432863000
1	-0.771784000	0.374567000	2.338210000
6	-1.027269000	1.147112000	-0.893422000
1	-2.047048000	-0.653175000	-1.598274000
1	-1.980109000	1.678776000	-0.861415000
1	-0.480324000	1.498773000	-1.767083000
1	-0.575245000	2.288267000	0.940326000

E = – 389.337959
ZVPE = 0.216687

2,4-Dehydroprotoadamantane (C_1)

6	1.067125000	-1.097572000	0.000000000
1	1.744752000	-1.953408000	0.000000000
6	0.197457000	-1.125786000	-1.269803000
6	-0.903239000	-0.056452000	-1.184214000
1	-1.438882000	0.011903000	-2.130734000
6	-0.353878000	1.317948000	-0.758997000
1	-0.673068000	2.189713000	-1.312945000
6	-0.353877000	1.317949000	0.758996000
6	0.947399000	1.379731000	-0.000001000
1	1.431436000	2.349421000	-0.000001000
6	1.883029000	0.197272000	-0.000001000
6	-1.824890000	-0.405793000	0.000001000
6	-0.903238000	-0.056451000	1.184215000
1	-1.438881000	0.011905000	2.130735000
6	0.197458000	-1.125785000	1.269804000
1	-0.265981000	-2.107535000	-1.394064000
1	0.830785000	-0.956751000	-2.144098000
1	-0.673067000	2.189714000	1.312944000
1	2.531597000	0.233534000	0.879738000
1	2.531596000	0.233534000	-0.879740000
1	-2.159069000	-1.446196000	0.000001000
1	-2.706108000	0.238077000	0.000001000
1	-0.265980000	-2.107534000	1.394066000
1	0.830786000	-0.956749000	2.144098000

E = –389.438505
ZVPE = 0.219514

Protoadamantane alkene (C_1)

6	-0.195767000	-1.120922000	-1.247252000
6	1.020098000	-1.125458000	-0.280256000
6	-1.331854000	-0.142960000	-0.865726000
1	-0.619591000	-2.129243000	-1.264794000
1	0.150801000	-0.907832000	-2.260498000
6	0.532127000	-1.100729000	1.182870000
6	-1.743662000	-0.365446000	0.593231000
6	-0.455973000	0.064212000	1.315526000
1	1.389652000	-0.986078000	1.845402000
1	0.030724000	-2.032892000	1.451676000

1	-2.024257000	-1.394252000	0.821864000
1	-2.588121000	0.277924000	0.848539000
6	1.911471000	0.087415000	-0.416635000
1	1.600924000	-2.028375000	-0.473046000
6	-0.874377000	1.316722000	-0.780452000
1	-2.160332000	-0.271281000	-1.564657000
6	1.460103000	1.247069000	0.046965000
6	0.026387000	1.334192000	0.495494000
1	-0.619418000	0.318381000	2.361593000
1	-1.747925000	1.958054000	-0.647376000
1	-0.335789000	1.663724000	-1.660351000
1	-0.140844000	2.241143000	1.073929000
1	2.910283000	-0.020708000	-0.820448000
1	2.062576000	2.146862000	0.025575000

E = - 389.445207
ZVPE = 0.219018

Protoadamantane alkene 2 (C_1)

6	-0.007732000	-0.216691000	1.529510000
6	-1.236774000	-0.676856000	0.638127000
6	1.379915000	0.097376000	0.847130000
1	0.189959000	-1.024773000	2.237987000
1	-0.314908000	0.645547000	2.126721000
6	-0.758032000	-1.454315000	-0.588372000
6	1.631427000	-0.954831000	-0.233058000
6	0.420970000	-0.637066000	-1.143662000
1	1.616189000	-1.982349000	0.129017000
1	2.588360000	-0.783988000	-0.726805000
6	-2.037176000	0.504479000	0.044577000
1	-1.893104000	-1.289458000	1.259845000
6	1.375406000	1.365999000	-0.072614000
1	2.151368000	0.150672000	1.617590000
6	-0.985419000	1.395685000	-0.568417000
6	0.204435000	0.869099000	-0.887206000
1	0.602415000	-0.846939000	-2.197562000
1	2.343010000	1.449208000	-0.572729000
1	1.156689000	2.294994000	0.449525000
1	-2.538930000	1.078384000	0.826594000
1	-2.820858000	0.128654000	-0.619134000
1	-1.574827000	-1.555732000	-1.304343000
1	-0.416467000	-2.461504000	-0.345631000
1	-1.011024000	2.440001000	-0.277172000

E = - 389.397887
ZVPE = 0.218131

Protoadamantane TSA (C_1)

6	-0.156976000	-1.046750000	1.328334000
6	-1.130722000	-1.021899000	0.137779000
6	1.030148000	-0.091791000	1.109108000
1	0.239063000	-2.054623000	1.472294000
1	-0.694475000	-0.781320000	2.242422000
6	-0.384012000	-1.170784000	-1.193829000
6	1.785226000	-0.523199000	-0.160540000
6	0.755445000	-0.146382000	-1.243978000
1	-1.082306000	-1.017586000	-2.019957000
1	0.029993000	-2.175970000	-1.305884000
1	2.053969000	-1.583016000	-0.165991000

1	2.701788000	0.058661000	-0.276657000
6	-1.871009000	0.321255000	0.106202000
1	-1.856401000	-1.830067000	0.254673000
6	-1.023756000	1.547654000	-0.160270000
1	-2.459987000	0.463016000	1.014972000
1	-2.593474000	0.319331000	-0.717445000
6	0.303416000	1.270163000	-0.791707000
1	1.194356000	-0.107997000	-2.240178000
6	0.592216000	1.316458000	0.705582000
1	1.660271000	-0.066293000	1.998162000
1	1.183898000	2.161919000	1.041063000
1	-0.500742000	1.810311000	1.068534000
1	0.724189000	2.075287000	-1.386096000

E = – 389.335091
ZVPE = 0.213953

Protoadamantane TSB (C_1)

6	-0.156871000	-1.109219000	-1.263161000
6	1.046108000	-1.082351000	-0.291078000
6	-1.311783000	-0.161162000	-0.878006000
1	-0.550890000	-2.128571000	-1.284508000
1	0.187342000	-0.887260000	-2.276250000
6	0.561395000	-1.099674000	1.170596000
6	-1.734627000	-0.419513000	0.570735000
6	-0.468207000	0.027151000	1.319608000
1	1.413349000	-0.965055000	1.838932000
1	0.099830000	-2.056457000	1.422213000
1	-1.997334000	-1.458875000	0.776130000
1	-2.594958000	0.201237000	0.828058000
6	1.884152000	0.187891000	-0.368040000
1	1.680518000	-1.944200000	-0.503785000
6	1.453804000	1.397333000	0.189511000
1	1.641523000	1.118907000	-1.148337000
1	2.938934000	0.074677000	-0.616149000
6	-0.017322000	1.335861000	0.543364000
1	-0.652145000	0.246094000	2.369951000
6	-0.870461000	1.301268000	-0.768306000
1	-2.129394000	-0.286999000	-1.589854000
1	-1.749523000	1.936824000	-0.657711000
1	-0.323052000	1.668330000	-1.636308000
1	-0.281321000	2.215847000	1.126291000

E = –389.331958
ZVPE = 0.213900

Protoadamantane TSB2 (C_1)

6	-0.143619000	-1.087187000	-1.280499000
6	1.058900000	-1.069510000	-0.301530000
6	-1.309053000	-0.156221000	-0.878190000
1	-0.526380000	-2.110193000	-1.326046000
1	0.200799000	-0.837368000	-2.286655000
6	0.564204000	-1.118148000	1.153239000
6	-1.731678000	-0.441413000	0.565162000
6	-0.467598000	0.001601000	1.320547000
1	1.410245000	-1.002257000	1.833646000
1	0.103488000	-2.081362000	1.379675000
1	-1.989182000	-1.485294000	0.754205000

1	-2.595140000	0.170882000	0.831869000
6	1.858877000	0.217549000	-0.416530000
1	1.702264000	-1.922796000	-0.522788000
6	1.366584000	1.454358000	0.017508000
1	2.340628000	0.712392000	0.626586000
1	2.779103000	0.194727000	-0.999103000
6	-0.031080000	1.327352000	0.569875000
1	-0.652848000	0.203083000	2.374374000
6	-0.880305000	1.308172000	-0.749688000
1	-2.124208000	-0.279529000	-1.593270000
1	-1.756609000	1.947031000	-0.640565000
1	-0.315508000	1.677208000	-1.606047000
1	-0.288047000	2.194151000	1.174754000

E = -389.333729
ZVPE = 0.213777

Protoadamantane TSC (C_1)

6	-0.017816000	-0.273992000	1.521988000
6	-1.266260000	-0.658991000	0.610659000
6	1.379222000	0.029142000	0.848775000
1	0.160644000	-1.126689000	2.181338000
1	-0.294988000	0.563500000	2.164592000
6	-0.800227000	-1.417315000	-0.631847000
6	1.600003000	-0.986161000	-0.273586000
6	0.391869000	-0.607033000	-1.160107000
1	-1.614816000	-1.490570000	-1.352865000
1	-0.475990000	-2.435570000	-0.409781000
1	1.560189000	-2.026388000	0.049733000
1	2.557832000	-0.820401000	-0.766696000
6	-2.041420000	0.561396000	0.049193000
1	-1.936879000	-1.265419000	1.220423000
6	-0.954771000	1.532244000	-0.325927000
1	-2.664407000	1.042351000	0.806677000
1	-2.719831000	0.243314000	-0.747247000
6	0.204504000	0.906647000	-0.802260000
1	0.563690000	-0.748710000	-2.225912000
6	1.422360000	1.337105000	-0.013566000
1	2.150616000	0.030645000	1.619805000
1	2.375179000	1.385396000	-0.543507000
1	1.239409000	2.251636000	0.540676000
1	-0.405428000	1.858658000	-1.477173000

E = -389.287129
ZVPE = 0.211716

d_2-Protoadamantane diazirine (C_1)

6	1.556672000	0.951218000	-0.991931000
6	0.436224000	1.572322000	-0.125938000
6	1.775825000	-0.556256000	-0.770028000
1	2.493182000	1.466885000	-0.762922000
1	1.345091000	1.144581000	-2.046632000
6	0.532209000	1.072986000	1.323392000
6	2.011187000	-0.828676000	0.720063000
6	0.637138000	-0.460712000	1.299983000
1	-0.345680000	1.400247000	1.884155000
1	1.406403000	1.488611000	1.827833000
1	2.818458000	-0.235890000	1.152169000
1	2.244387000	-1.883208000	0.881430000

6	-0.988616000	1.255182000	-0.625731000
1	0.563339000	2.655498000	-0.157378000
6	-1.452617000	-0.079797000	-0.094344000
1(Iso=2)	-1.032633000	1.269550000	-1.718915000
1(Iso=2)	-1.676528000	2.027989000	-0.276129000
6	-0.371908000	-1.062569000	0.255080000
1	0.471849000	-0.871674000	2.294614000
6	0.496401000	-1.366830000	-1.001936000
1	2.597570000	-0.899052000	-1.400329000
1	0.736188000	-2.429568000	-1.044334000
1	-0.017174000	-1.114273000	-1.929479000
1	-0.827309000	-1.972329000	0.641923000
7	-2.768463000	-0.582793000	-0.518143000
7	-2.741855000	-0.135574000	0.621334000

E = -498.940467

ZVPE = 0.221962

d_2-Protoadamantane diazo (C_1)

6	1.684360000	0.974934000	-0.887935000
6	0.569115000	1.570439000	0.003773000
6	1.854387000	-0.551992000	-0.779931000
1	2.632594000	1.439570000	-0.604202000
1	1.500345000	1.255437000	-1.928147000
6	0.604883000	0.948398000	1.407121000
6	2.039461000	-0.947030000	0.689876000
6	0.661766000	-0.580613000	1.259974000
1	-0.280492000	1.256679000	1.966738000
1	1.475364000	1.291924000	1.968915000
1	2.851887000	-0.415677000	1.187363000
1	2.236853000	-2.017715000	0.773576000
6	-0.856181000	1.370461000	-0.561700000
1	0.743304000	2.645757000	0.067116000
6	-1.358557000	0.008775000	-0.154186000
1(Iso=2)	-0.850177000	1.495693000	-1.650169000
1(Iso=2)	-1.514576000	2.142814000	-0.158872000
6	-0.341514000	-1.056836000	0.140031000
1	0.449583000	-1.066030000	2.211543000
6	0.562384000	-1.306049000	-1.107253000
1	2.684993000	-0.867543000	-1.412857000
1	0.774710000	-2.371203000	-1.210675000
1	0.084622000	-0.976458000	-2.029003000
1	-0.846958000	-1.971748000	0.441698000
7	-2.615415000	-0.219127000	-0.030519000
7	-3.736394000	-0.412932000	0.076000000

E = -498.958308

ZVPE = 0.222017

d_2-singlet protoadamantylidene (C_1)

6	-0.047905000	-1.091050000	-1.285490000
6	1.081811000	-1.044487000	-0.236689000
6	-1.218447000	-0.142334000	-0.963140000
1	-0.441308000	-2.109274000	-1.344709000
1	0.361009000	-0.854750000	-2.271519000
6	0.503959000	-1.124858000	1.180779000
6	-1.769510000	-0.455903000	0.429297000
6	-0.577088000	-0.047489000	1.308142000
1	1.301234000	-0.969833000	1.911737000
1	0.070430000	-2.106809000	1.383020000
1	-2.053423000	-1.502003000	0.561170000

1	-2.646874000	0.159520000	0.639812000
6	1.886105000	0.263898000	-0.311031000
1	1.755797000	-1.884418000	-0.421220000
6	1.240814000	1.522211000	0.155154000
1(Iso=2)	2.333708000	0.424869000	-1.296877000
1(Iso=2)	2.761252000	0.205358000	0.355772000
6	-0.103507000	1.306871000	0.681223000
1	-0.856074000	0.102892000	2.350384000
6	-0.746423000	1.303537000	-0.791475000
1	-1.974435000	-0.213128000	-1.747459000
1	-1.567496000	2.017342000	-0.790586000
1	-0.054371000	1.630731000	-1.571069000
1	-0.488291000	2.157116000	1.240933000

E = −389.352650

ZVPE = 0.209817

d_2-triplet protoadamantylidene (C_1)

6	0.003075000	-1.180811000	-1.166201000
6	1.170136000	-0.955916000	-0.175336000
6	-1.274585000	-0.362431000	-0.884367000
1	-0.268416000	-2.239635000	-1.132781000
1	0.351417000	-0.983617000	-2.183269000
6	0.653217000	-0.876139000	1.268614000
6	-1.705349000	-0.560859000	0.573179000
6	-0.533367000	0.099214000	1.312216000
1	1.459807000	-0.547646000	1.926818000
1	0.327418000	-1.855977000	1.624203000
1	-1.835793000	-1.607413000	0.853227000
1	-2.646509000	-0.041389000	0.764060000
6	1.973919000	0.352004000	-0.446286000
1	1.863133000	-1.792666000	-0.284254000
6	1.209519000	1.471411000	0.125627000
1(Iso=2)	2.157329000	0.465848000	-1.523931000
1(Iso=2)	2.959726000	0.265390000	0.022844000
6	-0.221563000	1.391398000	0.432863000
1	-0.771784000	0.374567000	2.338210000
6	-1.027269000	1.147112000	-0.893422000
1	-2.047048000	-0.653176000	-1.598274000
1	-1.980109000	1.678775000	-0.861415000
1	-0.480324000	1.498773000	-1.767083000
1	-0.575246000	2.288267000	0.940326000

E = −389.344465

ZVPE = 0.210182

d_2-2,4-Dehydroprotoadamantane (C_1)

6	1.067125000	-1.097572000	0.000000000
1	1.744751000	-1.953408000	0.000000000
6	0.197457000	-1.125786000	-1.269803000
6	-0.903239000	-0.056452000	-1.184214000
1	-1.438883000	0.011904000	-2.130734000
6	-0.353877000	1.317949000	-0.758997000
1	-0.673067000	2.189714000	-1.312944000
6	-0.353876000	1.317949000	0.758996000
6	0.947399000	1.379731000	-0.000001000
1	1.431437000	2.349420000	-0.000001000
6	1.883029000	0.197271000	-0.000001000
6	-1.824890000	-0.405792000	0.000001000
6	-0.903238000	-0.056451000	1.184215000
1	-1.438881000	0.011905000	2.130735000
6	0.197458000	-1.125785000	1.269804000
1	-0.265982000	-2.107535000	-1.394064000
1	0.830784000	-0.956751000	-2.144098000
1	-0.673066000	2.189715000	1.312943000
1(Iso=2)	2.531597000	0.233533000	0.879738000
1(Iso=2)	2.531596000	0.233533000	-0.879740000
1	-2.159070000	-1.446196000	0.000001000
1	-2.706108000	0.238078000	0.000001000
1	-0.265980000	-2.107534000	1.394066000
1	0.830786000	-0.956749000	2.144098000

E = -389,445184

ZVPE = 0,212836

d_2-Protoadamantane alkene (C_1)

6	-0.195766000	-1.120922000	-1.247252000
6	1.020099000	-1.125457000	-0.280256000
6	-1.331854000	-0.142961000	-0.865726000
1	-0.619590000	-2.129243000	-1.264794000
1	0.150802000	-0.907832000	-2.260498000
6	0.532128000	-1.100729000	1.182870000
6	-1.743662000	-0.365447000	0.593231000
6	-0.455973000	0.064212000	1.315526000
1	1.389653000	-0.986077000	1.845402000
1	0.030725000	-2.032892000	1.451676000
1	-2.024256000	-1.394253000	0.821864000
1	-2.588121000	0.277922000	0.848539000
6	1.911471000	0.087416000	-0.416635000
1	1.600925000	-2.028374000	-0.473046000
6	-0.874378000	1.316721000	-0.780452000
1	-2.160332000	-0.271282000	-1.564657000
6	1.460102000	1.247070000	0.046965000
6	0.026386000	1.334192000	0.495494000
1	-0.619418000	0.318381000	2.361593000
1	-1.747926000	1.958053000	-0.647376000
1	-0.335790000	1.663724000	-1.660351000
1	-0.140845000	2.241143000	1.073929000
1(Iso=2)	2.910283000	-0.020706000	-0.820448000
1(Iso=2)	2.062575000	2.146863000	0.025575000

E = -389.451612
ZVPE = 0.212613

d_2-Protoadamantane alkene 2 (C_1)

6	-0.007732000	-0.216691000	1.529510000
6	-1.236774000	-0.676856000	0.638127000
6	1.379915000	0.097376000	0.847130000
1	0.189959000	-1.024773000	2.237987000
1	-0.314908000	0.645547000	2.126721000
6	-0.758032000	-1.454315000	-0.588372000
6	1.631427000	-0.954831000	-0.233058000
6	0.420970000	-0.637066000	-1.143662000
1	1.616189000	-1.982349000	0.129017000
1	2.588360000	-0.783988000	-0.726805000
6	-2.037176000	0.504479000	0.044577000
1	-1.893104000	-1.289458000	1.259845000
6	1.375406000	1.365999000	-0.072614000
1	2.151368000	0.150672000	1.617590000
6	-0.985419000	1.395685000	-0.568417000
6	0.204435000	0.869099000	-0.887206000
1	0.602415000	-0.846939000	-2.197562000
1	2.343010000	1.449208000	-0.572729000
1	1.156689000	2.294994000	0.449525000
1(Iso=2)	-2.538930000	1.078384000	0.826594000
1(Iso=2)	-2.820858000	0.128654000	-0.619134000
1	-1.574827000	-1.555732000	-1.304343000
1	-0.416467000	-2.461504000	-0.345631000
1	-1.011024000	2.440001000	-0.277172000

E = - 389.404519
ZVPE = 0.211499

d_2-Protoadamantane TSA (C_1)

6	-0.156976000	-1.046750000	1.328334000
6	-1.130722000	-1.021899000	0.137779000
6	1.030148000	-0.091791000	1.109108000
1	0.239064000	-2.054623000	1.472294000
1	-0.694475000	-0.781320000	2.242422000
6	-0.384012000	-1.170784000	-1.193829000
6	1.785226000	-0.523199000	-0.160540000
6	0.755445000	-0.146382000	-1.243978000
1	-1.082306000	-1.017586000	-2.019957000
1	0.029994000	-2.175970000	-1.305884000
1	2.053969000	-1.583016000	-0.165991000
1	2.701788000	0.058662000	-0.276657000
6	-1.871009000	0.321254000	0.106202000
1	-1.856401000	-1.830068000	0.254673000
6	-1.023756000	1.547654000	-0.160270000
1	-2.459987000	0.463015000	1.014972000
1	-2.593474000	0.319330000	-0.717445000
6	0.303416000	1.270163000	-0.791707000
1	1.194356000	-0.107997000	-2.240178000
6	0.592216000	1.316458000	0.705582000
1	1.660271000	-0.066293000	1.998162000
1	1.183897000	2.161919000	1.041063000
1	-0.500742000	1.810311000	1.068534000
1	0.724189000	2.075287000	-1.386096000

E = -389.341719
ZVPE = 0.207325

d_2-Protoadamantane TSB (C_1)

6	-0.156871000	-1.109219000	-1.263161000
6	1.046108000	-1.082351000	-0.291078000
6	-1.311783000	-0.161162000	-0.878006000
1	-0.550890000	-2.128571000	-1.284508000
1	0.187342000	-0.887260000	-2.276250000
6	0.561395000	-1.099674000	1.170596000
6	-1.734627000	-0.419513000	0.570735000
6	-0.468207000	0.027151000	1.319608000
1	1.413349000	-0.965055000	1.838932000
1	0.099830000	-2.056457000	1.422213000
1	-1.997334000	-1.458875000	0.776130000
1	-2.594958000	0.201236000	0.828058000
6	1.884152000	0.187891000	-0.368040000
1	1.680518000	-1.944200000	-0.503785000
6	1.453804000	1.397333000	0.189511000
1(Iso=2)	1.641523000	1.118907000	-1.148337000
1(Iso=2)	2.938934000	0.074677000	-0.616149000
6	-0.017322000	1.335861000	0.543364000
1	-0.652145000	0.246094000	2.369951000
6	-0.870461000	1.301268000	-0.768306000
1	-2.129394000	-0.286999000	-1.589854000
1	-1.749523000	1.936824000	-0.657711000
1	-0.323052000	1.668330000	-1.636308000
1	-0.281321000	2.215847000	1.126291000

E = −389.337438
ZVPE = 0.208419

d_2-Protoadamantane TSB2 (C_1)

6	-0.143618000	-1.087187000	-1.280499000
6	1.058901000	-1.069509000	-0.301530000
6	-1.309053000	-0.156222000	-0.878190000
1	-0.526379000	-2.110193000	-1.326046000
1	0.200799000	-0.837368000	-2.286655000
6	0.564205000	-1.118148000	1.153239000
6	-1.731678000	-0.441414000	0.565162000
6	-0.467598000	0.001601000	1.320547000
1	1.410246000	-1.002256000	1.833646000
1	0.103489000	-2.081362000	1.379675000
1	-1.989181000	-1.485295000	0.754205000
1	-2.595140000	0.170881000	0.831869000
6	1.858877000	0.217550000	-0.416530000
1	1.702265000	-1.922795000	-0.522788000
6	1.366583000	1.454359000	0.017508000
1	2.340628000	0.712393000	0.626586000
1	2.779103000	0.194729000	-0.999103000
6	-0.031081000	1.327352000	0.569875000
1	-0.652848000	0.203083000	2.374374000
6	-0.880306000	1.308172000	-0.749688000
1	-2.124208000	-0.279530000	-1.593270000
1	-1.756610000	1.947030000	-0.640565000
1	-0.315509000	1.677208000	-1.606047000
1	-0.288048000	2.194151000	1.174754000

E = −389.339187
ZVPE = 0.208319

d_2-Protoadamantane TSC (C_1)

6	-0.017816000	-0.273992000	1.521988000
6	-1.266260000	-0.658991000	0.610659000
6	1.379222000	0.029142000	0.848775000
1	0.160644000	-1.126689000	2.181338000
1	-0.294988000	0.563500000	2.164592000
6	-0.800227000	-1.417315000	-0.631847000
6	1.600003000	-0.986161000	-0.273586000
6	0.391869000	-0.607033000	-1.160107000
1	-1.614816000	-1.490570000	-1.352865000
1	-0.475990000	-2.435570000	-0.409781000
1	1.560189000	-2.026388000	0.049733000
1	2.557832000	-0.820401000	-0.766696000
6	-2.041420000	0.561396000	0.049193000
1	-1.936879000	-1.265419000	1.220423000
6	-0.954771000	1.532244000	-0.325927000
1	-2.664407000	1.042351000	0.806677000
1	-2.719831000	0.243314000	-0.747247000
6	0.204504000	0.906647000	-0.802260000
1	0.563690000	-0.748710000	-2.225912000
6	1.422360000	1.337105000	-0.013566000
1	2.150616000	0.030645000	1.619805000
1	2.375179000	1.385396000	-0.543507000
1	1.239409000	2.251636000	0.540676000
1	-0.405428000	1.858658000	-1.477173000

E = −389.293688
ZVPE = 0.205157

Dinitrogen N_2 ($D_{\infty h}$)

7	0.000000000	0.000000000	0.545450000
7	0.000000000	0.000000000	-0.545450000

E = − 109.568426
ZVPE = 0.005588

References

1. Becke, A. D., Density-functional exchange-energy approximation with correct asymptotic behavior. *Phys. Rev. A.* **1988,** *38* (6), 3098.
2. Becke, A., Density-Functional Thermochemistry. III. The Role of Exact Exchange. J. Chem. Phys., 98: 5648-5652. 1993.
3. Fernandez-Ramos, A.; Ellingson, B. A.; Garrett, B. C.; Truhlar, D. G., Variational Transition State Theory with Multidimensional Tunneling. In *Reviews in Computational Chemistry*, 2007; pp 125-232.
4. Šumanovac, T.; Alešković, M.; Šekutor, M.; Matković, M.; Baron, T.; Mlinarić-Majerski, K.; Bohne, C.; Basarić, N., Photoelimination of nitrogen from adamantane and pentacycloundecane (PCU) diazirines: a spectroscopic study and supramolecular control. *Photochem. Photobiol. Sci.* **2019,** *18* (7), 1806-1822.
5. Majerski, Z.; Hamersak, Z., Rearrangement of bridgehead alcohols to polycyclic ketones by fragmentation-cyclization: 4-protoadamantanone (tricyclo-[4.3.1.03,8]decan-4-one). *Org. Synth.* **1979,** *59* (147).
6. Egunlusi, A. O.; Malan, S. F.; Omoruyi, S. I.; Ekpo, O. E.; Palchykov, V. A.; Joubert, J., Open and rearranged norbornane derived polycyclic cage molecules as potential neuroprotective agents through attenuation of MPP+- and calcium overload-induced excitotoxicity in neuroblastoma SH-SY5Y cells. *Eur. J. Med. Chem.* **2020,** *204*, 112617.
7. Gaidai, A. V.; Volochnyuk, D. M.; Shishkin, O. V.; Fokin, A. A.; Levandovskiy, I. A.; Shubina, T. E., D_3-Trishomocubane-4-carboxylic Acid as a New Chiral Building Block: Synthesis and Absolute Configuration. *Synthesis* **2012,** *44* (05), 810-816.
8. Guerra-Navarro, N. A.; Palacios-Grijalva, L. N.; Angeles-Beltrán, D.; Negrón-Silva, G. E.; Lomas-Romero, L.; González-Zamora, E.; Gaviño-Ramírez, R.; Navarrete-Bolaños, J., Synthesis of New Pentacyclo[5.4.0.02,6.03,10.05,9]undecane-8,11-dione (PCU) Cyanosilylated Derivatives Using Sulphated Zirconia and Hydrotalcite as Catalysts in Microwave-Assisted Reactions under Solvent Free Conditions. *Molecules* **2011,** *16* (8), 6561-6576.
9. Eaton, P. E.; Cassar, L.; Hudson, R. A.; Hwang, D. R., Synthesis of homopentaprismane and homohypostrophene and some comments on the mechanism of metal ion catalyzed rearrangements of polycyclic compounds. *J. Org. Chem.* **1976,** *41* (8), 1445-1448.

Full Citations for Electronic Structure Codes

Gaussian 16

M. J. Frisch, G. W. Trucks, H. B. Schlegel, G. E. Scuseria, M. A. Robb, J. R. Cheeseman, G. Scalmani, V. Barone, G. A. Petersson, H. Nakatsuji, X. Li, M. Caricato, A. V. Marenich, J. Bloino, B. G. Janesko, R. Gomperts, B. Mennucci, H. P. Hratchian, J. V. Ortiz, A. F. Izmaylov, J. L. Sonnenberg, D. Williams-Young, F. Ding, F. Lipparini, F. Egidi, J. Goings, B. Peng, A. Petrone, T. Henderson, D. Ranasinghe, V. G. Zakrzewski, J. Gao, N. Rega, G. Zheng, W. Liang, M. Hada, M. Ehara, K. Toyota, R. Fukuda, J. Hasegawa, M. Ishida, T. Nakajima, Y. Honda, O. Kitao, H. Nakai, T. Vreven, K. Throssell, J. A. Montgomery, Jr., J. E. Peralta, F. Ogliaro, M. J. Bearpark, J. J. Heyd, E. N. Brothers, K. N. Kudin, V. N. Staroverov, T. A. Keith, R. Kobayashi, J. Normand, K. Raghavachari, A. P. Rendell, J. C. Burant, S. S. Iyengar, J. Tomasi, M. Cossi, J. M. Millam, M. Klene, C. Adamo, R. Cammi, J. W. Ochterski, R. L. Martin, K. Morokuma, O. Farkas, J. B. Foresman, D. J. Fox, Gaussian 16 Revision C.01, **2016**, Gaussian Inc., Wallingford.

Polyrate

J. Zheng, J. L. Bao, R. Meana-Pañeda, S. Zhang, B. J. Lynch, J. C. Corchado, Y.-Y. Chuang, P. L. Fast, W.-P. Hu, Y.-P. Liu, G. C. Lynch, K. A. Nguyen, C. F. Jackels, A. Fernandez Ramos, B. A. Ellingson, V. S. Melissas, J. Villà, I. Rossi, E. L. Coitiño, J. Pu, T. V. Albu, A. Ratkiewicz, R. Steckler, B. C. Garrett, A. D. Isaacson, D. G. Truhlar, Polyrate Version 2017-C, **2017**, University of Minnesota, Minneapolis.
